# Supplementary material for: Basonuclin-2 promotes fracture repair through NuRD-dependent chromatin remodeling in periosteal stem cells
Source: EMBO J. 2025 Dec 22;45(4):1060–76. doi: 10.1038/s44318-025-00664-1 (PMC12910080; doi:10.1038/s44318-025-00664-1)
Supplement: Supplementary file 1 — Appendix [file 44318_2025_664_MOESM1_ESM.pdf]

Appendix for

Basonuclin-2 promotes fracture repair through NuRD-dependent chromatin remodeling in periosteal stem cells

Contents

Appendix Figure S1. RNAseq analysis of SSCs from 3 dpf callus and periosteum 1

Appendix Figure S2. Bnc2 is expressed in periosteal precursor cells 2

Appendix Figure S3. Drilling repair of Prx1-cre_Bnc2 mice is normal 3

Appendix Figure S4. Prx1-creER，Bnc2 mice shows significant impairment in fracture healing 4

Appendix Figure S5. Fracture healing in Ocn-cre_Bnc2 mice is normal 5

Appendix Figure S6. Ocn-creER+ cells are not involved in cartilage formation during fracture healing 6

Appendix Figure S7. The periosteum thickening in 3 dpf in Prx1-cre_Bnc2 mice is hindered 7

Appendix Figure S8. The percentage of Proliferative BNC2+ periosteal cells increased significantly after fracture 8

Appendix Figure S9. Bnc2 promotes proliferation ability of periosteal cell 9

Appendix Figure S10. Prx1-cre_Bnc2 did not show cartilage phenotype 10

Appendix Figure S11. Flow sorting of tdTomato-positive cells in Prx1-cre; Bnc2$^{f/f}$; Rosa26-LSL-tdTomato mice in 3 dpf 11

Appendix Figure S12. working model 12

Appendix Figure S13. Prx1-cre; Bnc2$^{f/f}$ mice show decreased trabecular bone volume and thickened cortical bone 13

Appendix Figure S1

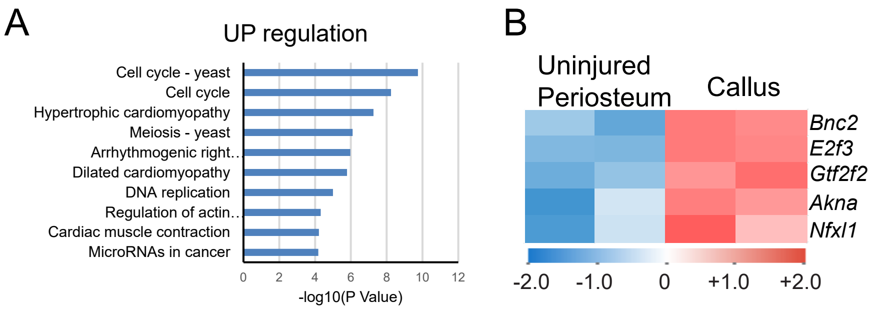

Appendix Figure S1. RNAseq analysis of SSCs from 3 dpf callus and periosteum.
(**A**) GO analysis of up-regulated genes in callus SSCs on 3 dpf. (**B**) Heat maps of transcription factor expression in up-regulated genes in callus SSCs on 3 dpf.

Appendix Figure S2

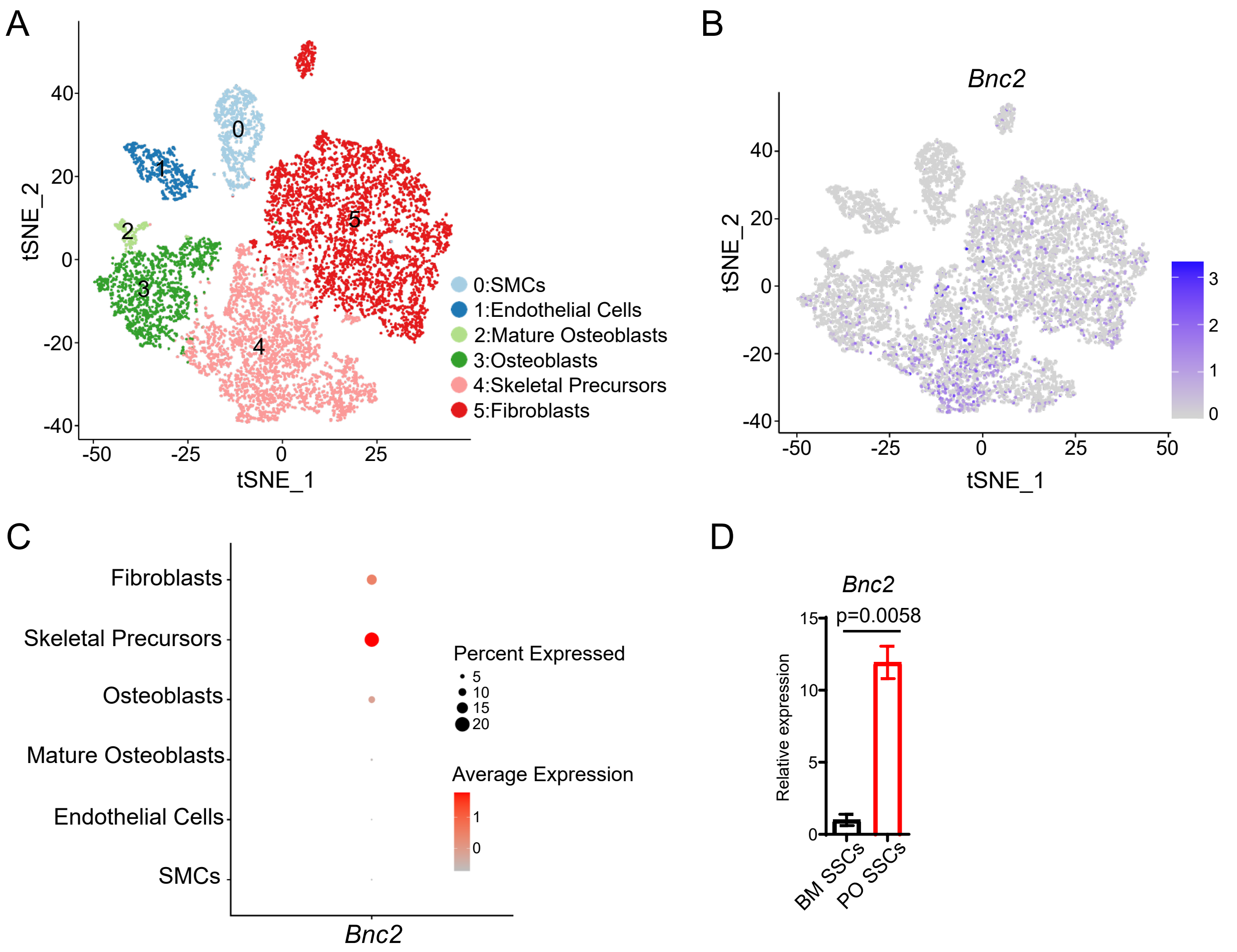

Appendix Figure S2. *Bnc2* is expressed in periosteal precursor cells.
(**A-C**) A cluster of single-cell sequencing of *Prx1-cre; Rosa26-LSL-tdTomato* positive periosteal cells (**A**), and the expression of *Bnc2* was specifically enriched in skeletal precursors (**B** and **C**). (**D**) The relative expression of *Bnc2* in SSCs in periosteum and bone marrow. Data are presented as the means ± SEM. n = 2. Unpaired t test.

Appendix Figure S3

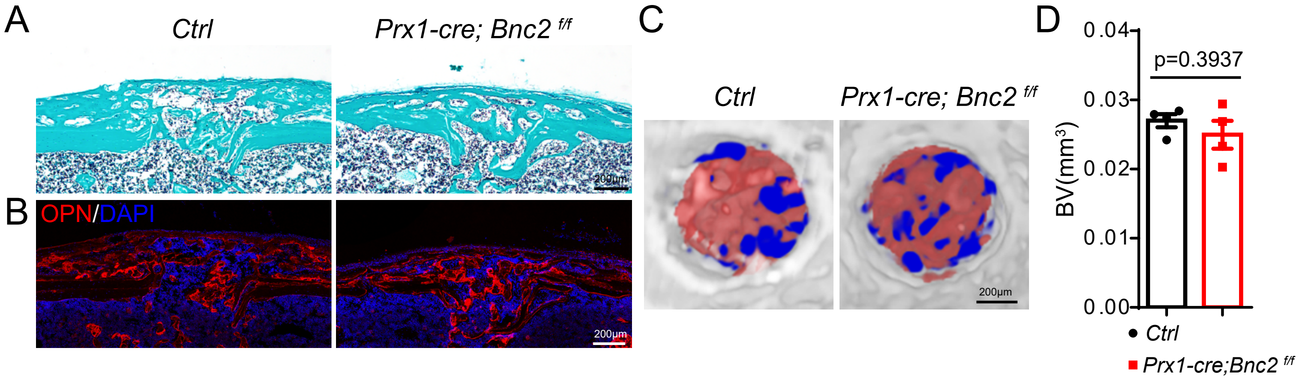

Appendix Figure S3. Drilling repair of *Prx1-cre; Bnc2$^{f/f}$* mice is normal.
(**A**) SOFG staining of *Prx1-cre; Bnc2$^{f/f}$* mice after 14 days of drilling. (**B**) OPN immunofluorescence staining of *Prx1-cre; Bnc2$^{f/f}$* mice 14 days after drilling. (**C** and **D**) micro-CT images of *Prx1-cre; Bnc2$^{f/f}$* mice after 14 days of drilling (**C**) and quantitative statistics of bone volume at the traumatic region (**D**). Data are presented as the means ± SEM. n = 4. Unpaired t test.

Appendix Figure S4

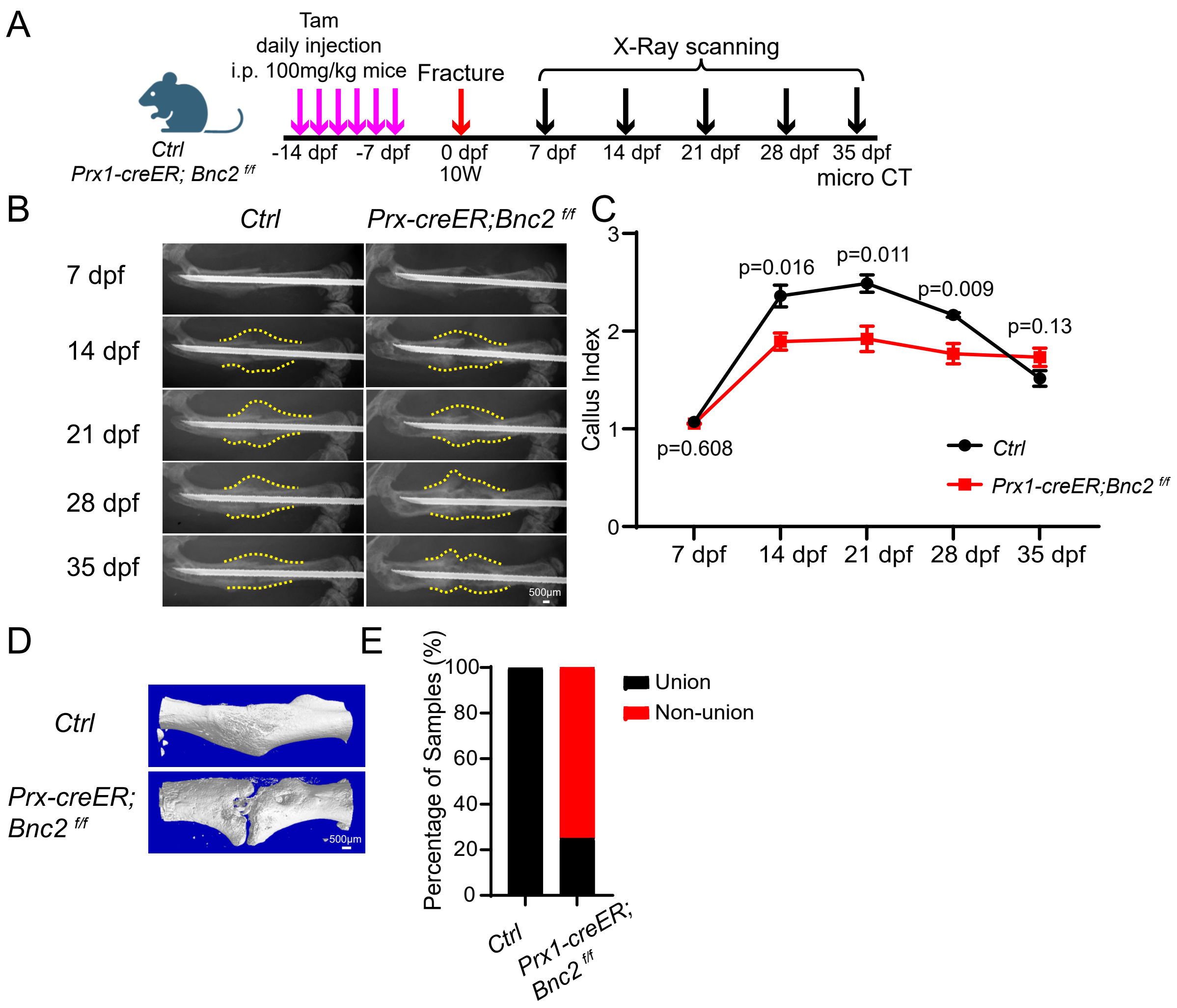

Appendix Figure S4. *Prx1-creER;Bnc2*$^{f/f}$ mice shows significant impairment in fracture healing. (**A**) Schematic diagram of *Prx1-creER; Bnc2*$^{f/f}$ mice fracture model. (**B** and **C**) X-Ray results (**B**) and quantitative statistics of callus index (**C**) in *Prx1-creER; Bnc2*$^{f/f}$ mice at different stages after fracture. Data are presented as the means ± SEM. n = 4. Unpaired t test. (**D**) microCT scanning results of *Prx1-creER; Bnc2*$^{f/f}$ mice on 35 dpf. (**E**) Quantitative statistics of percentage of union and non-union fracture samples on 35 dpf in *Prx1-creER; Bnc2*$^{f/f}$ mice. n = 4.

Appendix Figure S5

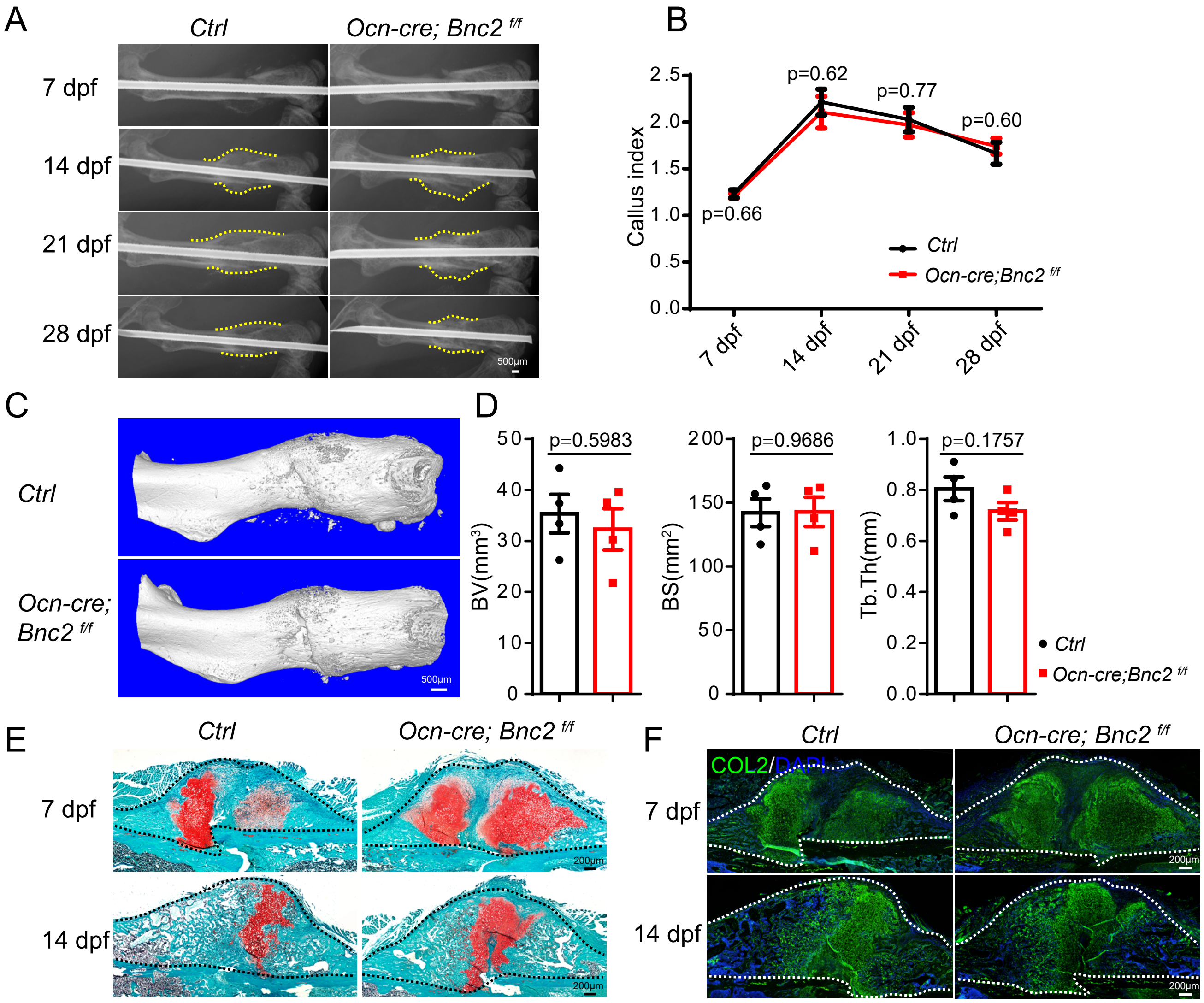

Appendix Figure S5. Fracture healing in *Ocn-cre; Bnc2$^{f/f}$* mice is normal.

(**A** and **B**) X-Ray results (**A**) and quantitative statistics of callus index (**B**) in *Ocn-cre; Bnc2$^{f/f}$* mice at different stages after fracture. Data are presented as the means ± SEM. n = 4. Unpaired t test. (**C** and **D**) micro-CT scanning results (**C**) and quantitative statistics of new bone formation at the fracture site (**D**) of *Ocn-cre; Bnc2$^{f/f}$* mice on 28 dpf. Data are presented as the means ± SEM. n = 4. Unpaired t test. (**E**) SOFG staining at different stages after fracture in *Ocn-cre; Bnc2$^{f/f}$* mice. (**F**) COL2 immunofluorescence staining of *Ocn-cre; Bnc2$^{f/f}$* mice on 7 dpf and 14 dpf.

Appendix Figure S6

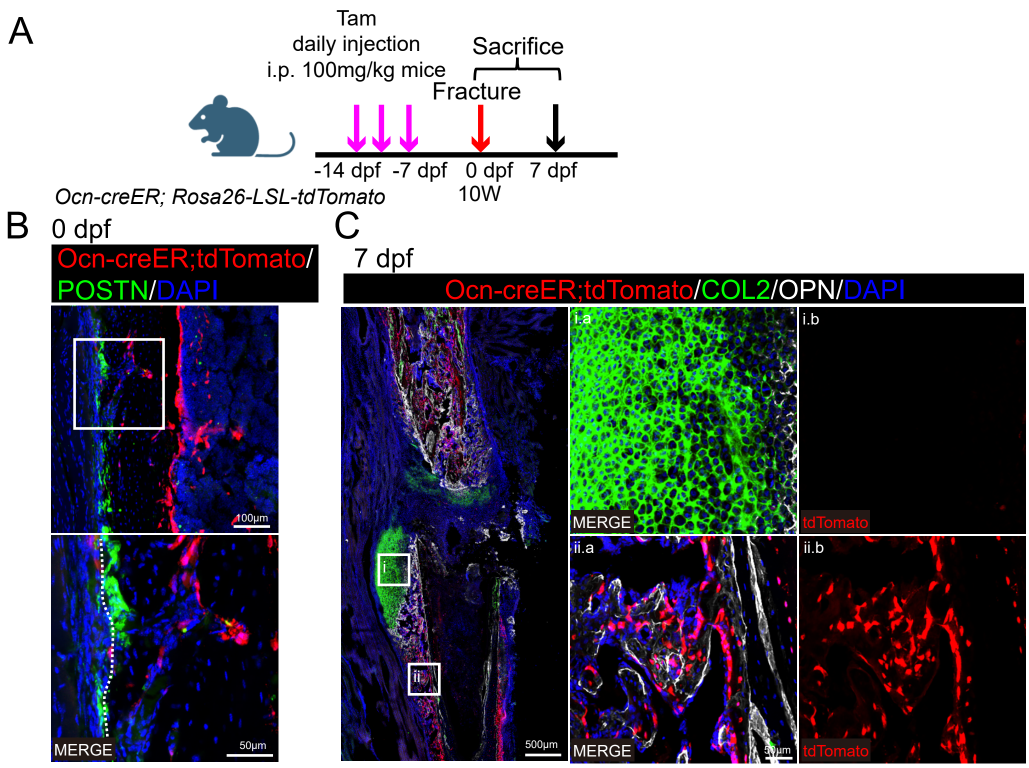

Appendix Figure S6. ***Ocn-creER***$^{+}$ **cells are not involved in cartilage formation during fracture healing.**

(**A**) Schematic diagram of *Ocn-creER; Rosa26-LSL-tdTomato* mouse fracture model. (**B**) Lineage tracing of 8-weeks-old *Ocn-creER; Rosa26-LSL-tdTomato* mice at 7 days after tamoxifen induction. (**C**) Slides on 7 dpf revealed *Ocn-creER; Rosa26-LSL-tdTomato*$^{+}$ cells did not label chondrocytes.

Appendix Figure S7

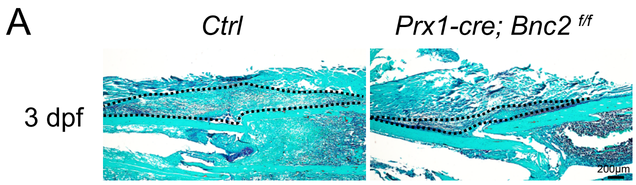

Appendix Figure S7. The periosteum thickening on 3 dpf in *Prx1-cre; Bnc2$^{f/f}$* mice is hindered.
(A) SOFG staining on 3 dpf in *Prx1-cre; Bnc2$^{f/f}$* mice.

Appendix Figure S8

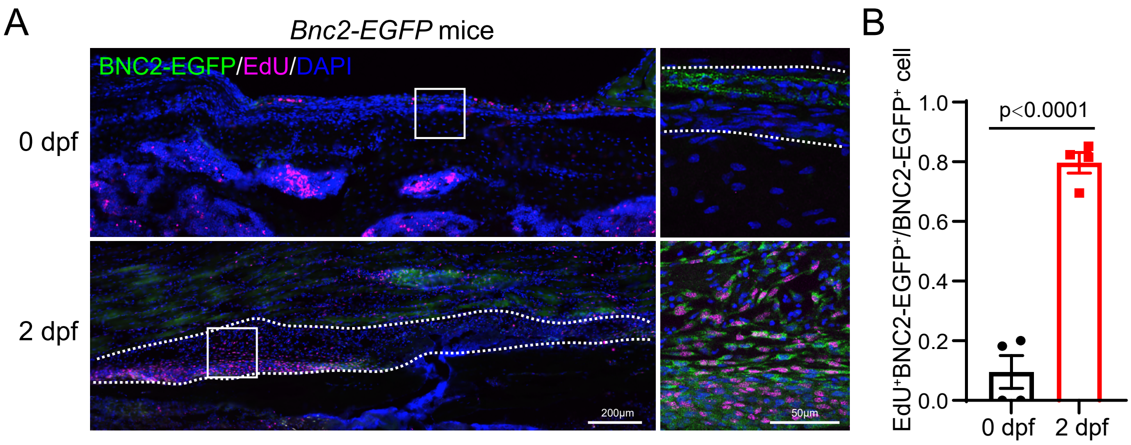

Appendix Figure S8. The percentage of proliferative BNC2^{+} periosteal cells increased significantly after fracture.

(A and **B)** The expression of BNC2-EGFP on 0 dpf and 2 dpf and the quantitative statistics of the proportion of proliferative BNC2-EGFP positive cells. Data are presented as the means ± SEM. n = 4. Unpaired t test.

Appendix Figure S9

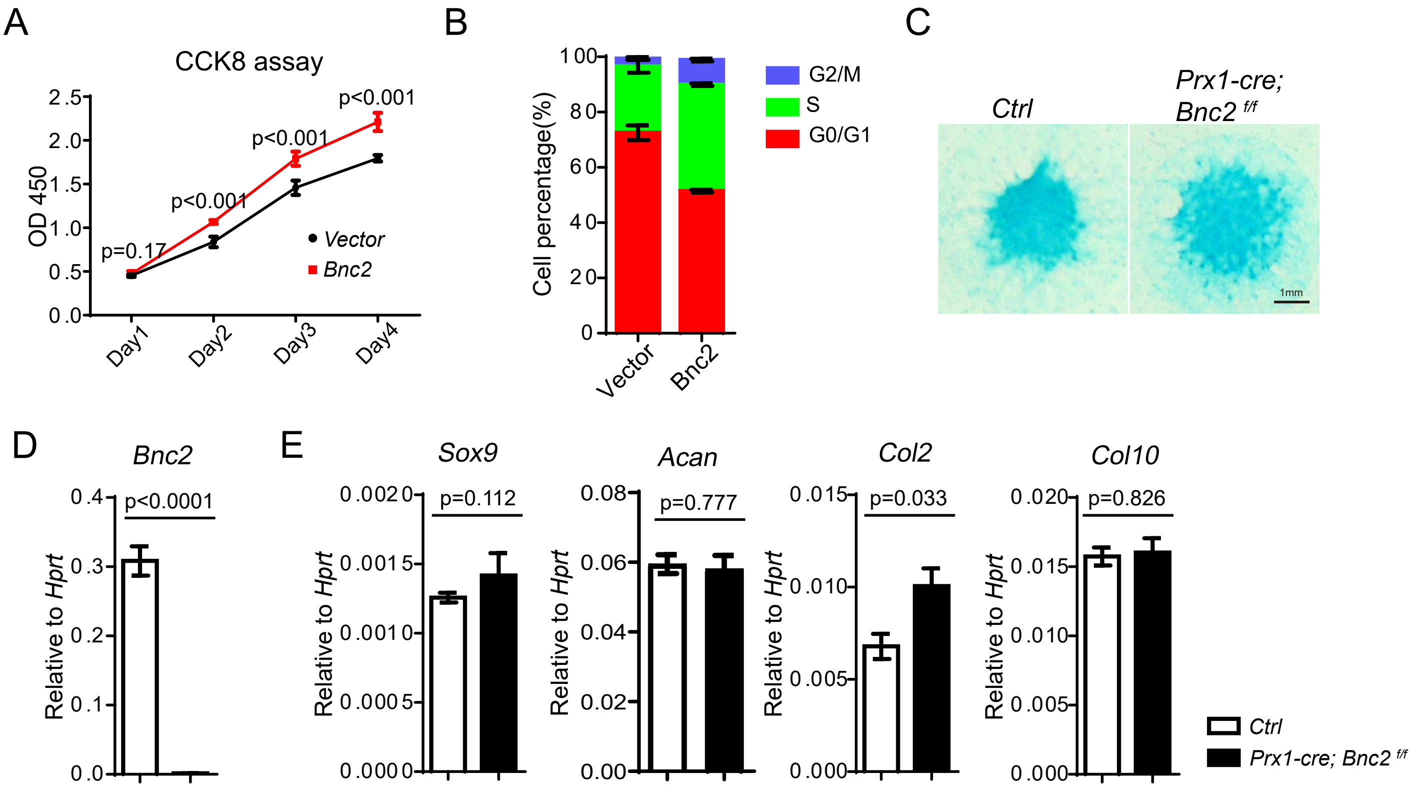

Appendix Figure S9. *Bnc2* promotes proliferation ability of periosteal cell.
(**A**) CCK8 assay of periosteal cells with *Bnc2* overexpression. Data are presented as the means ± SEM. n = 6. Unpaired t test. (**B**) Cell cycle analysis of periosteal cells with *Bnc2* overexpression. Data are presented as the means ± SEM. n = 3. Unpaired t test. (**C**) Alcian blue staining for chondrogenic differentiation of periosteal cell in *Prx1-cre; Bnc2$^{f/f}$* mice. (**D** and **E**) The expression of *Bnc2* and chondrogenic marker genes during chondrogenic differentiation of periosteal cell in *Prx1-cre; Bnc2$^{f/f}$* mice. Data are presented as the means ± SEM. n = 4. Unpaired t test.

Appendix Figure S10

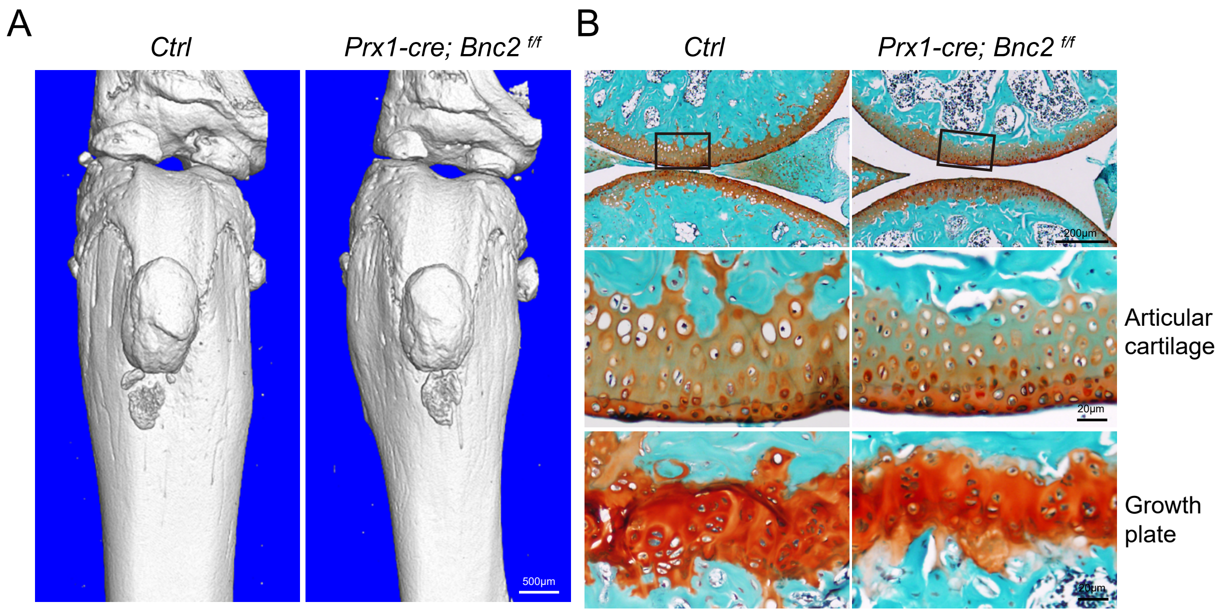

Appendix Figure S10. *Prx1-cre;Bnc2$^{f/f}$* mice did not show cartilage phenotype.
(**A**) Micro-CT scan images of 6-month-old *Prx1-cre; Bnc2$^{f/f}$* mice. (**B**) SOFG staining of 6-month-old *Prx1-cre; Bnc2$^{f/f}$* mice.

Appendix Figure S11

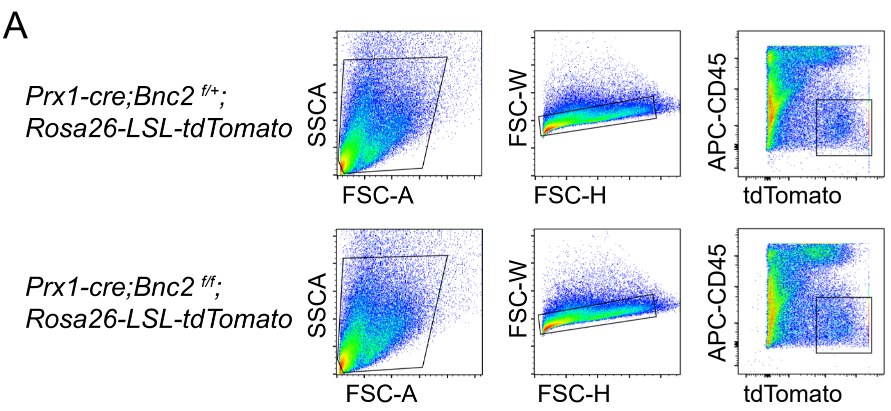

Appendix Figure S11. Flow sorting of tdTomato^{+} cells in *Prx1-cre; Bnc2$^{f/f}$; Rosa26-LSL-tdTomato* mice on 3 dpf.

(A) Flow sorting strategies of CD45^{-} tdTomato^{+} cells in *Prx1-cre; Bnc2$^{f/f}$; Rosa26-LSL-tdTomato* mice on 3 dpf.

Appendix Figure S12

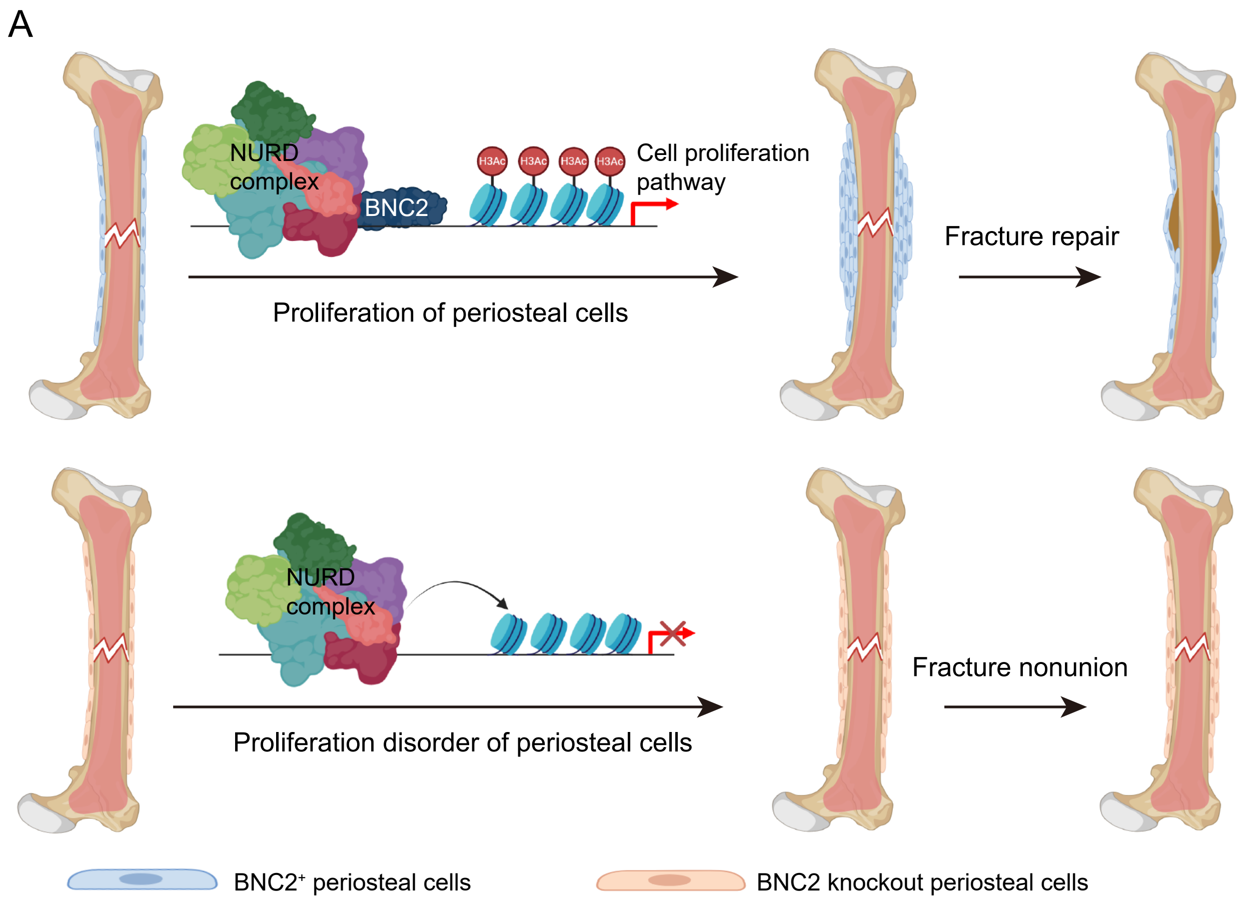

Appendix Figure S12. Working model of BNC2 promoting fracture repair.

(A) BNC2 regulates the proliferation of periosteal cells through interaction with the NuRD complex, thereby promoting fracture repair in mice.

Appendix Figure S13

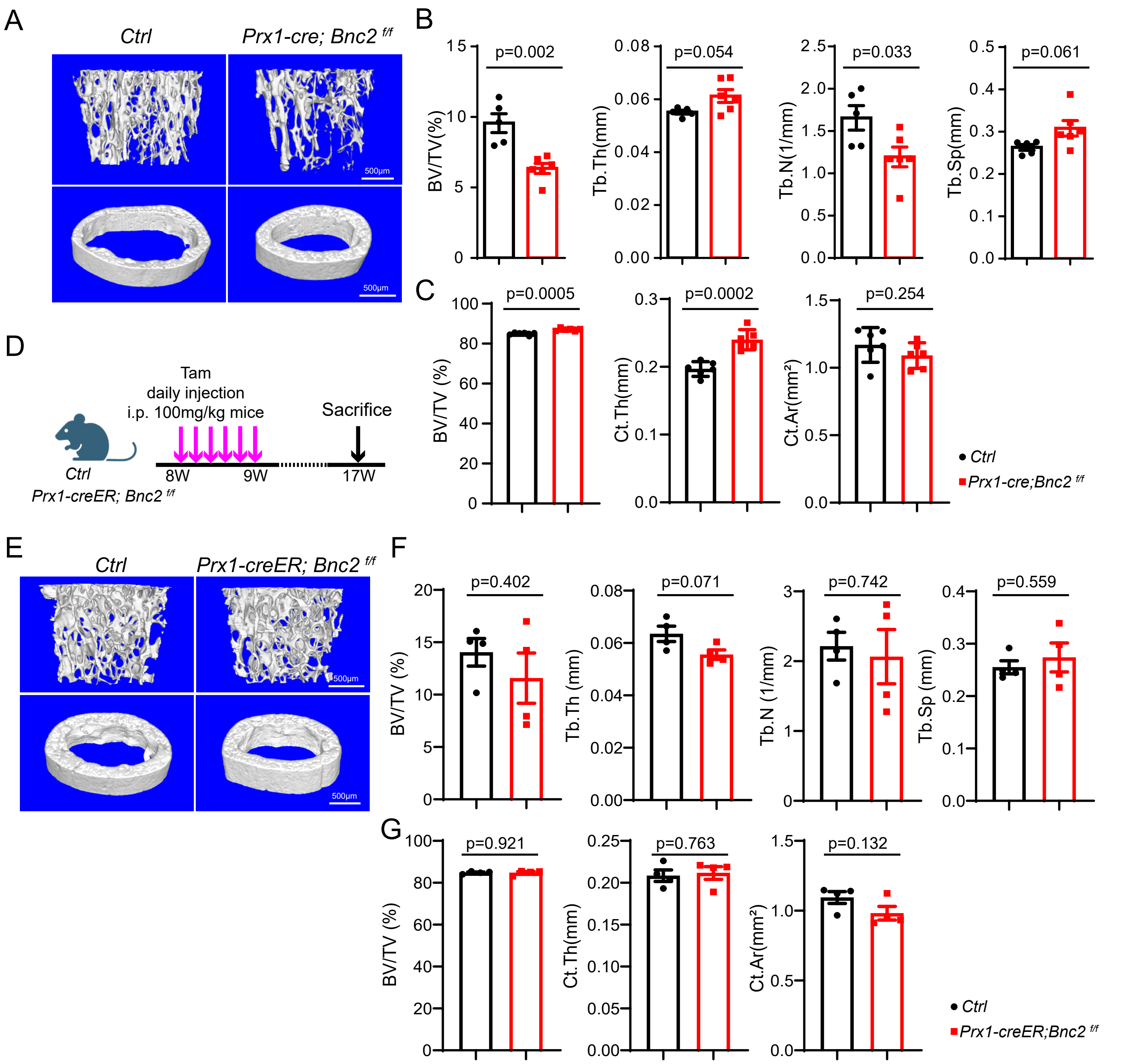

Appendix Figure S13. ***Prx1-cre;Bnc2$^{f/f}$*** **but not** ***Prx1-creER;Bnc2$^{f/f}$*** **mice show decreased trabecular bone volume and thickened cortical bone.**

(**A**) Micro-CT scan images of cancellous bone and cortical bone of *Prx1-cre; Bnc2$^{f/f}$* mice. (**B** and **C**) Quantitative statistics of trabecular bone mass (**B**) and cortical bone (**C**) in *Prx1-cre; Bnc2$^{f/f}$* mice. Data are presented as the means ± SEM. n = 6. Unpaired t test. (**D**) Schematic diagram of tamoxifen induction strategy in *Prx1-creER; Bnc2$^{f/f}$* mice. (**E**) Micro-CT scan images of cancellous bone and cortical bone of *Prx1-creER; Bnc2$^{f/f}$* mice. (**F** and **G**) Quantitative statistics of trabecular bone mass (**F**) and cortical bone (**G**) in *Prx1-creER; Bnc2$^{f/f}$* mice. Data are presented as the means ± SEM. n = 4. Unpaired t test.
