# Supplementary figures and images for: Basonuclin-2 promotes fracture repair through NuRD-dependent chromatin remodeling in periosteal stem cells
Source: EMBO J. 2025 Dec 22;45(4):1060–76. doi: 10.1038/s44318-025-00664-1 (PMC12910080; doi:10.1038/s44318-025-00664-1)

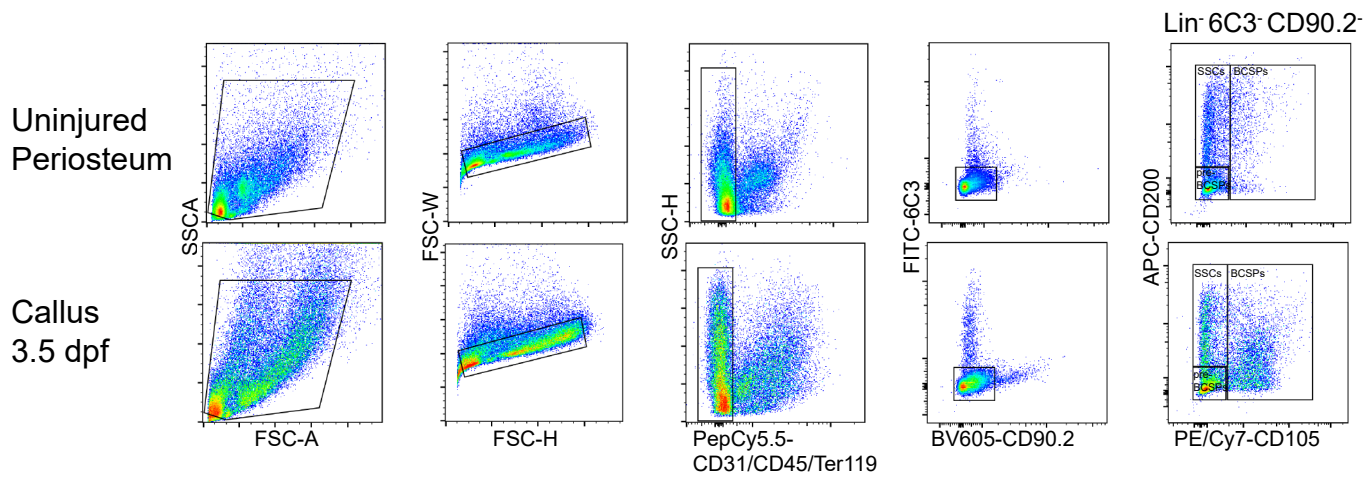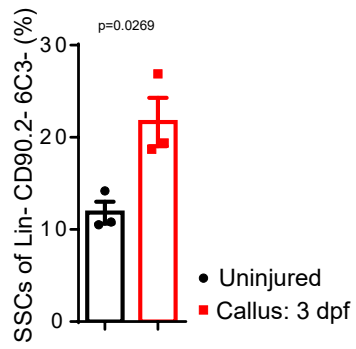

Supplement: Supplementary file 3 — Source data Fig. 1 [file 44318_2025_664_MOESM3_ESM.zip › Figure 1/1A/WT Fra PO,Callus - SSC sorting.pdf]

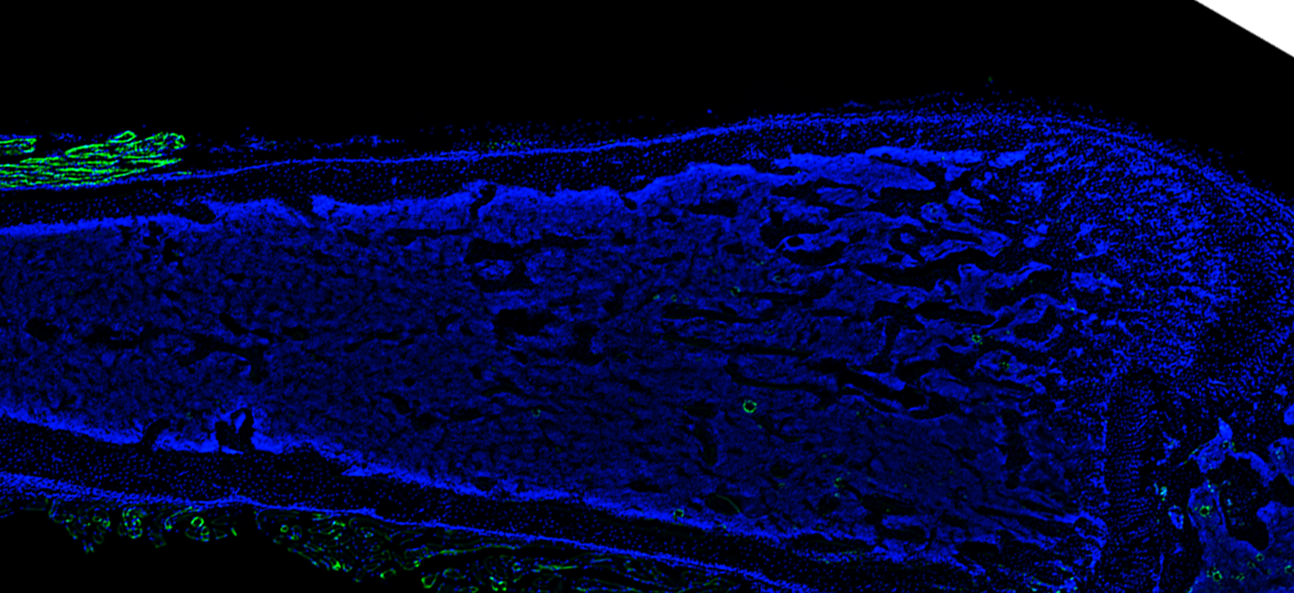

Supplement: Supplementary file 3 — Source data Fig. 1 [file 44318_2025_664_MOESM3_ESM.zip › Figure 1/1E/0 dpf_left.tif]

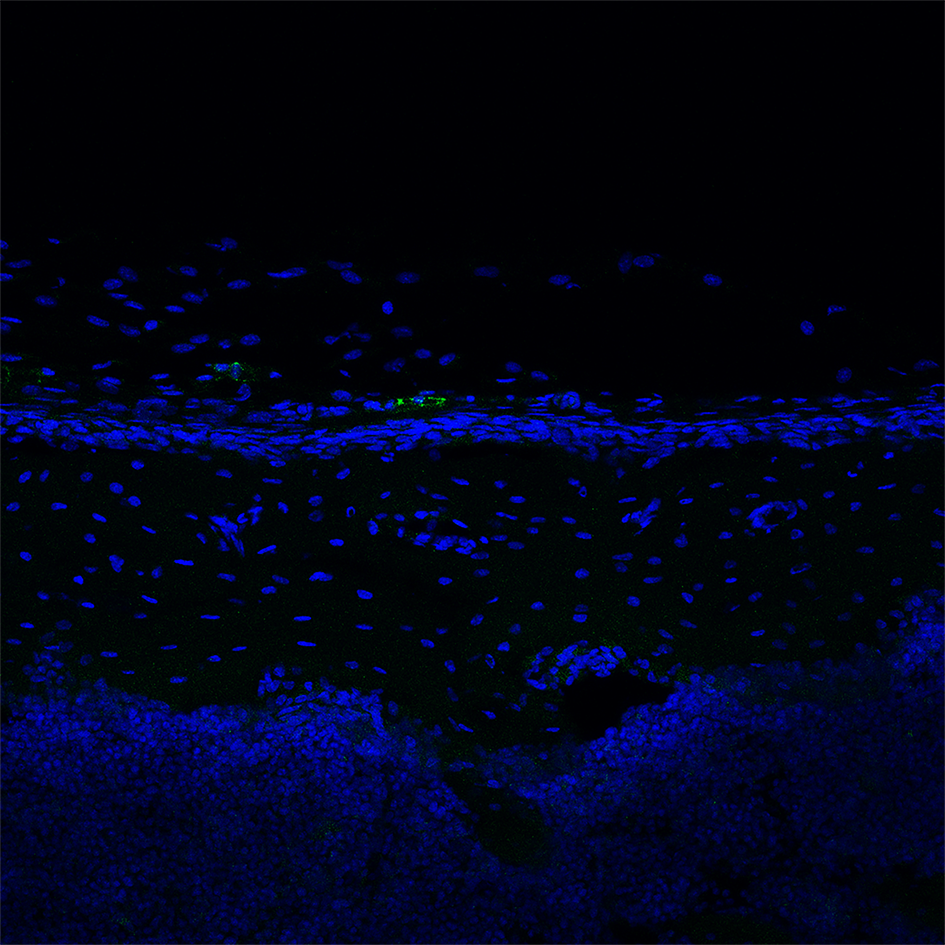

Supplement: Supplementary file 3 — Source data Fig. 1 [file 44318_2025_664_MOESM3_ESM.zip › Figure 1/1E/0 dpf_right.tif]

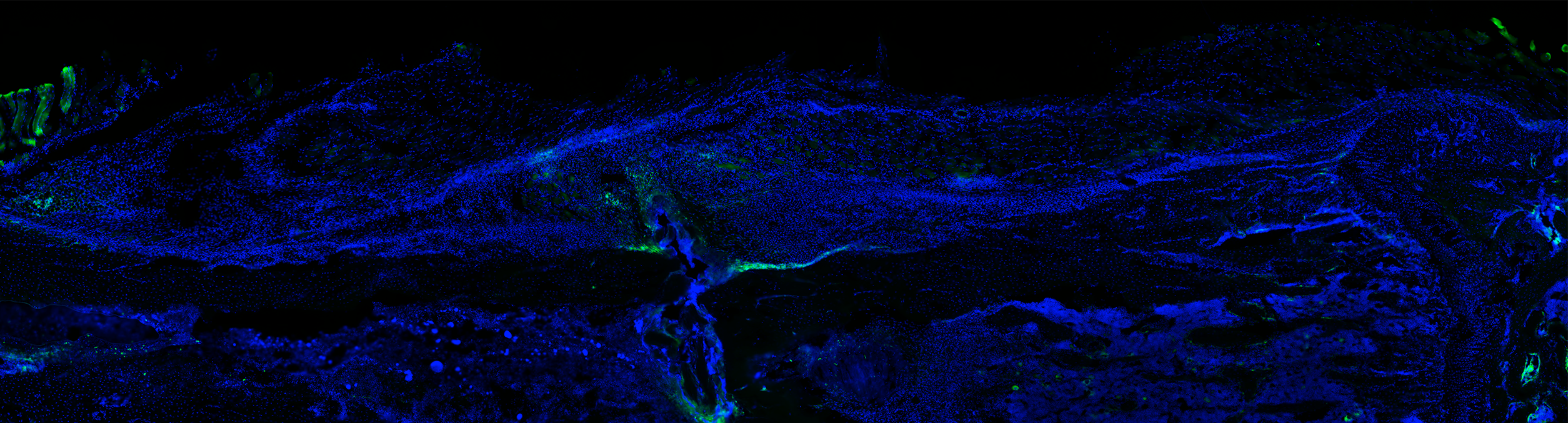

Supplement: Supplementary file 3 — Source data Fig. 1 [file 44318_2025_664_MOESM3_ESM.zip › Figure 1/1E/3 dpf_left.tif]

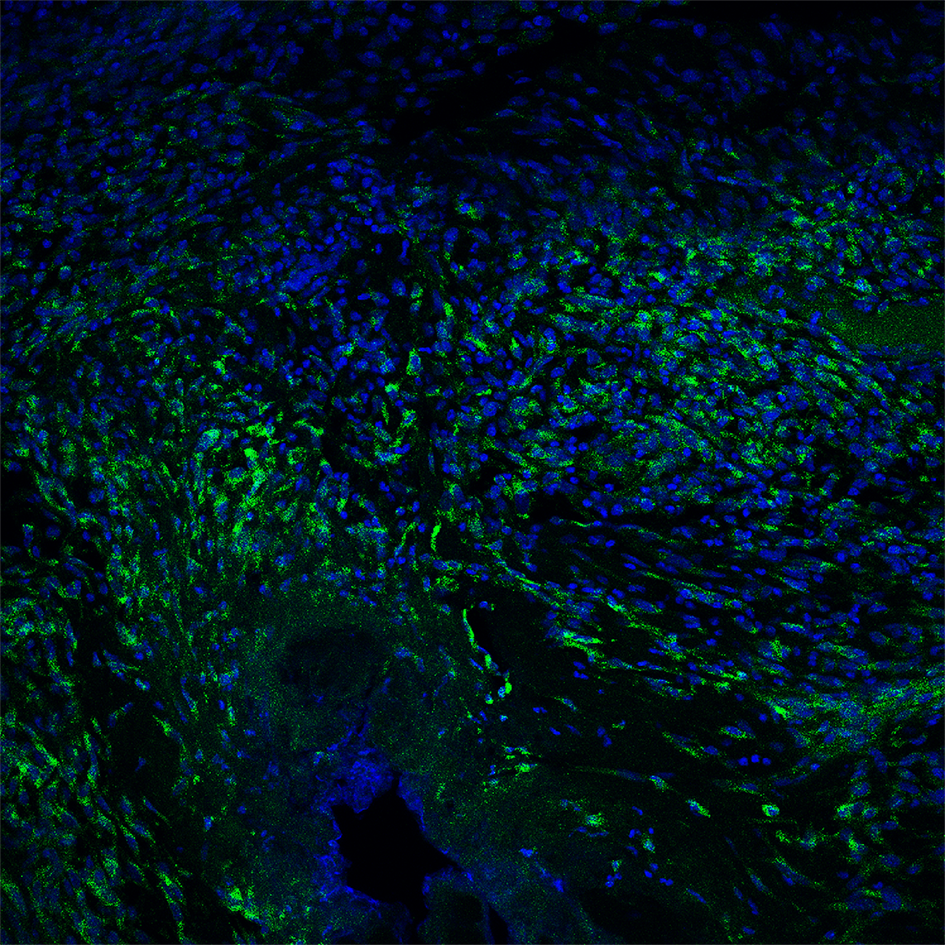

Supplement: Supplementary file 3 — Source data Fig. 1 [file 44318_2025_664_MOESM3_ESM.zip › Figure 1/1E/3 dpf_right.tif]

# Bnc2-GFP mice

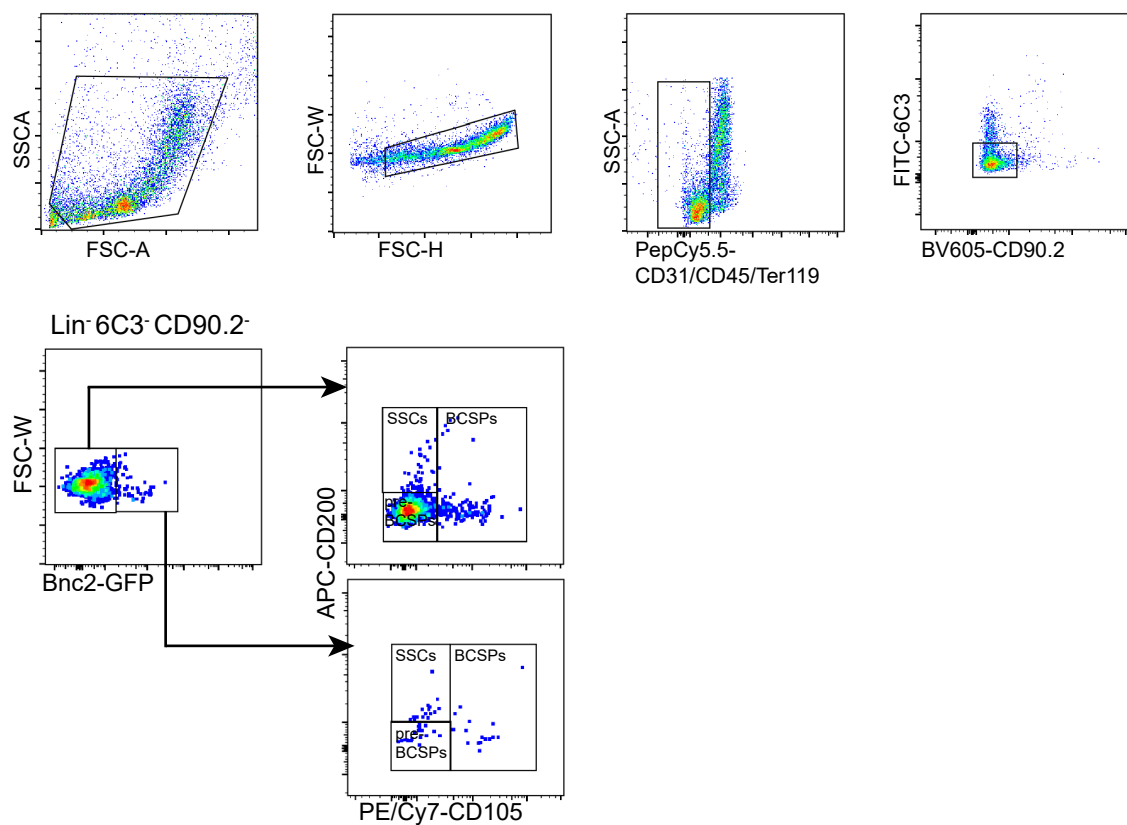

Supplement: Supplementary file 3 — Source data Fig. 1 [file 44318_2025_664_MOESM3_ESM.zip › Figure 1/1G/Bnc2-GFP mice - SSC flow.pdf]

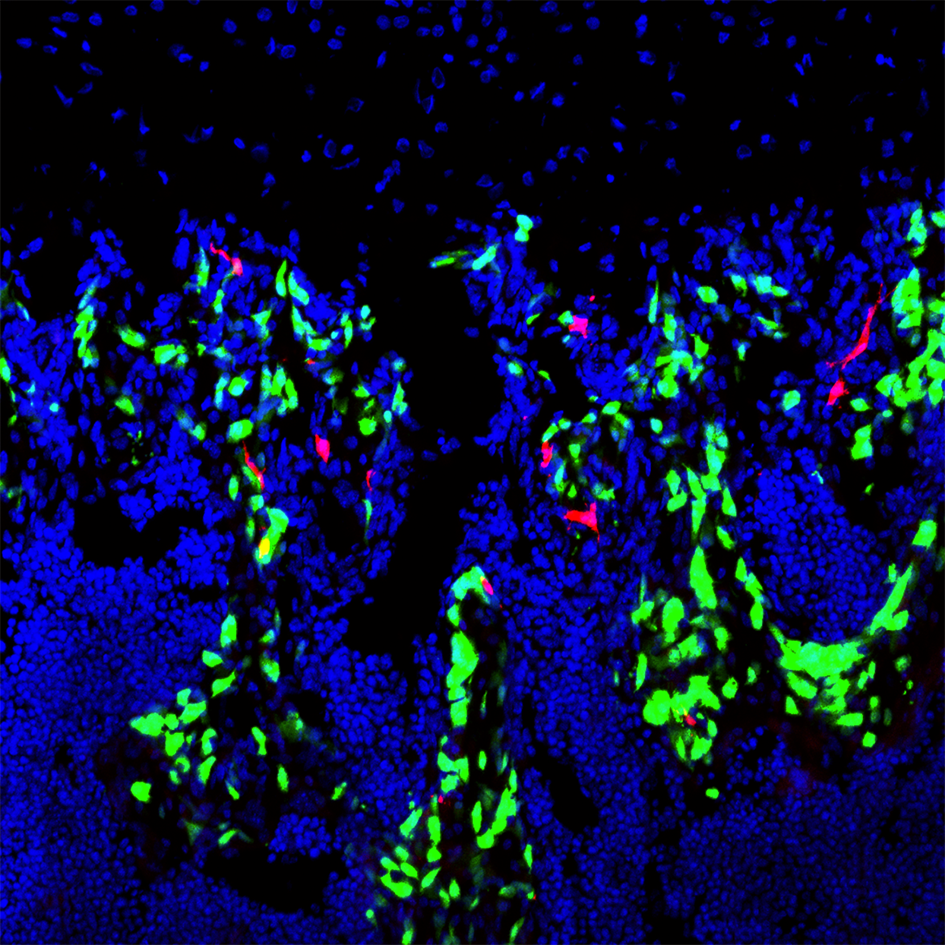

Supplement: Supplementary file 4 — Source data Fig. 2 [file 44318_2025_664_MOESM4_ESM.zip › Figure 2/2B/2B-i.tif]

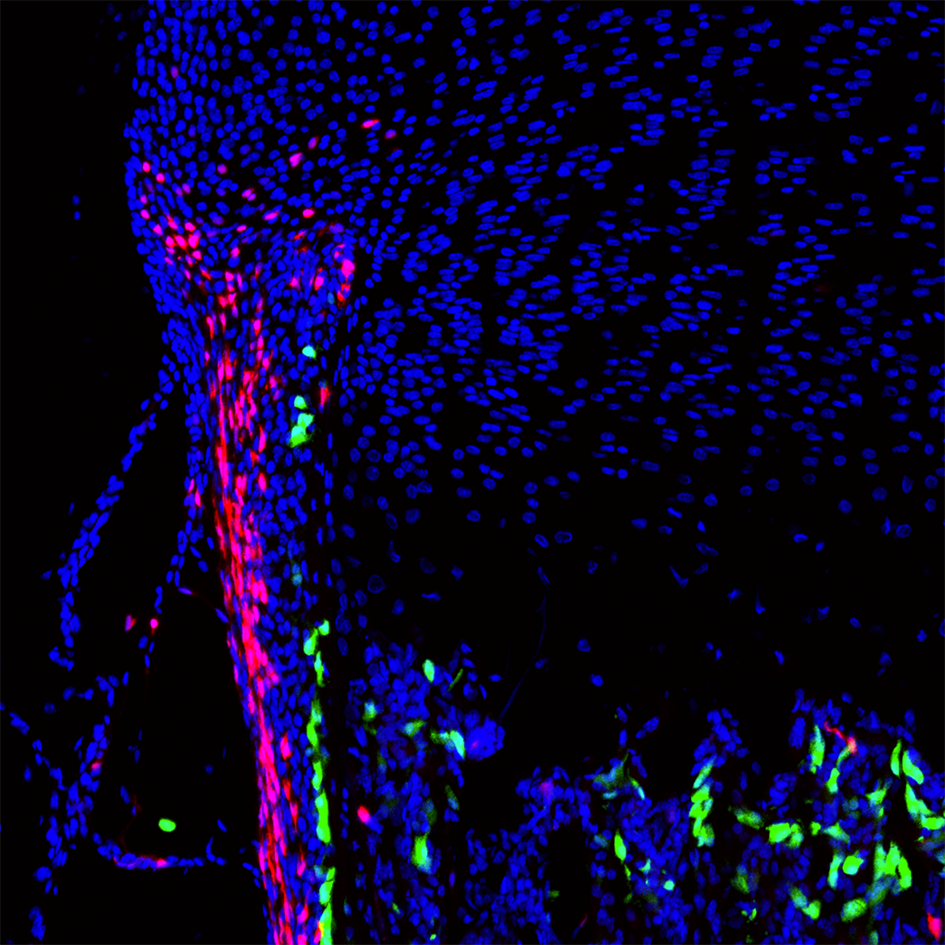

Supplement: Supplementary file 4 — Source data Fig. 2 [file 44318_2025_664_MOESM4_ESM.zip › Figure 2/2B/2B-ii.tif]

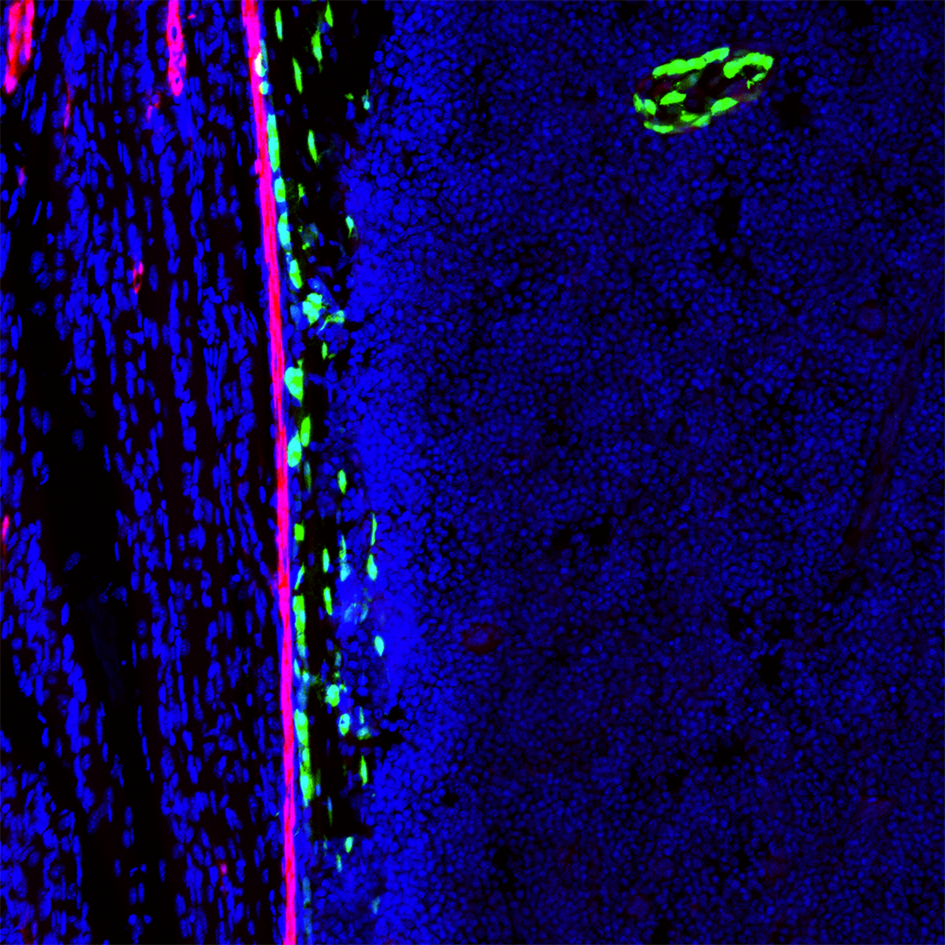

Supplement: Supplementary file 4 — Source data Fig. 2 [file 44318_2025_664_MOESM4_ESM.zip › Figure 2/2B/2B-iii.tif]

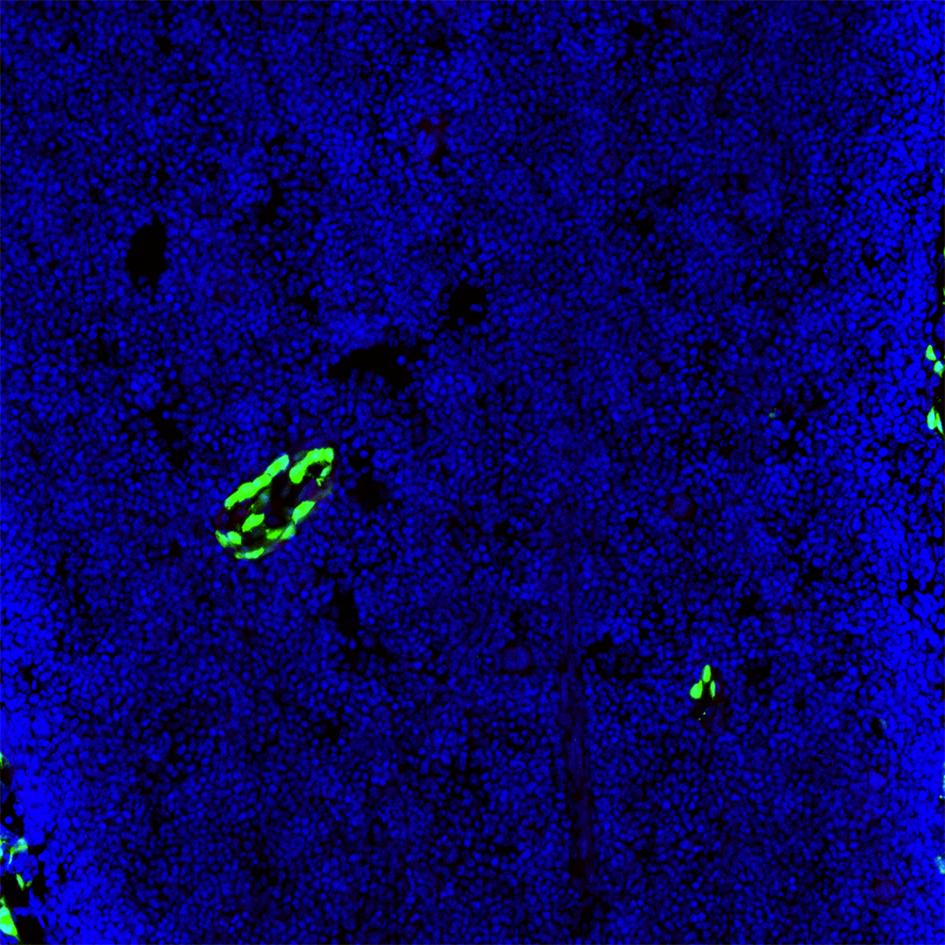

Supplement: Supplementary file 4 — Source data Fig. 2 [file 44318_2025_664_MOESM4_ESM.zip › Figure 2/2B/2B-iv.tif]

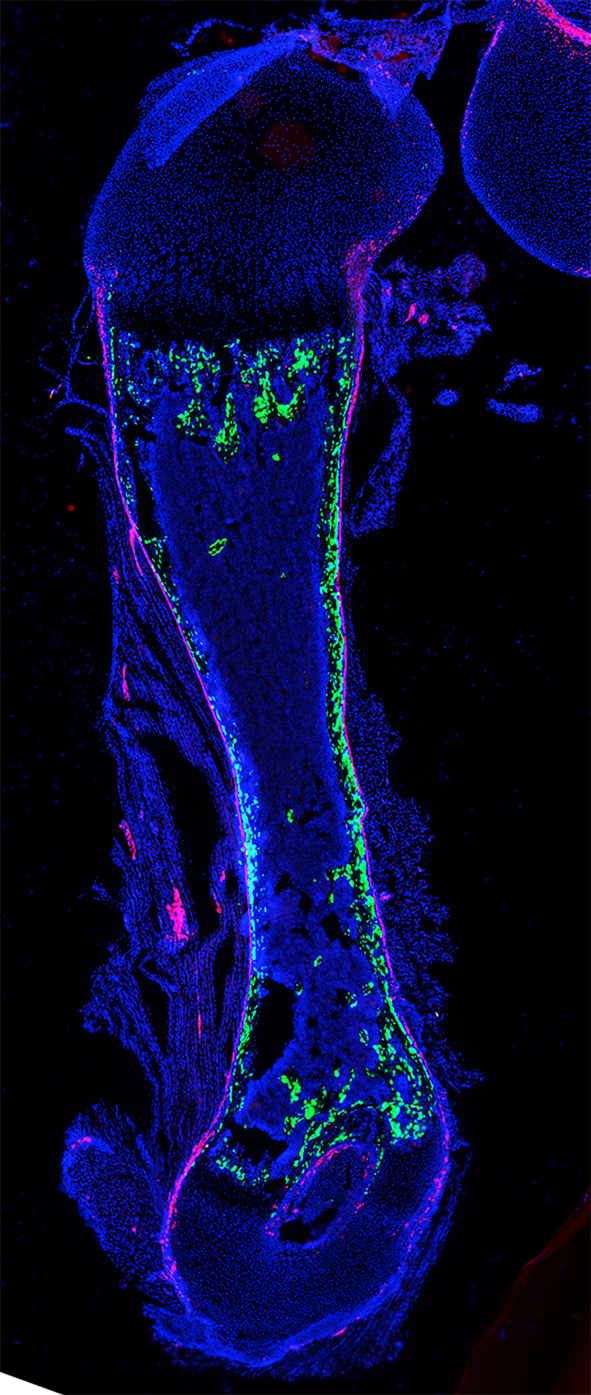

Supplement: Supplementary file 4 — Source data Fig. 2 [file 44318_2025_664_MOESM4_ESM.zip › Figure 2/2B/2B_left.tif]

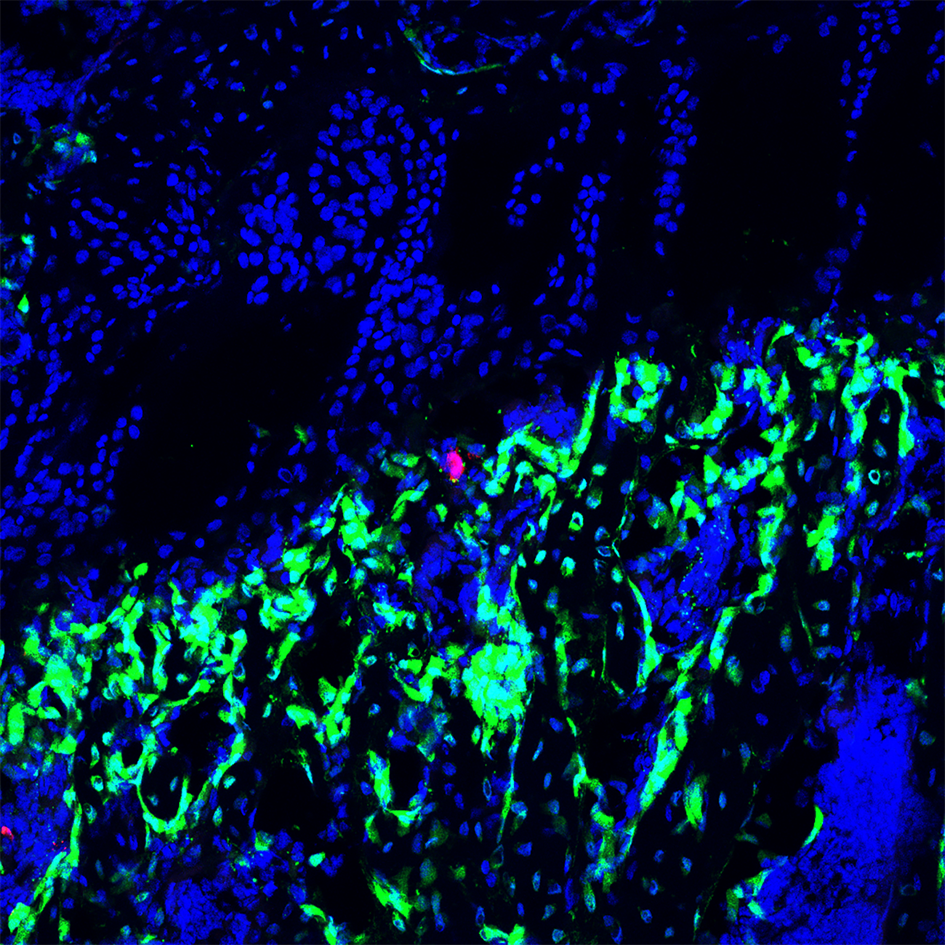

Supplement: Supplementary file 4 — Source data Fig. 2 [file 44318_2025_664_MOESM4_ESM.zip › Figure 2/2D/2D-i.tif]

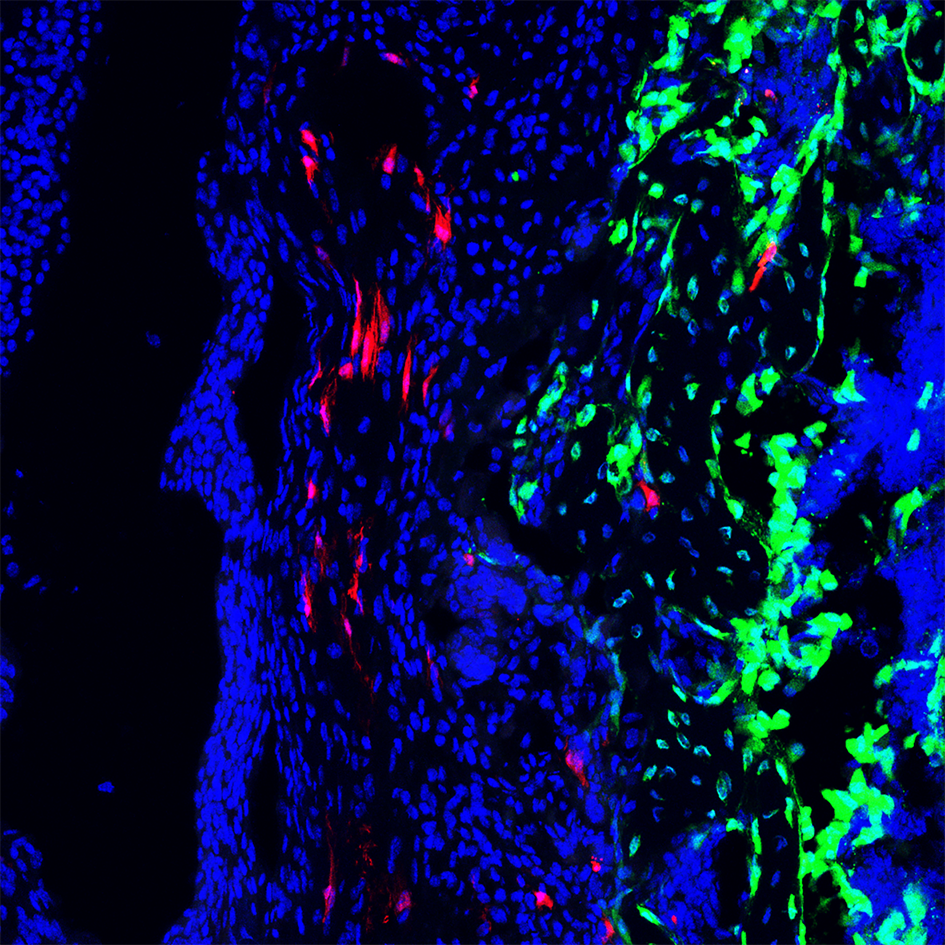

Supplement: Supplementary file 4 — Source data Fig. 2 [file 44318_2025_664_MOESM4_ESM.zip › Figure 2/2D/2D-ii.tif]

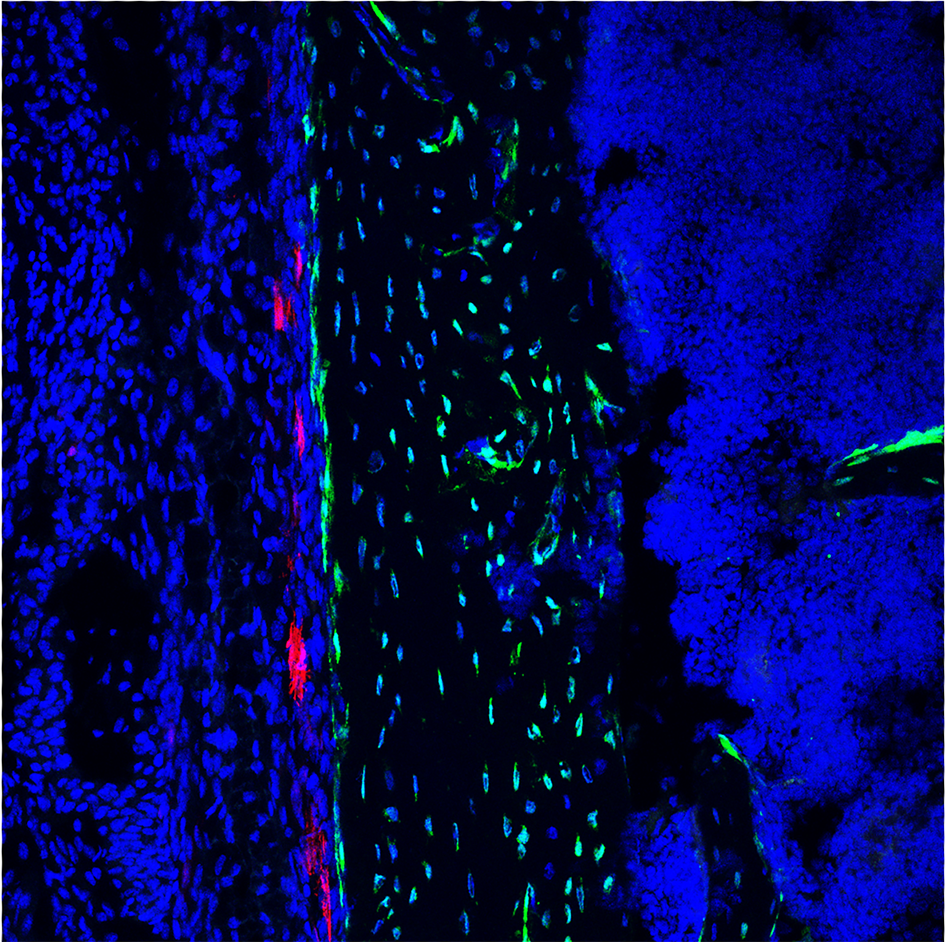

Supplement: Supplementary file 4 — Source data Fig. 2 [file 44318_2025_664_MOESM4_ESM.zip › Figure 2/2D/2D-iii.tif]

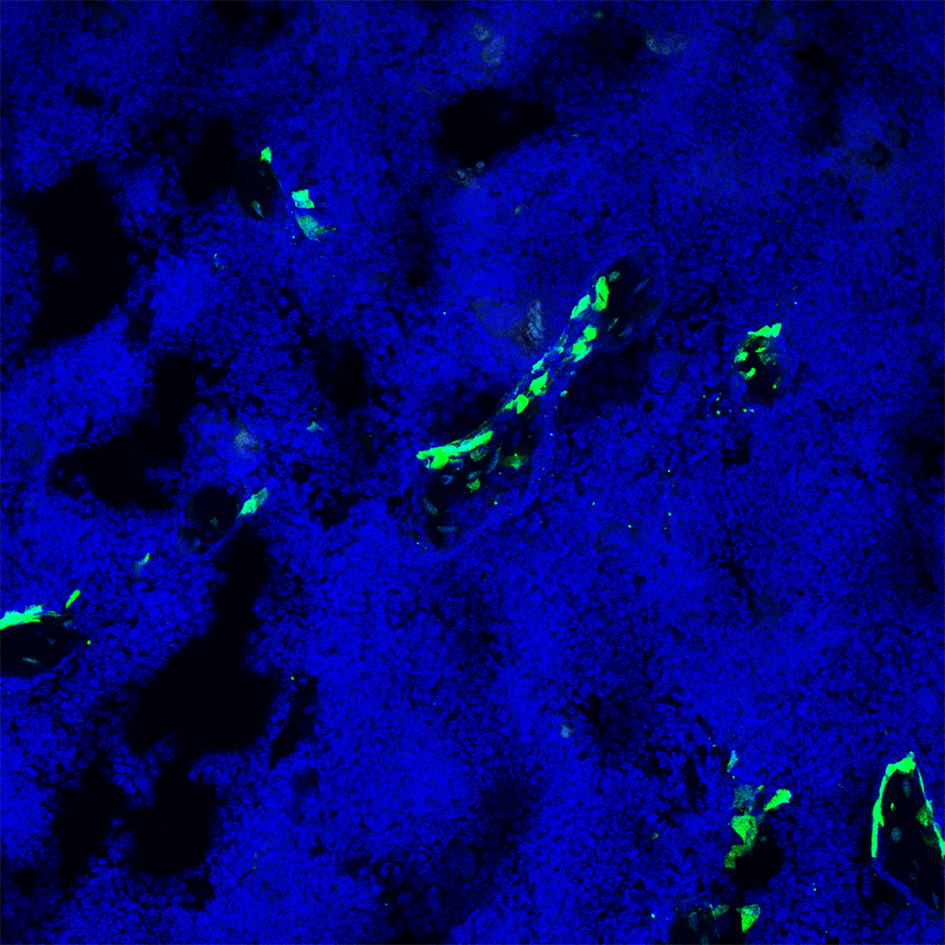

Supplement: Supplementary file 4 — Source data Fig. 2 [file 44318_2025_664_MOESM4_ESM.zip › Figure 2/2D/2D-iv.tif]

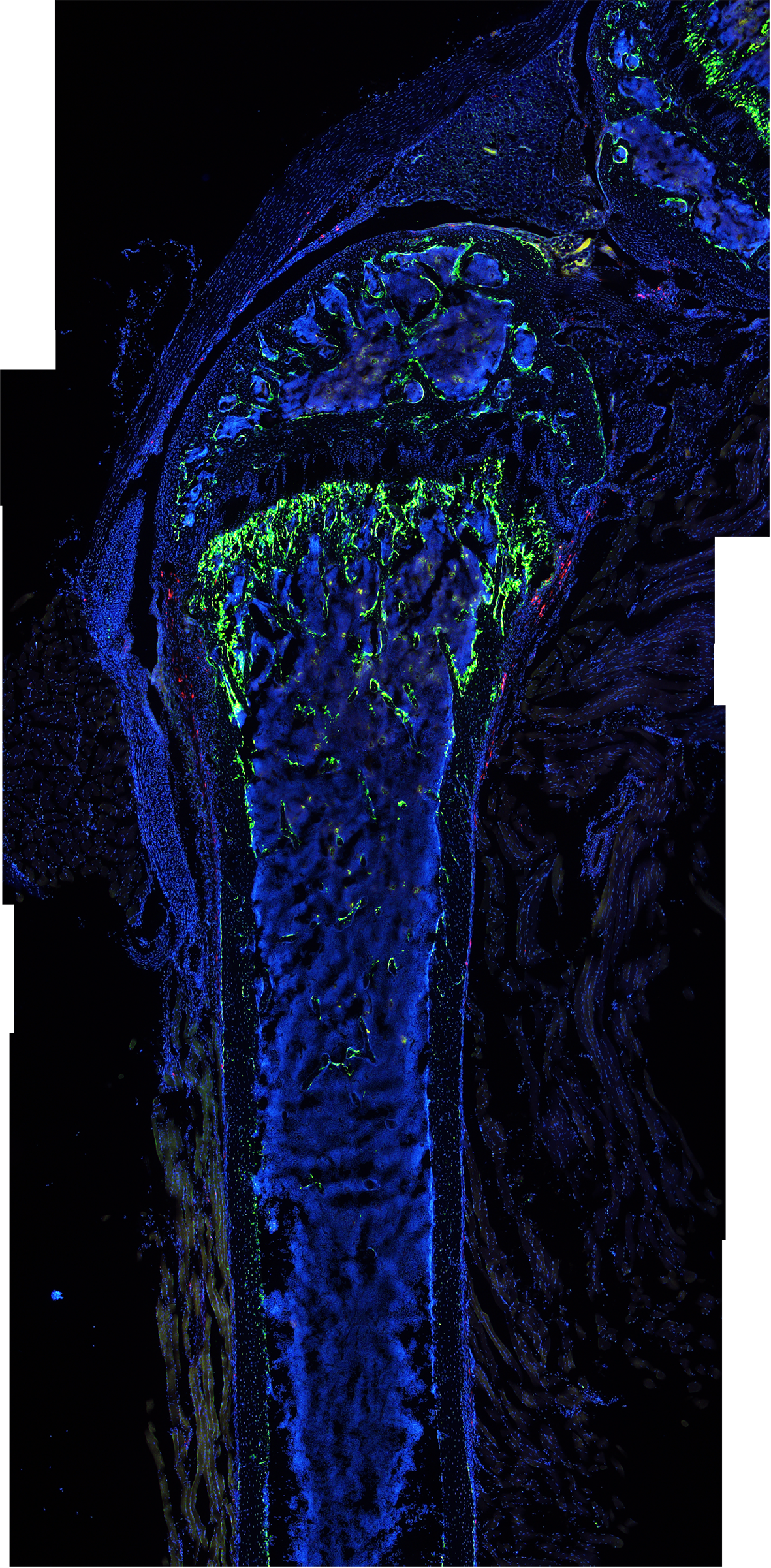

Supplement: Supplementary file 4 — Source data Fig. 2 [file 44318_2025_664_MOESM4_ESM.zip › Figure 2/2D/2D_left.tif]

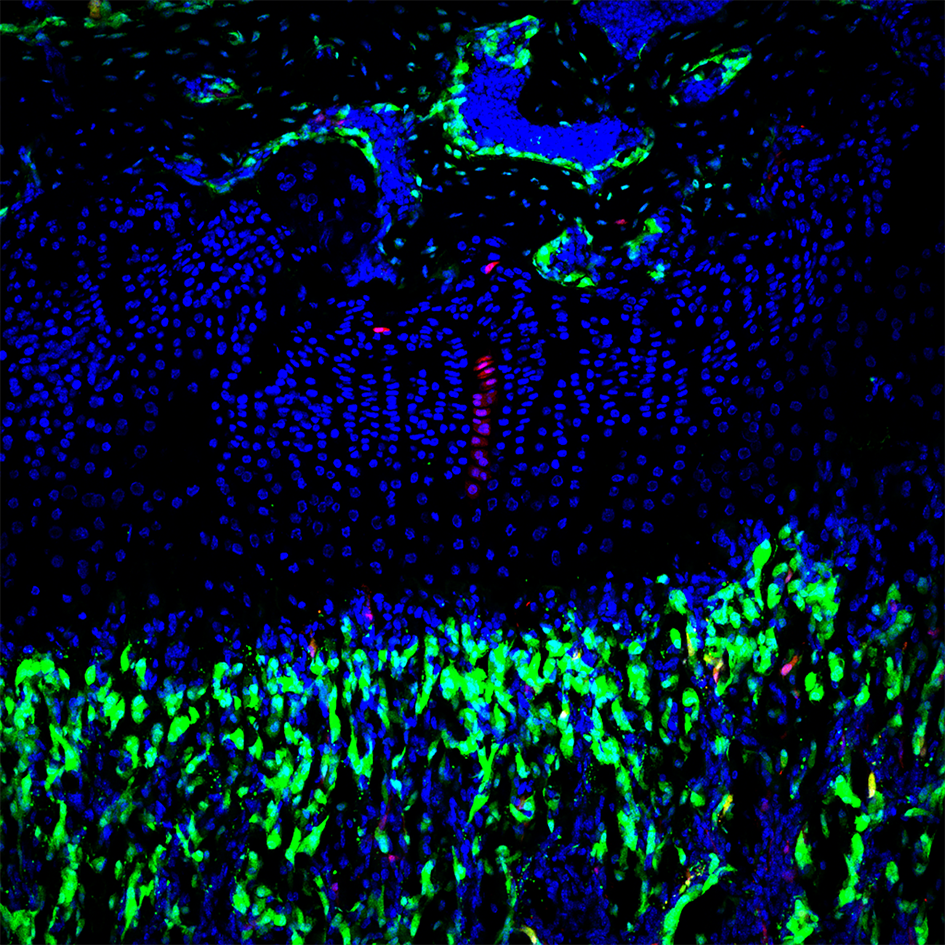

Supplement: Supplementary file 4 — Source data Fig. 2 [file 44318_2025_664_MOESM4_ESM.zip › Figure 2/2F/2F-i.tif]

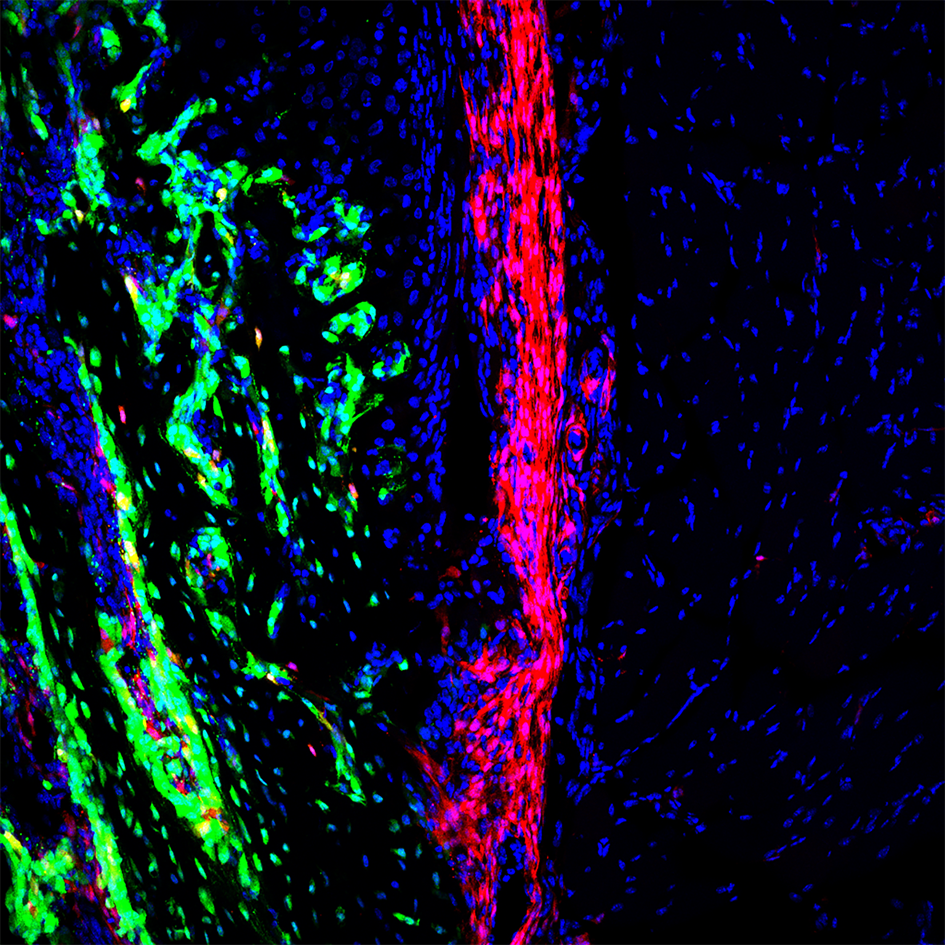

Supplement: Supplementary file 4 — Source data Fig. 2 [file 44318_2025_664_MOESM4_ESM.zip › Figure 2/2F/2F-ii.tif]

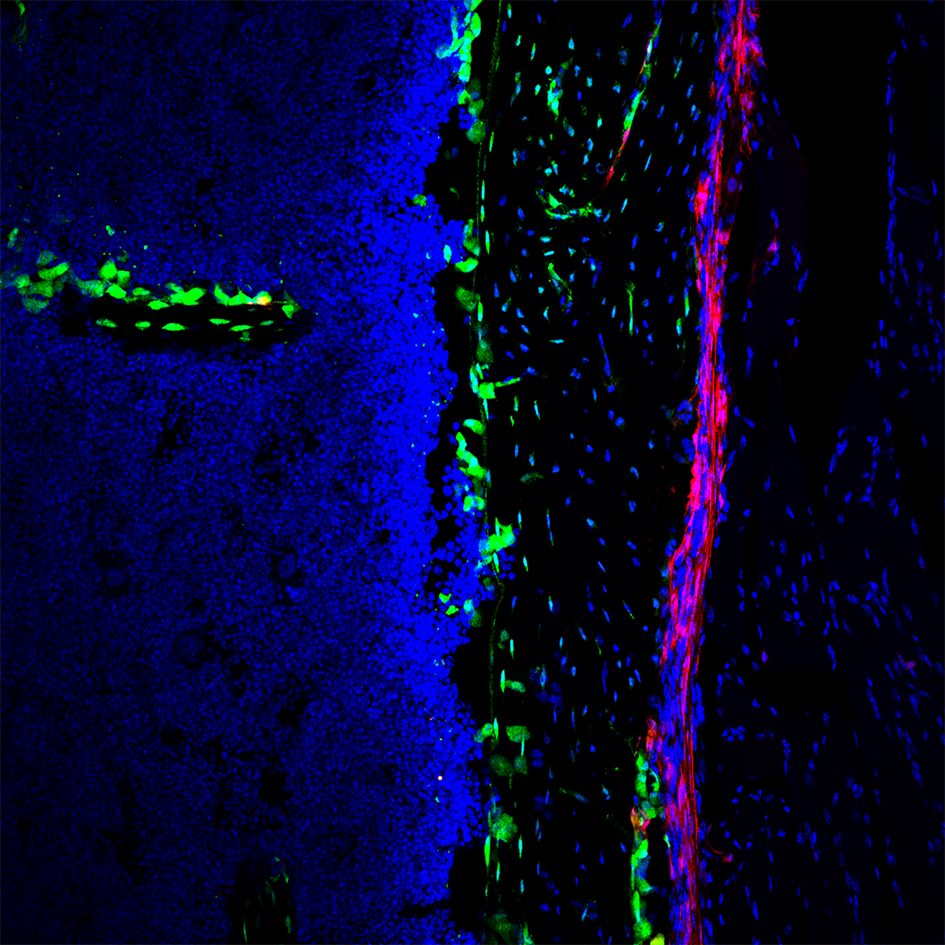

Supplement: Supplementary file 4 — Source data Fig. 2 [file 44318_2025_664_MOESM4_ESM.zip › Figure 2/2F/2F-iii.tif]

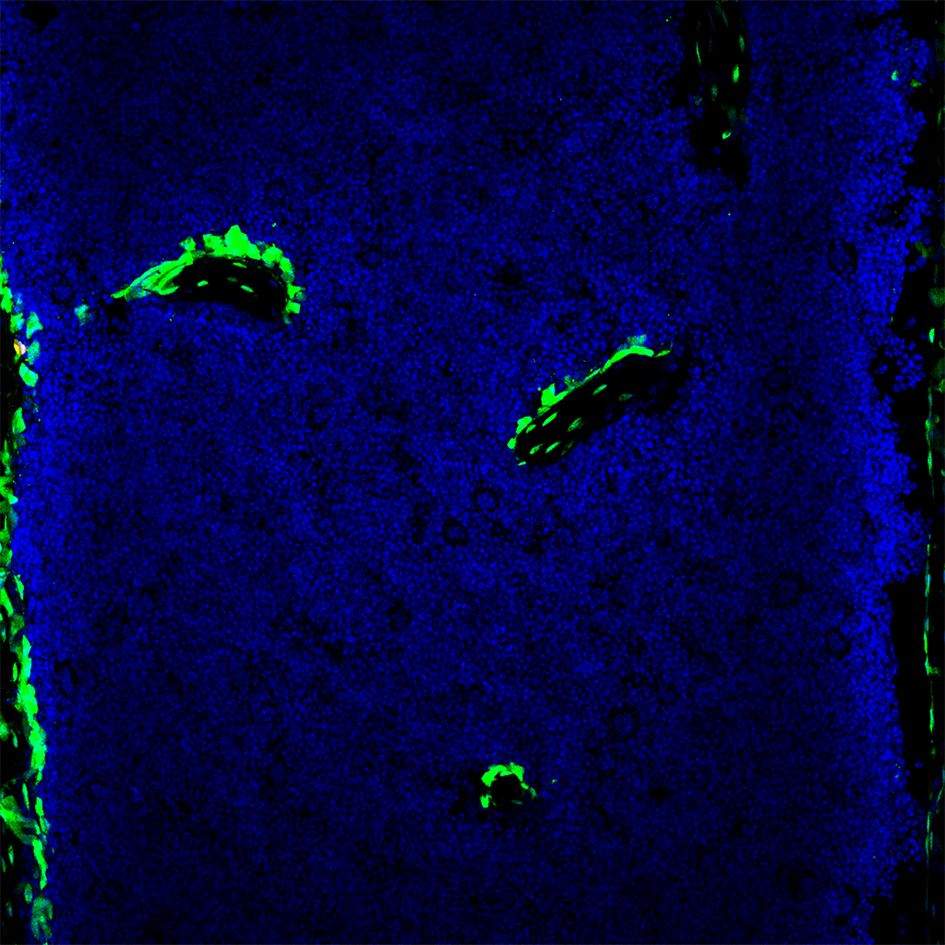

Supplement: Supplementary file 4 — Source data Fig. 2 [file 44318_2025_664_MOESM4_ESM.zip › Figure 2/2F/2F-iv.tif]

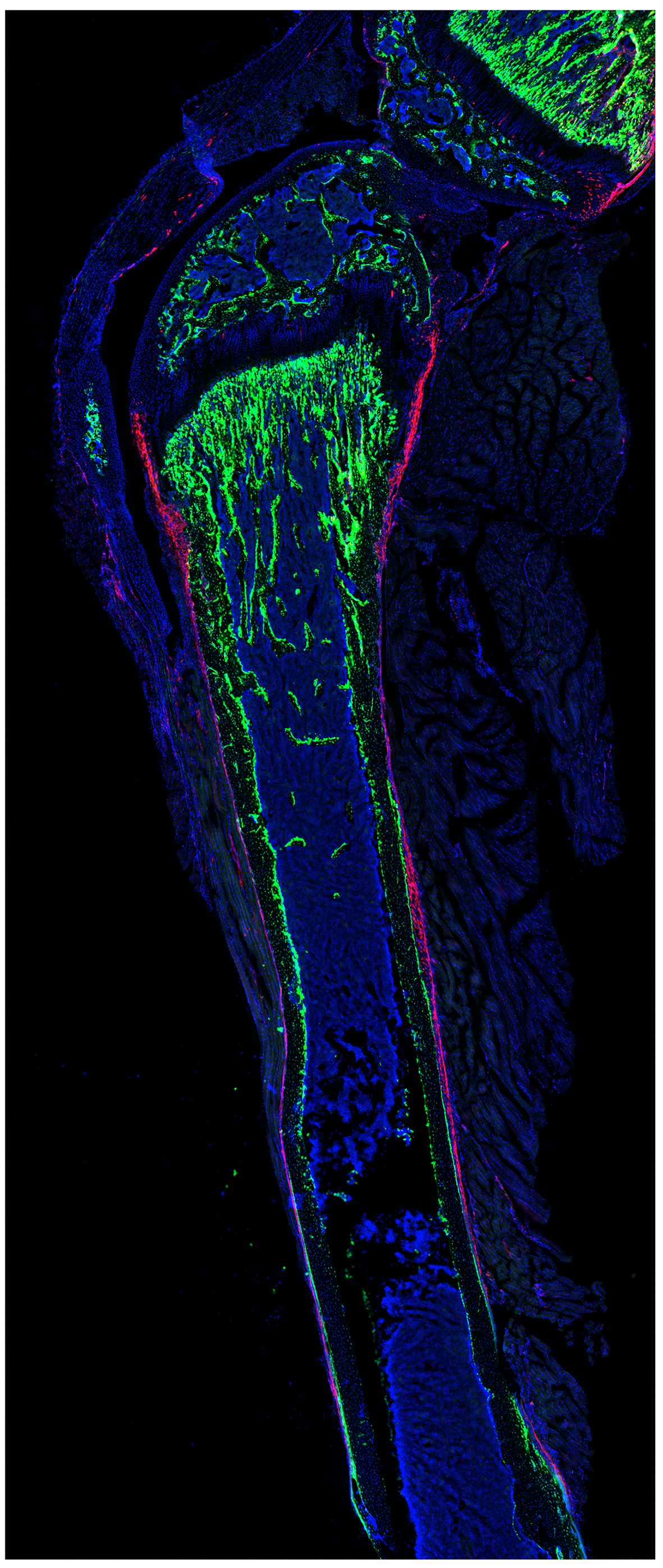

Supplement: Supplementary file 4 — Source data Fig. 2 [file 44318_2025_664_MOESM4_ESM.zip › Figure 2/2F/2F_left.tif]

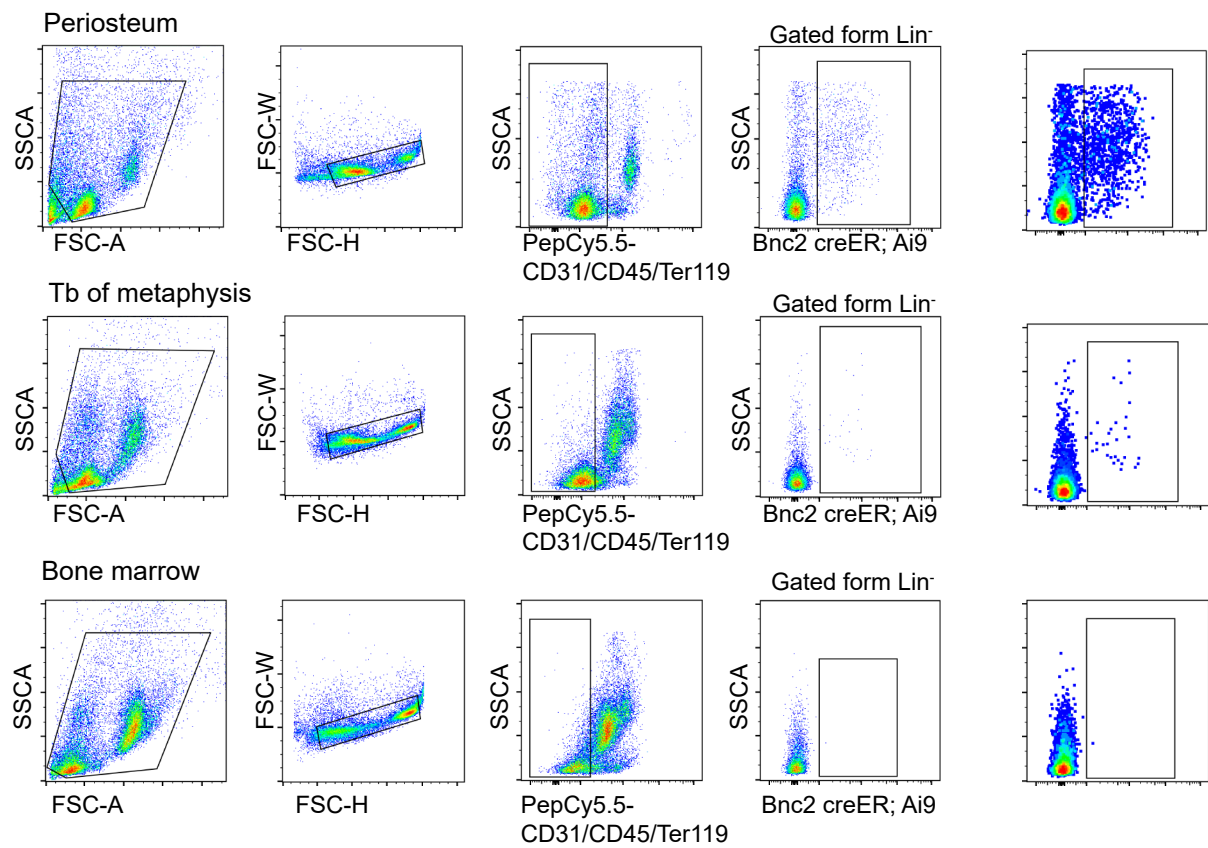

Supplement: Supplementary file 4 — Source data Fig. 2 [file 44318_2025_664_MOESM4_ESM.zip › Figure 2/2H/Flow cytometry analysis of the proportion of Bnc2-creER+ cells in long bone.pdf]

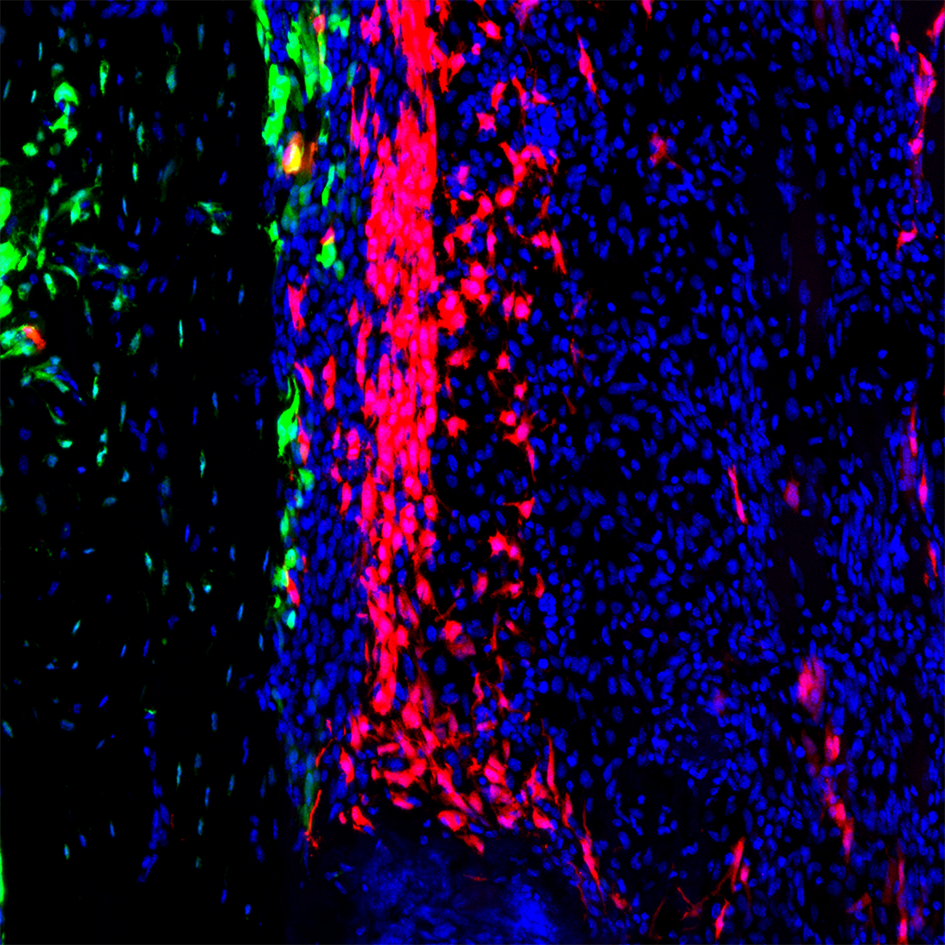

Supplement: Supplementary file 5 — Source data Fig. 3 [file 44318_2025_664_MOESM5_ESM.zip › Figure 3/3B/3B-i.tif]

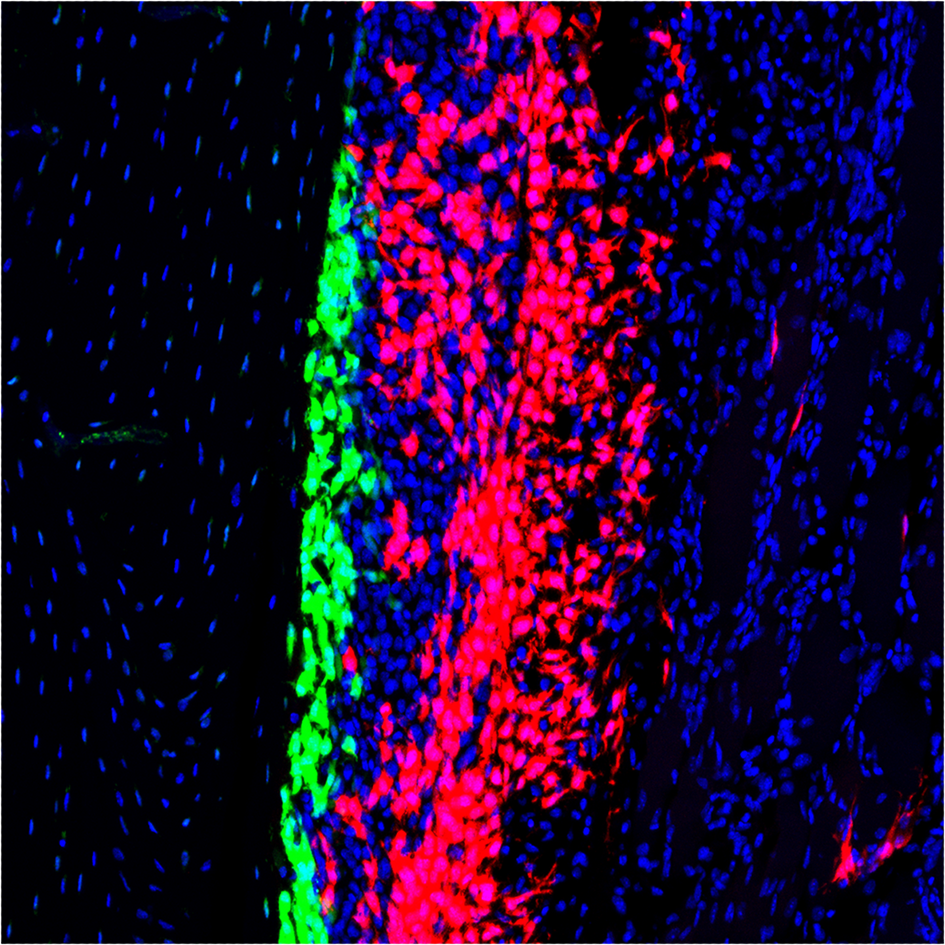

Supplement: Supplementary file 5 — Source data Fig. 3 [file 44318_2025_664_MOESM5_ESM.zip › Figure 3/3B/3B-ii.tif]

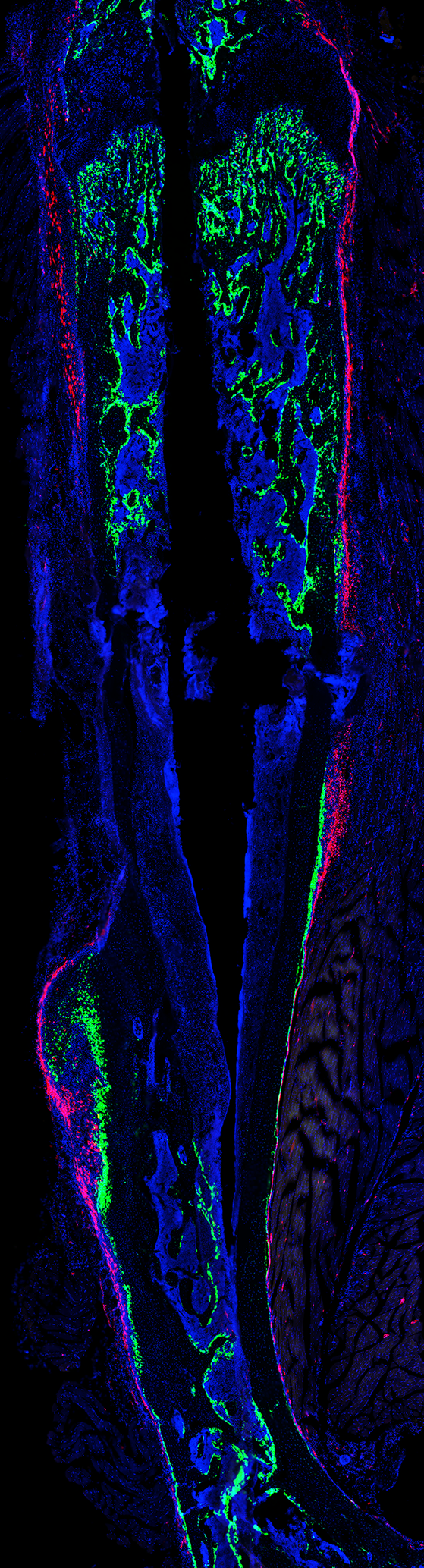

Supplement: Supplementary file 5 — Source data Fig. 3 [file 44318_2025_664_MOESM5_ESM.zip › Figure 3/3B/3B_left.tif]

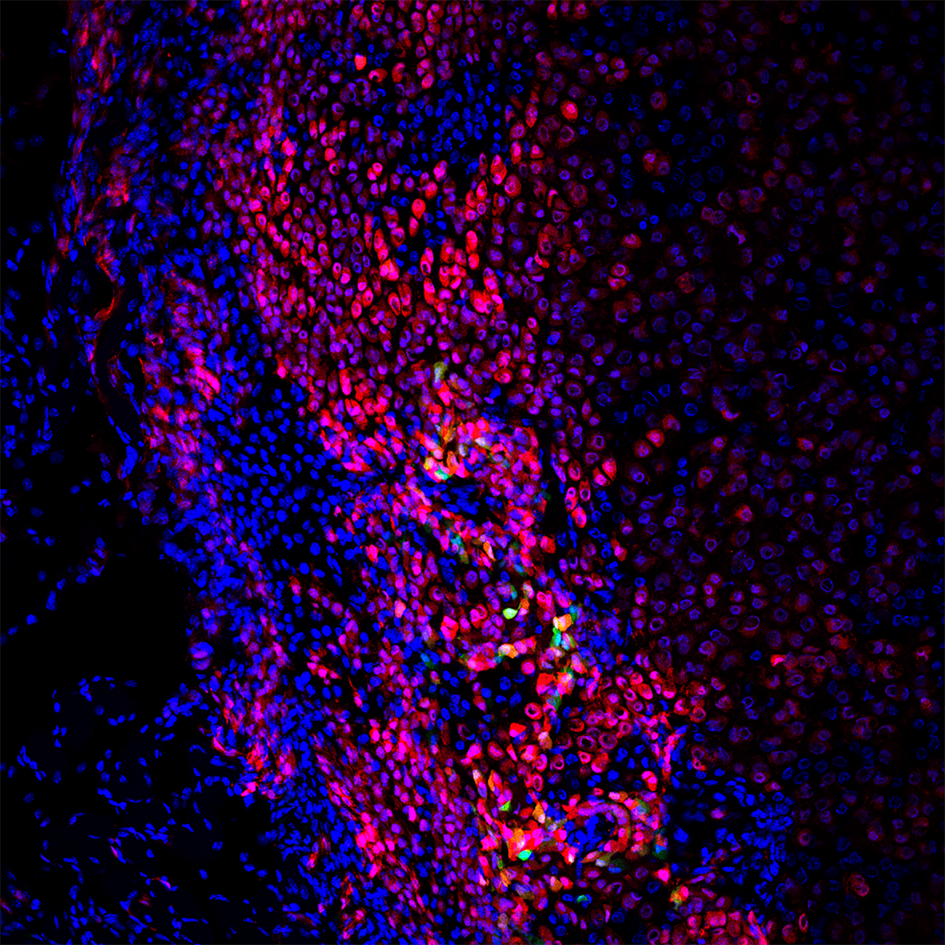

Supplement: Supplementary file 5 — Source data Fig. 3 [file 44318_2025_664_MOESM5_ESM.zip › Figure 3/3C/3C-i.tif]

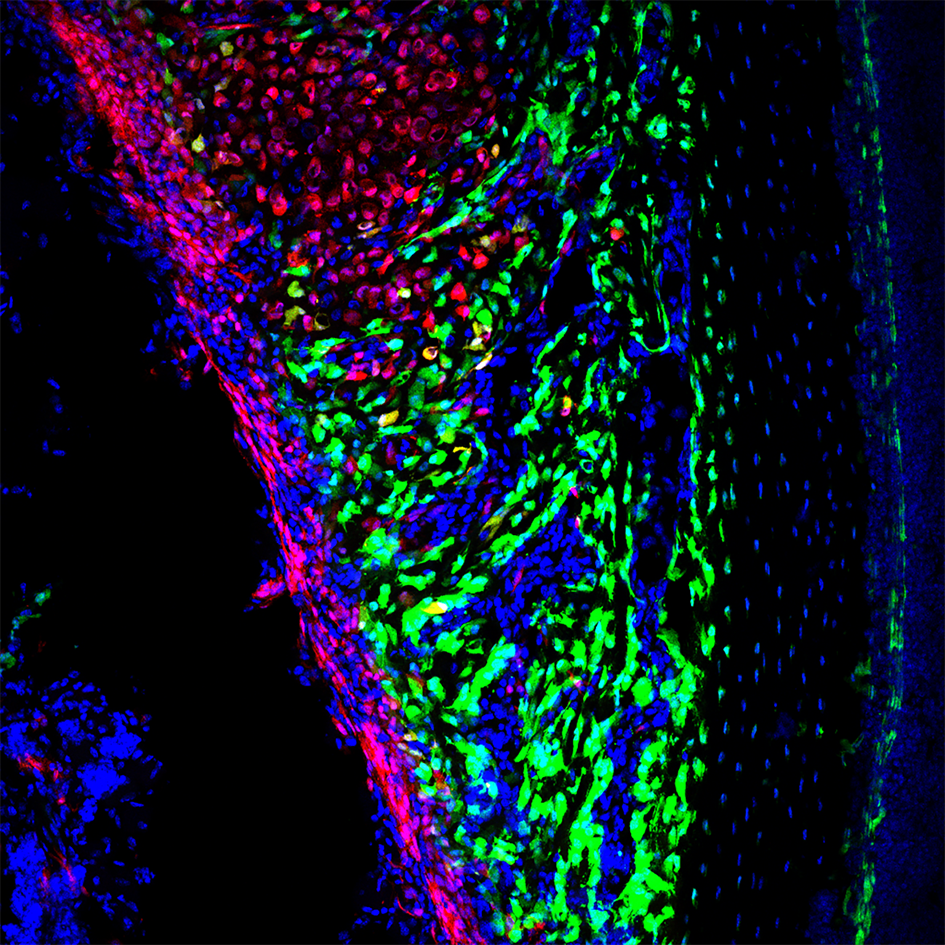

Supplement: Supplementary file 5 — Source data Fig. 3 [file 44318_2025_664_MOESM5_ESM.zip › Figure 3/3C/3C-ii.tif]

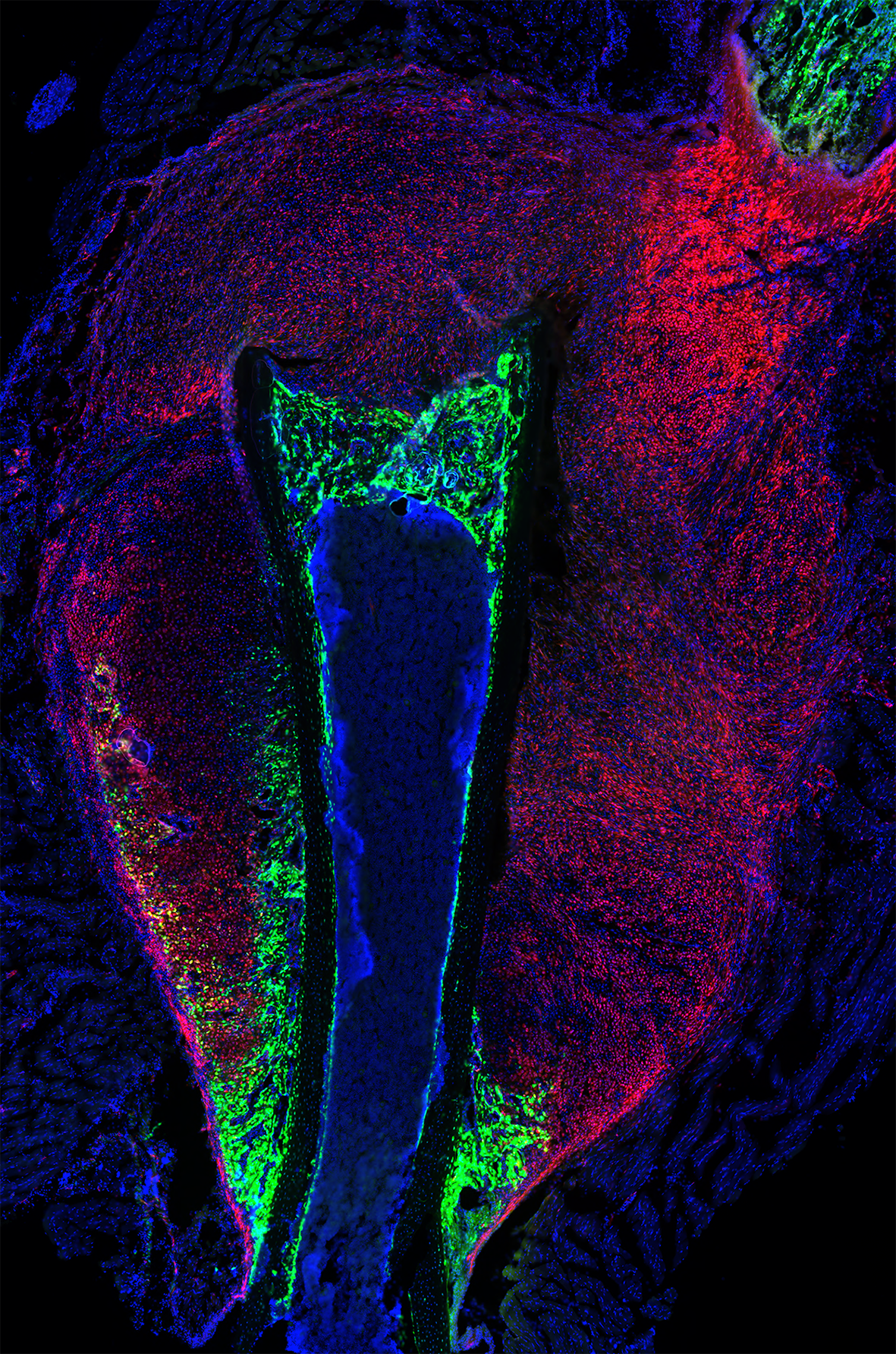

Supplement: Supplementary file 5 — Source data Fig. 3 [file 44318_2025_664_MOESM5_ESM.zip › Figure 3/3C/3C_left.tif]

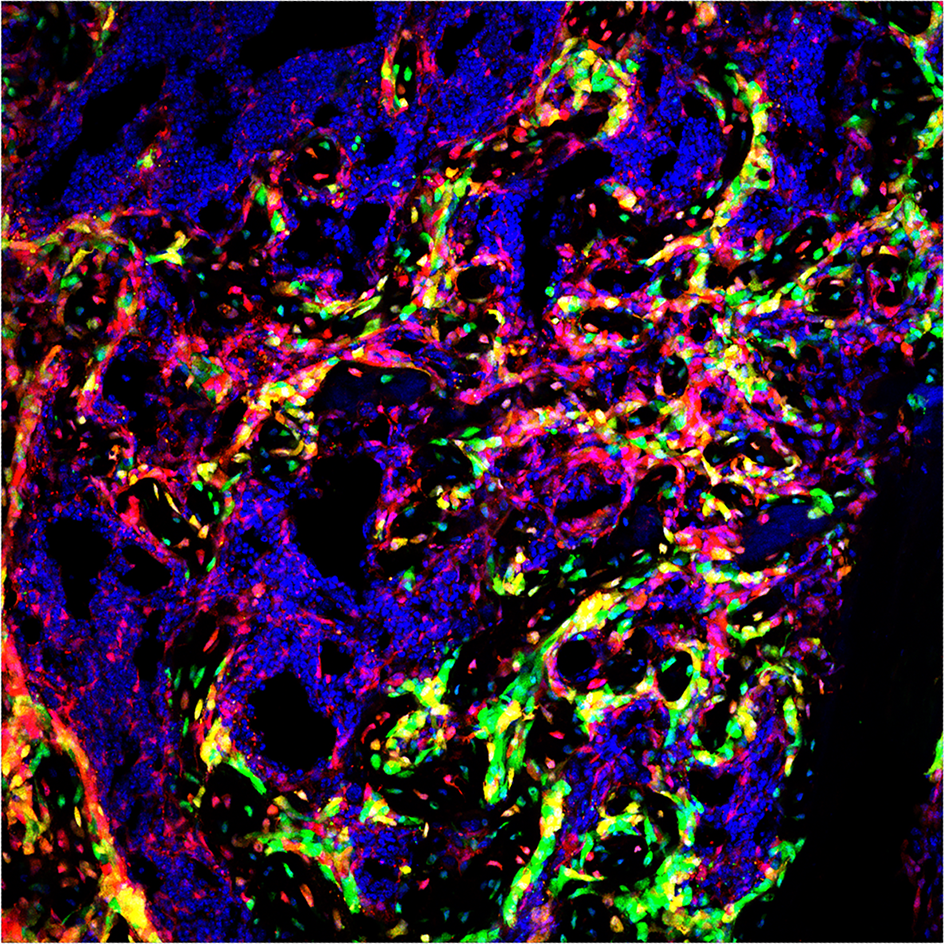

Supplement: Supplementary file 5 — Source data Fig. 3 [file 44318_2025_664_MOESM5_ESM.zip › Figure 3/3D/3D-i.tif]

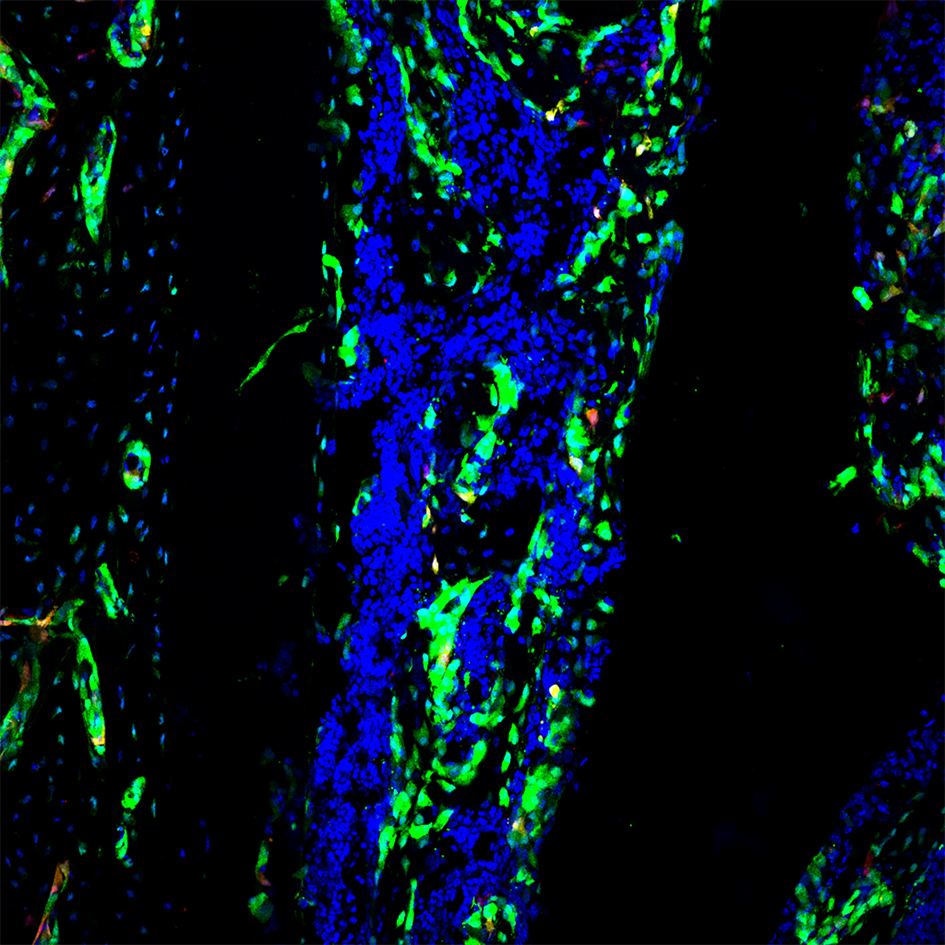

Supplement: Supplementary file 5 — Source data Fig. 3 [file 44318_2025_664_MOESM5_ESM.zip › Figure 3/3D/3D-ii.tif]

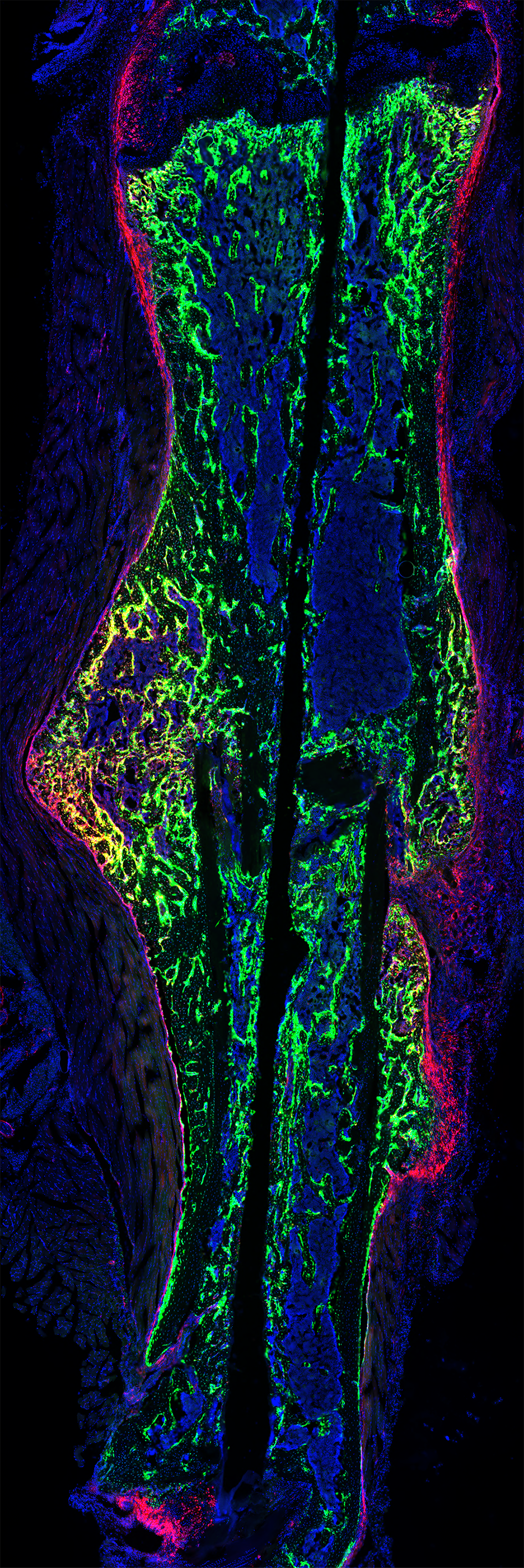

Supplement: Supplementary file 5 — Source data Fig. 3 [file 44318_2025_664_MOESM5_ESM.zip › Figure 3/3D/3D_left.tif]

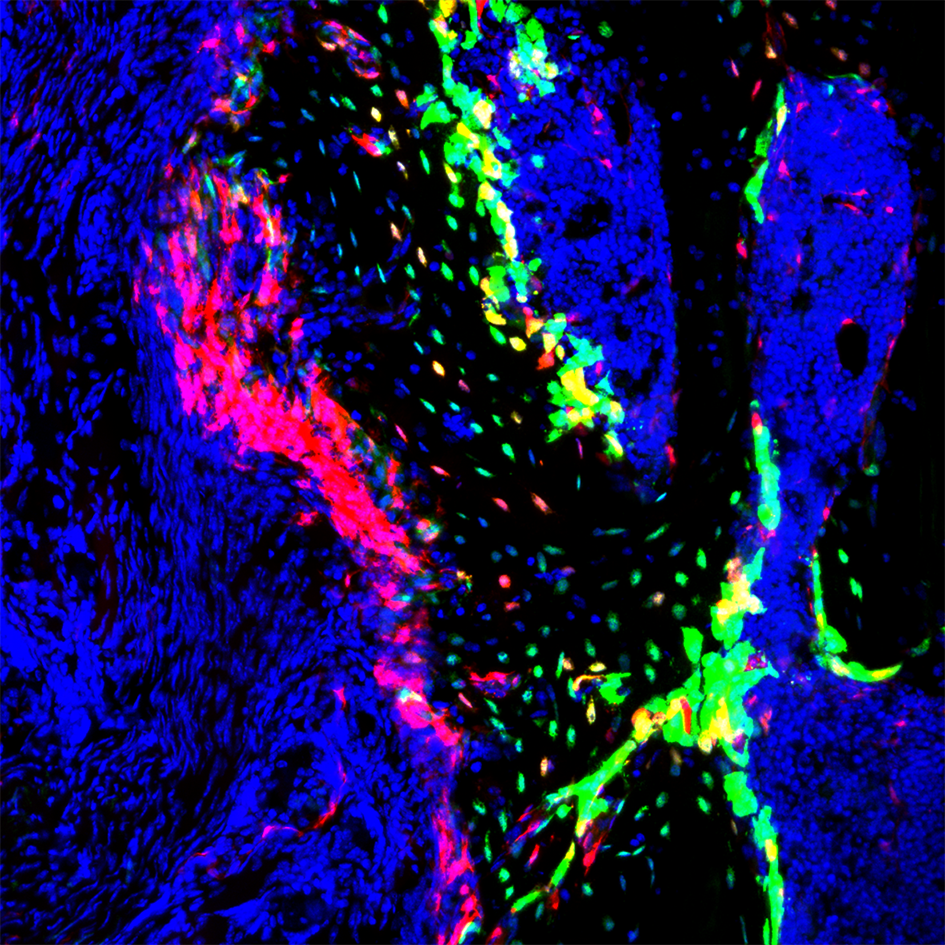

Supplement: Supplementary file 5 — Source data Fig. 3 [file 44318_2025_664_MOESM5_ESM.zip › Figure 3/3E/3E-i.tif]

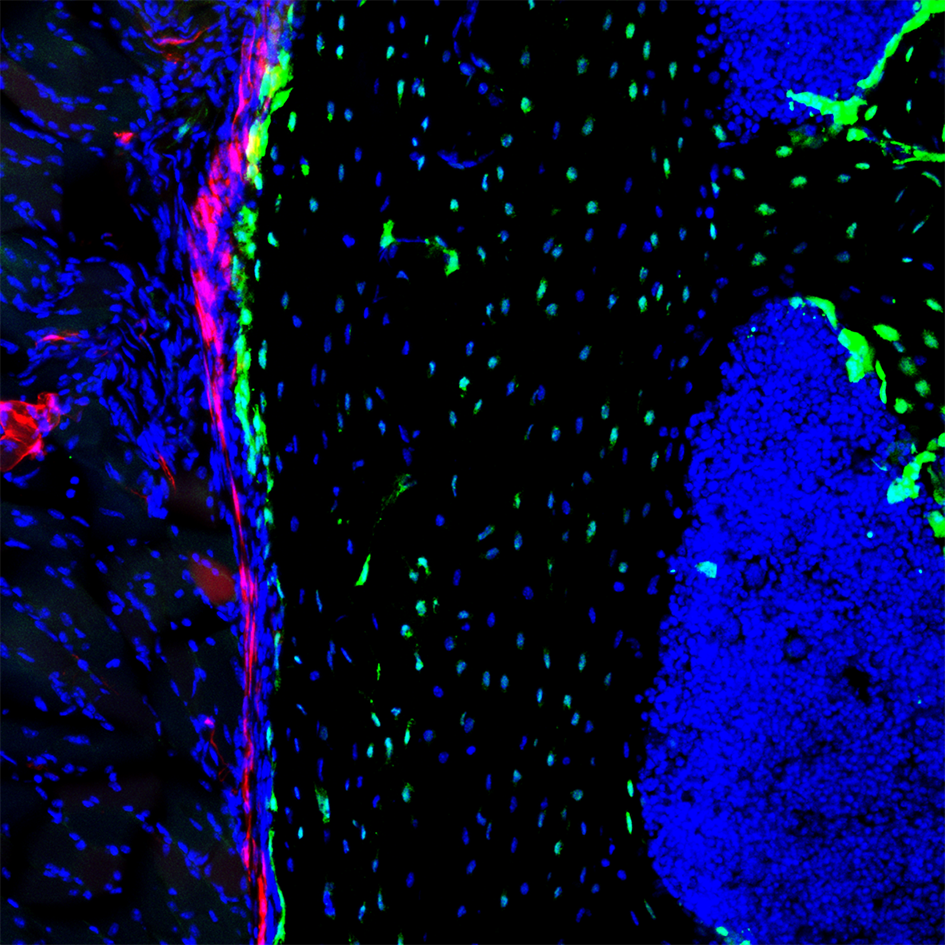

Supplement: Supplementary file 5 — Source data Fig. 3 [file 44318_2025_664_MOESM5_ESM.zip › Figure 3/3E/3E-ii.tif]

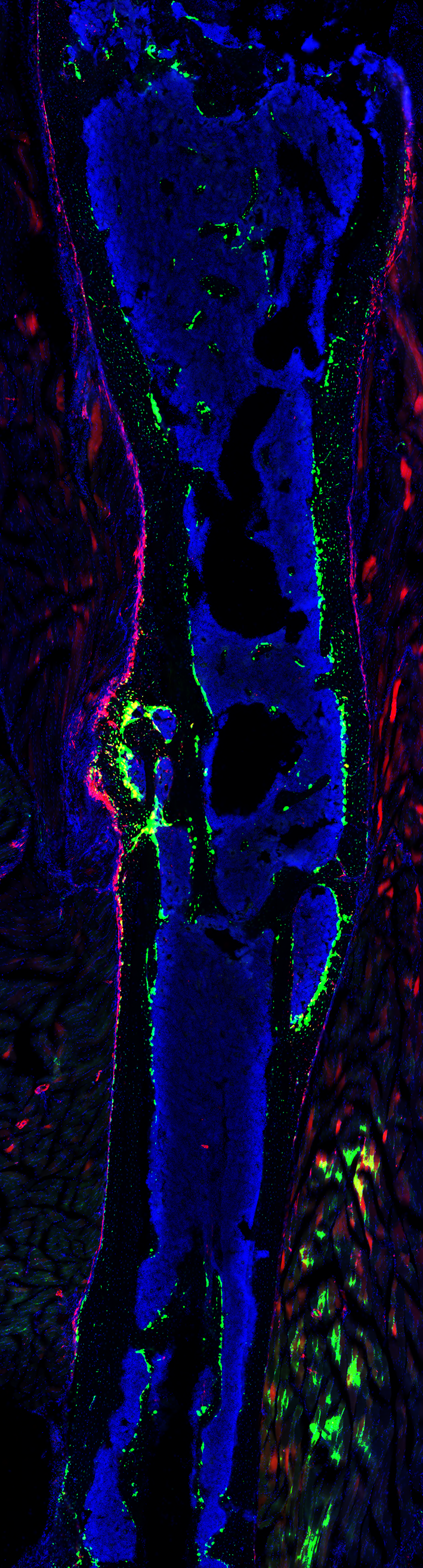

Supplement: Supplementary file 5 — Source data Fig. 3 [file 44318_2025_664_MOESM5_ESM.zip › Figure 3/3E/3E_left.tif]

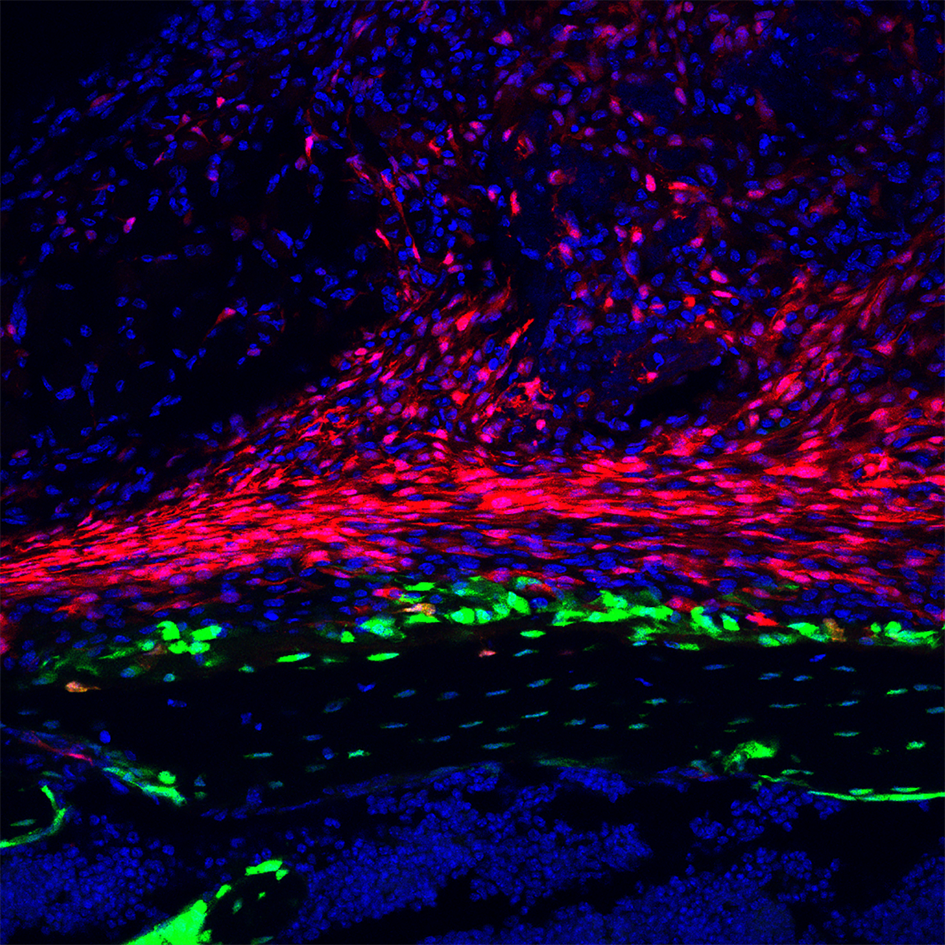

Supplement: Supplementary file 5 — Source data Fig. 3 [file 44318_2025_664_MOESM5_ESM.zip › Figure 3/3G/3G-i.tif]

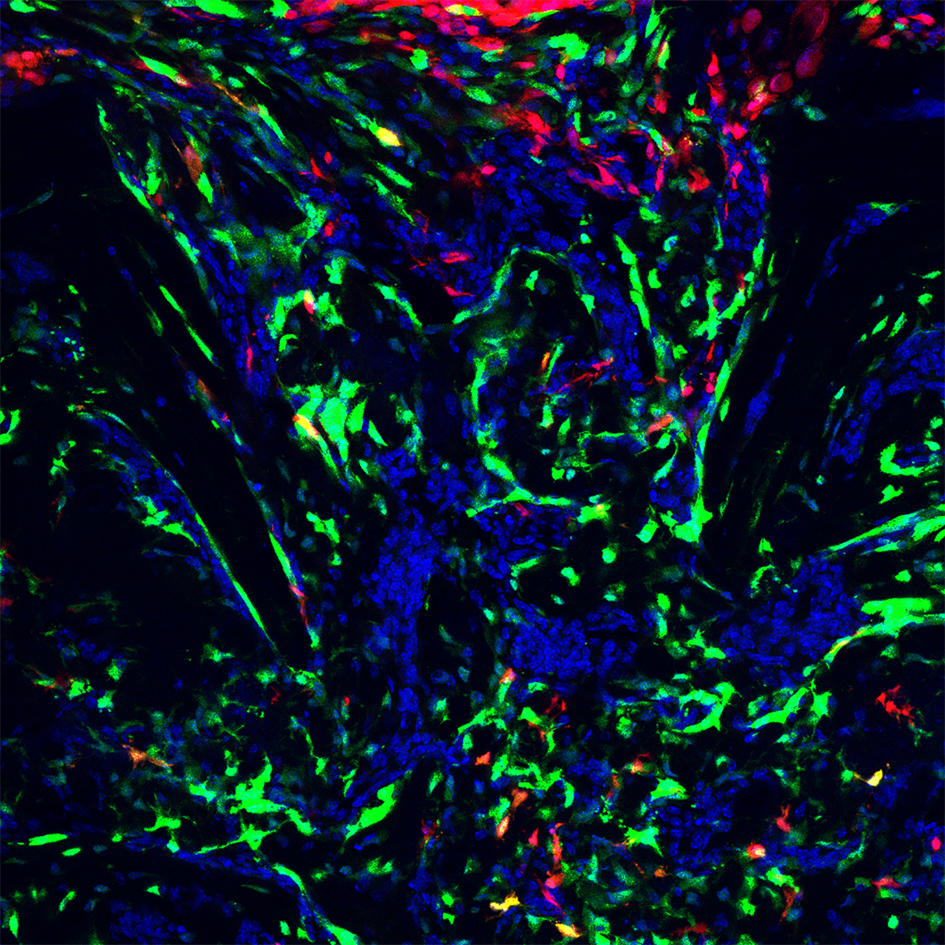

Supplement: Supplementary file 5 — Source data Fig. 3 [file 44318_2025_664_MOESM5_ESM.zip › Figure 3/3G/3G-ii.tif]

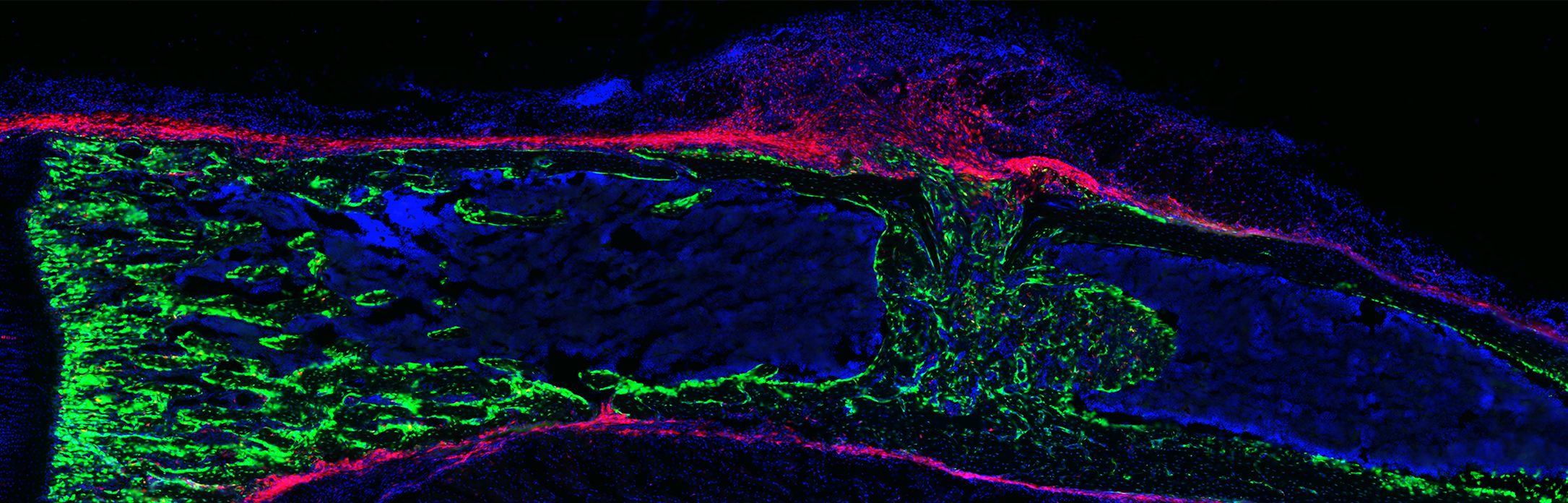

Supplement: Supplementary file 5 — Source data Fig. 3 [file 44318_2025_664_MOESM5_ESM.zip › Figure 3/3G/3G_left.tif]

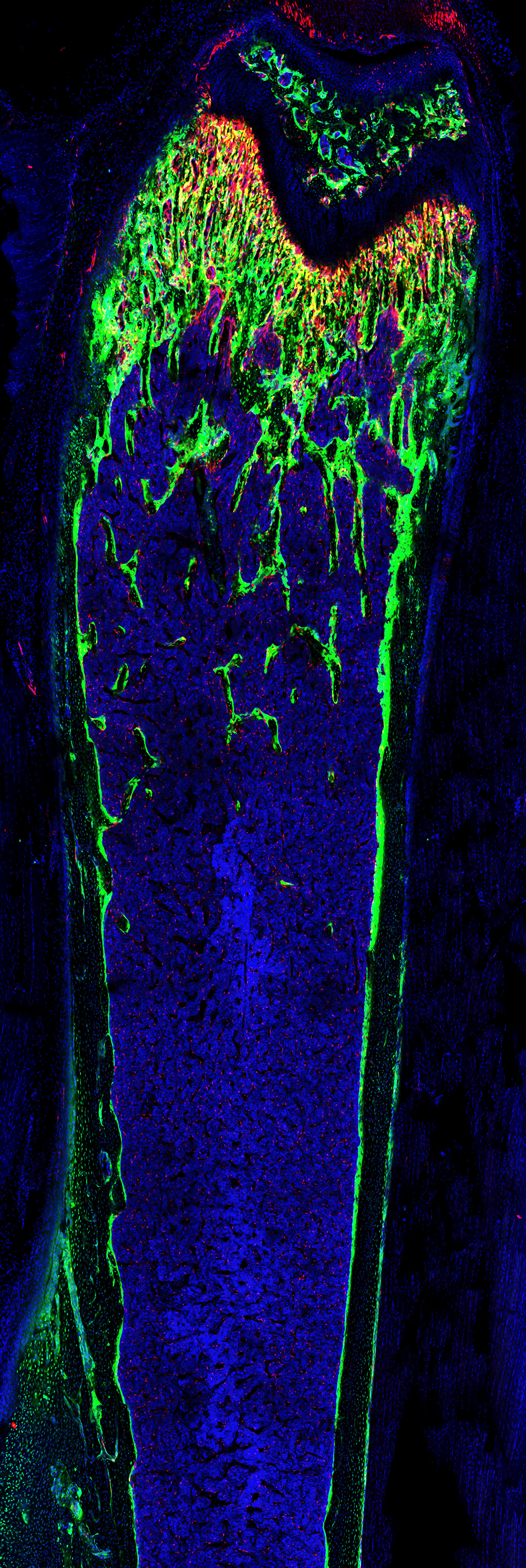

Supplement: Supplementary file 6 — Source data Fig. 4 [file 44318_2025_664_MOESM6_ESM.zip › Figure 4/4B/4B.tif]

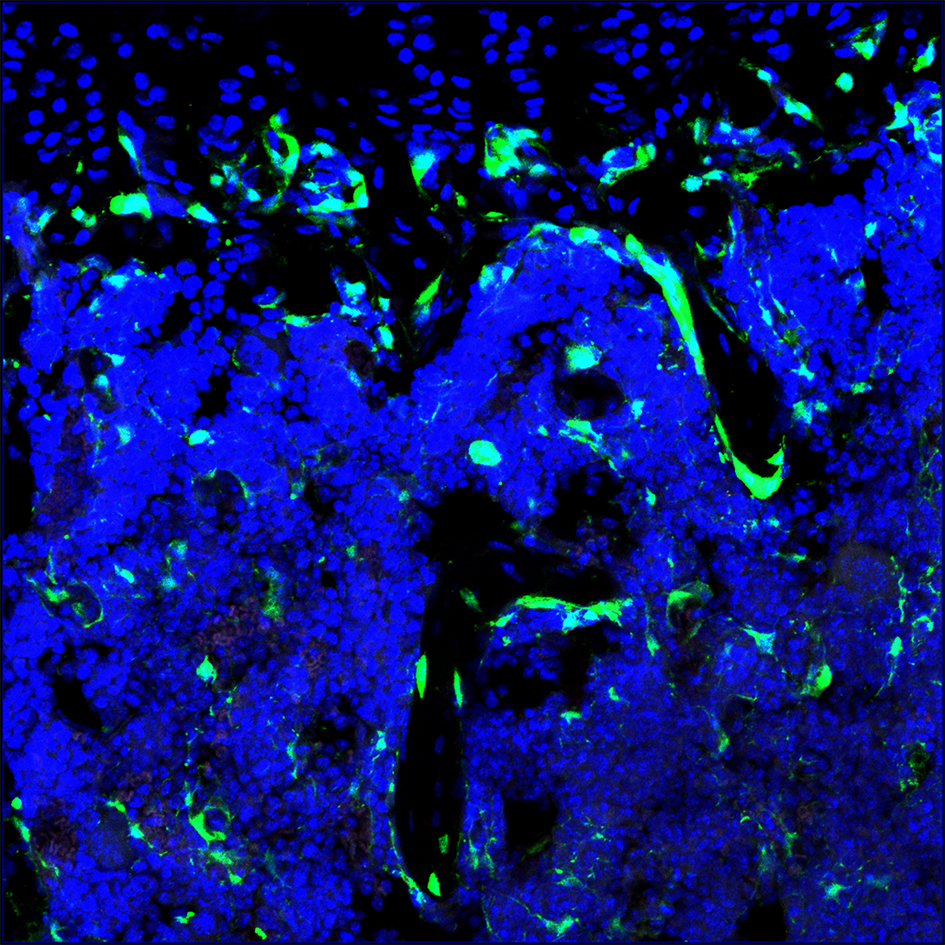

Supplement: Supplementary file 6 — Source data Fig. 4 [file 44318_2025_664_MOESM6_ESM.zip › Figure 4/4D/4D-i.tif]

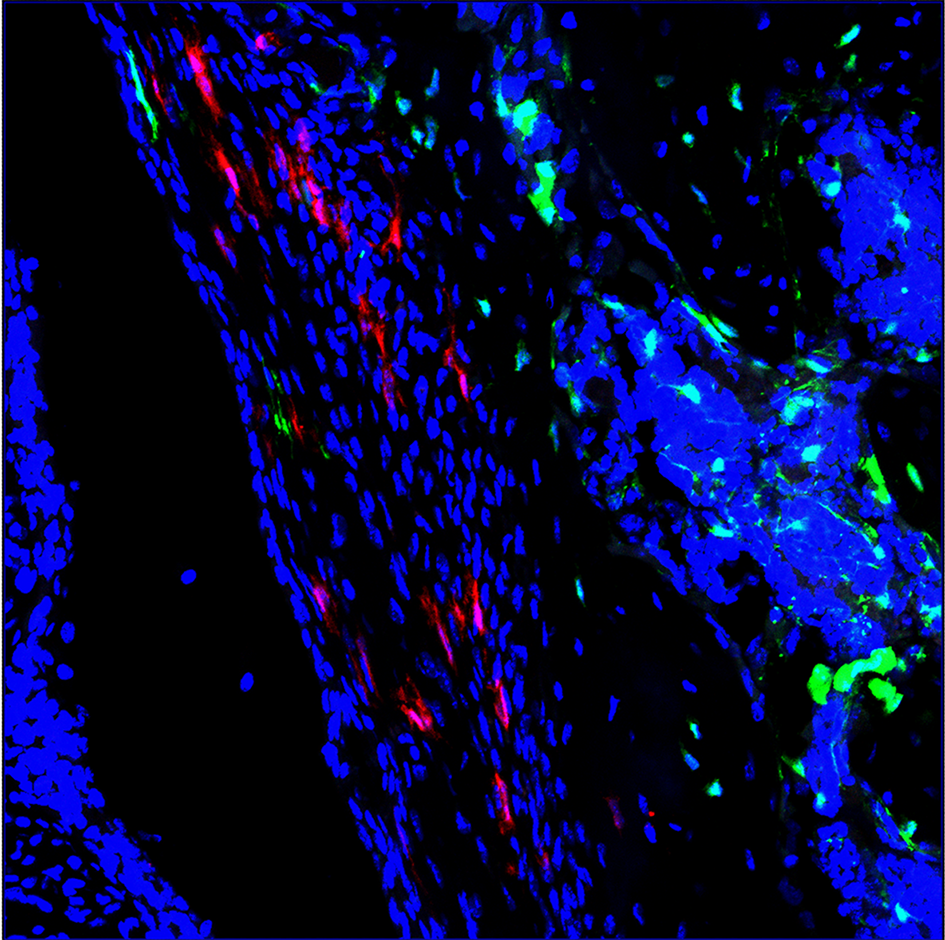

Supplement: Supplementary file 6 — Source data Fig. 4 [file 44318_2025_664_MOESM6_ESM.zip › Figure 4/4D/4D-ii.tif]

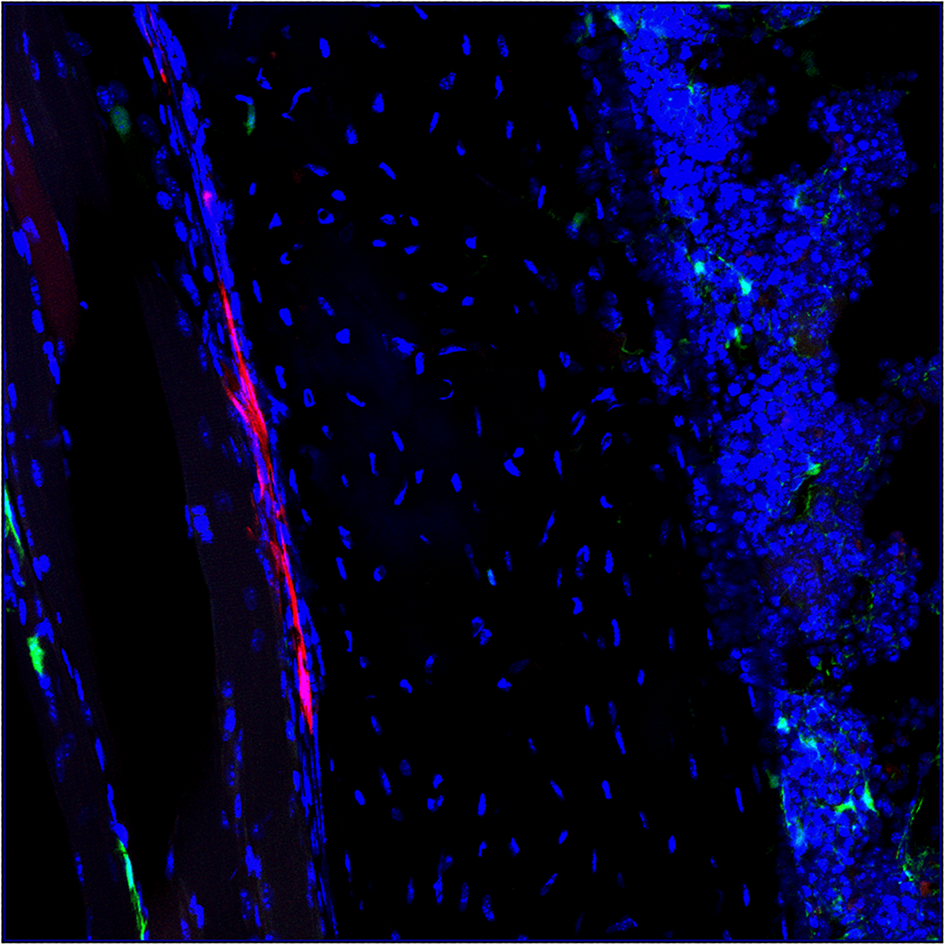

Supplement: Supplementary file 6 — Source data Fig. 4 [file 44318_2025_664_MOESM6_ESM.zip › Figure 4/4D/4D-iii.tif]

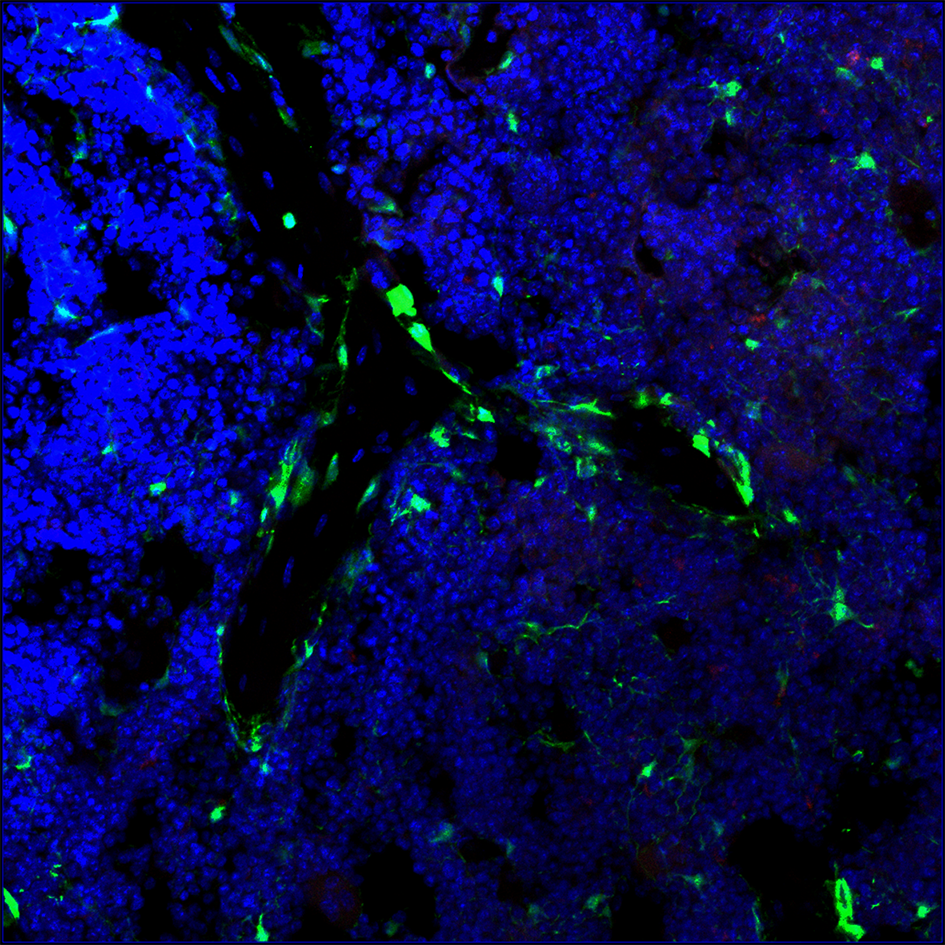

Supplement: Supplementary file 6 — Source data Fig. 4 [file 44318_2025_664_MOESM6_ESM.zip › Figure 4/4D/4D-iv.tif]

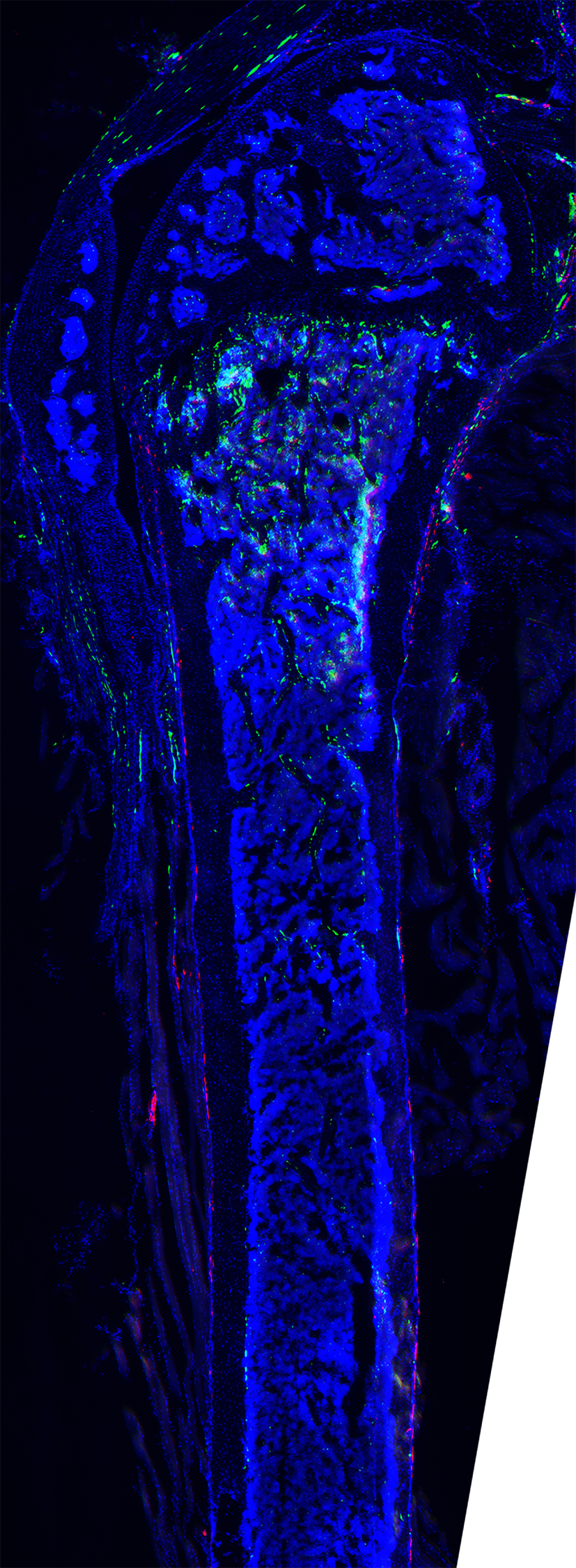

Supplement: Supplementary file 6 — Source data Fig. 4 [file 44318_2025_664_MOESM6_ESM.zip › Figure 4/4D/4D_left.tif]

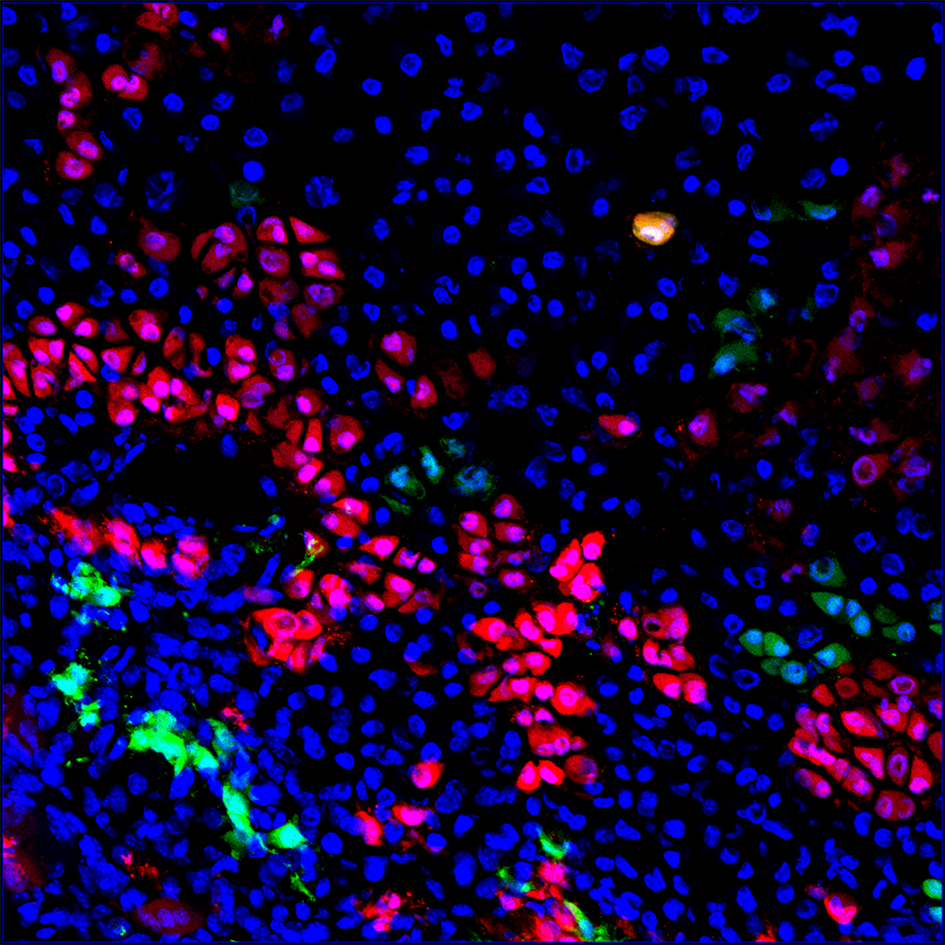

Supplement: Supplementary file 6 — Source data Fig. 4 [file 44318_2025_664_MOESM6_ESM.zip › Figure 4/4E/4E-i.tif]

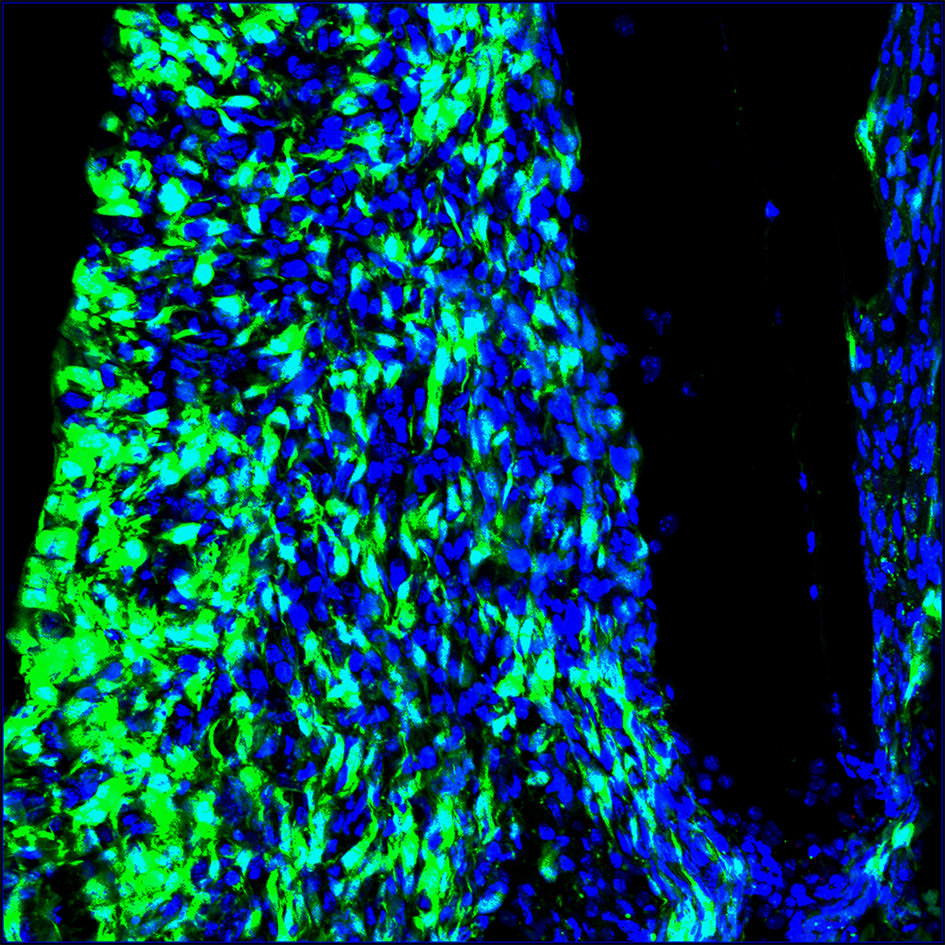

Supplement: Supplementary file 6 — Source data Fig. 4 [file 44318_2025_664_MOESM6_ESM.zip › Figure 4/4E/4E-ii.tif]

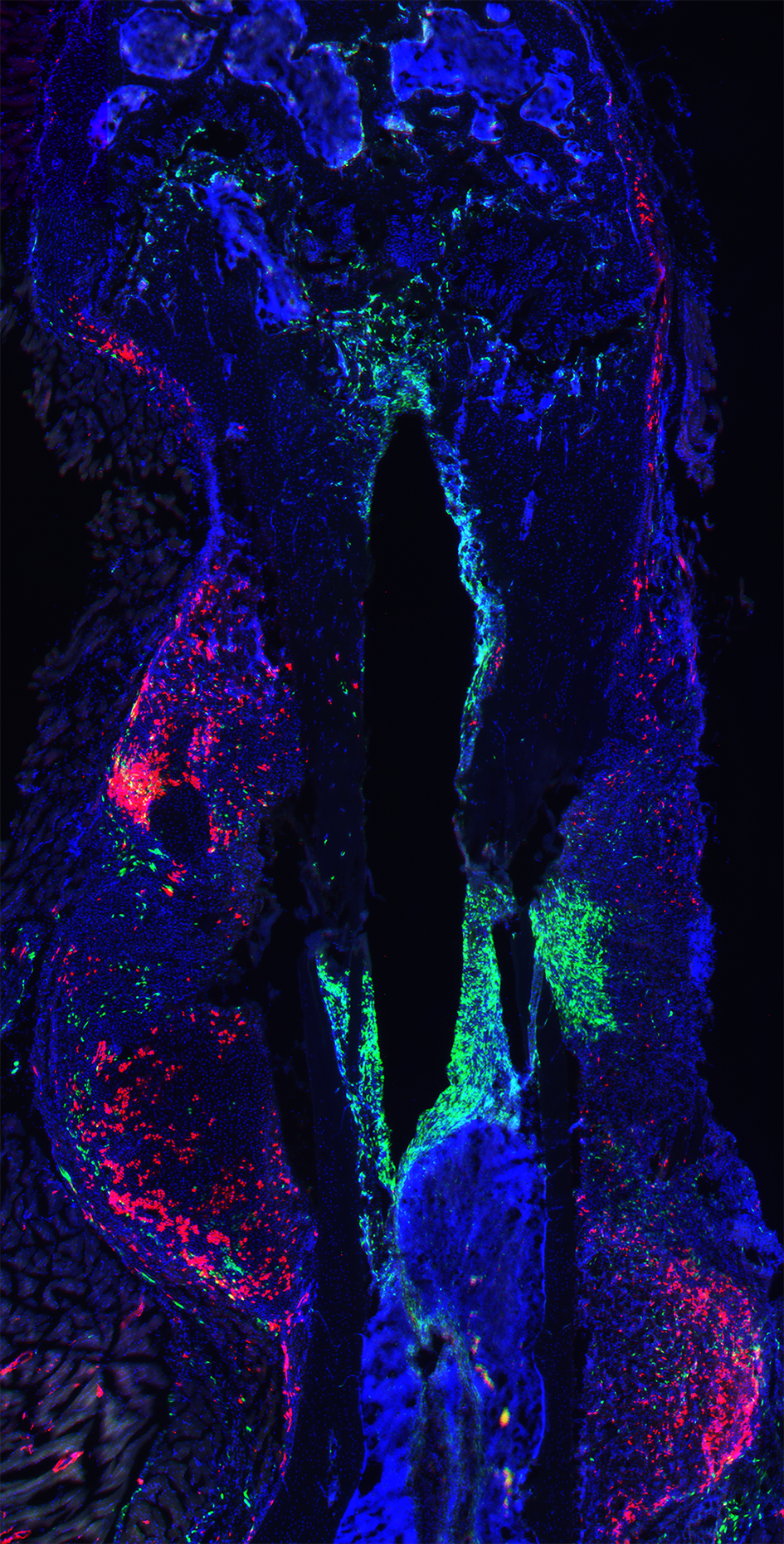

Supplement: Supplementary file 6 — Source data Fig. 4 [file 44318_2025_664_MOESM6_ESM.zip › Figure 4/4E/4E_left.tif]

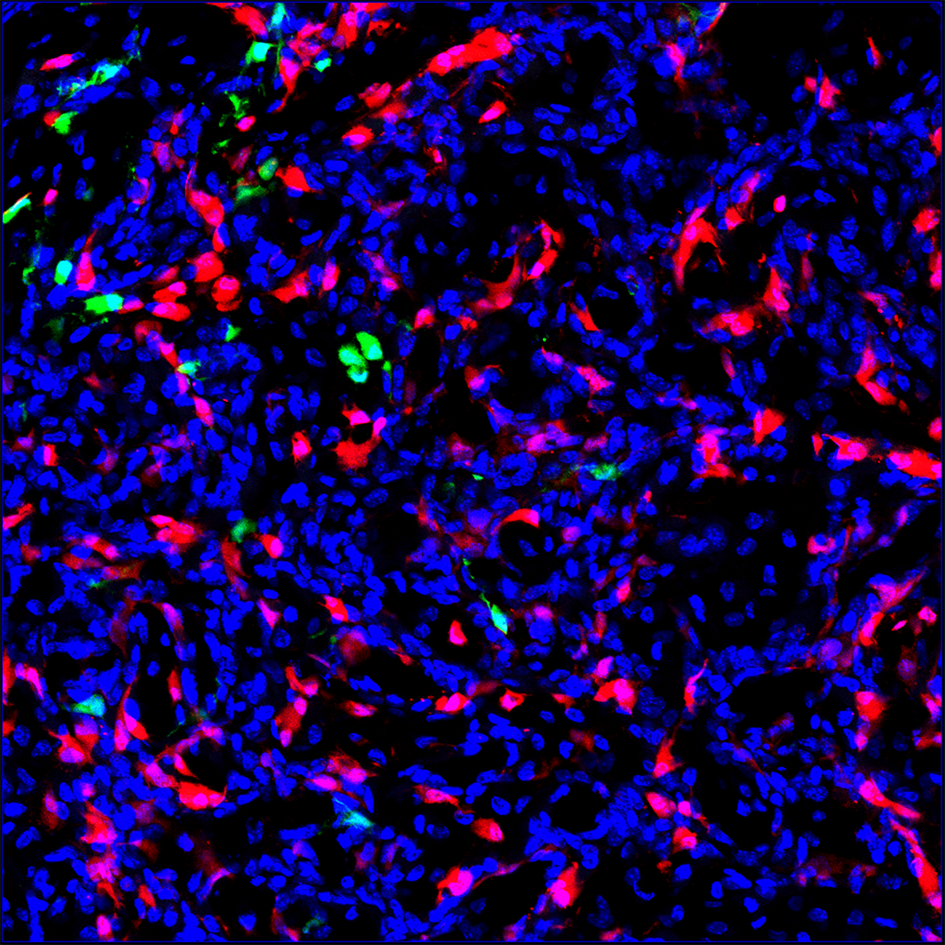

Supplement: Supplementary file 6 — Source data Fig. 4 [file 44318_2025_664_MOESM6_ESM.zip › Figure 4/4F/4F-i.tif]

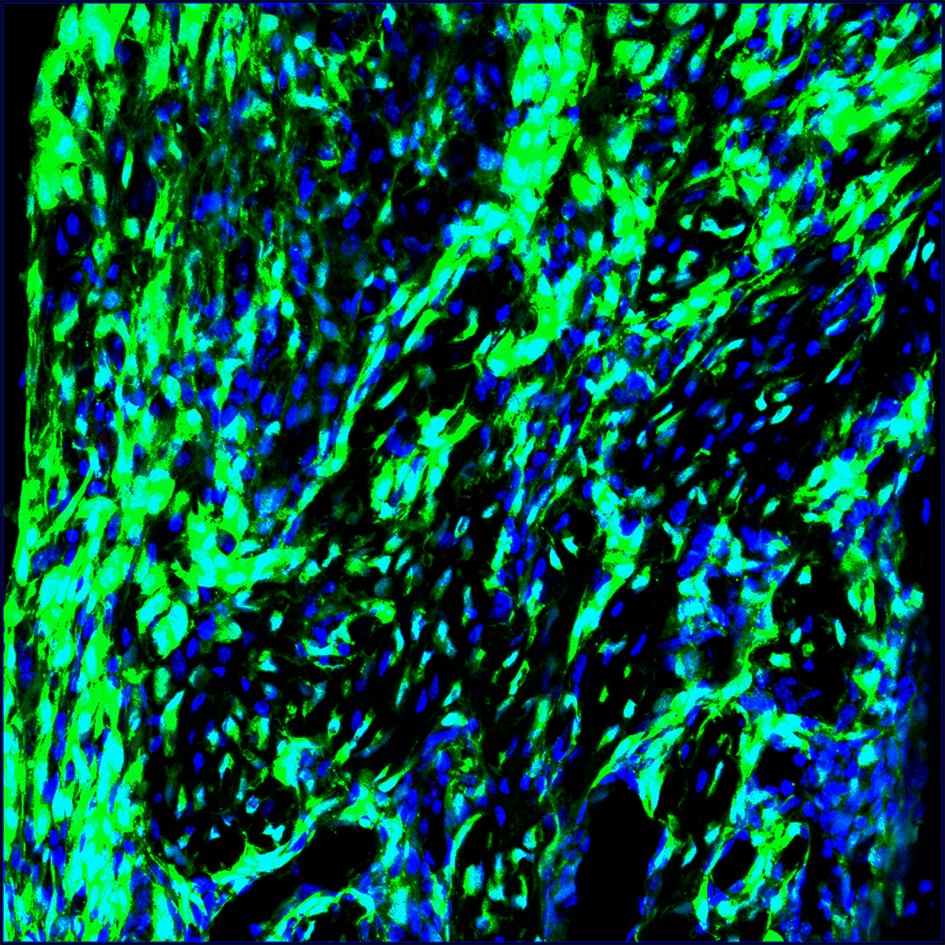

Supplement: Supplementary file 6 — Source data Fig. 4 [file 44318_2025_664_MOESM6_ESM.zip › Figure 4/4F/4F-ii.tif]

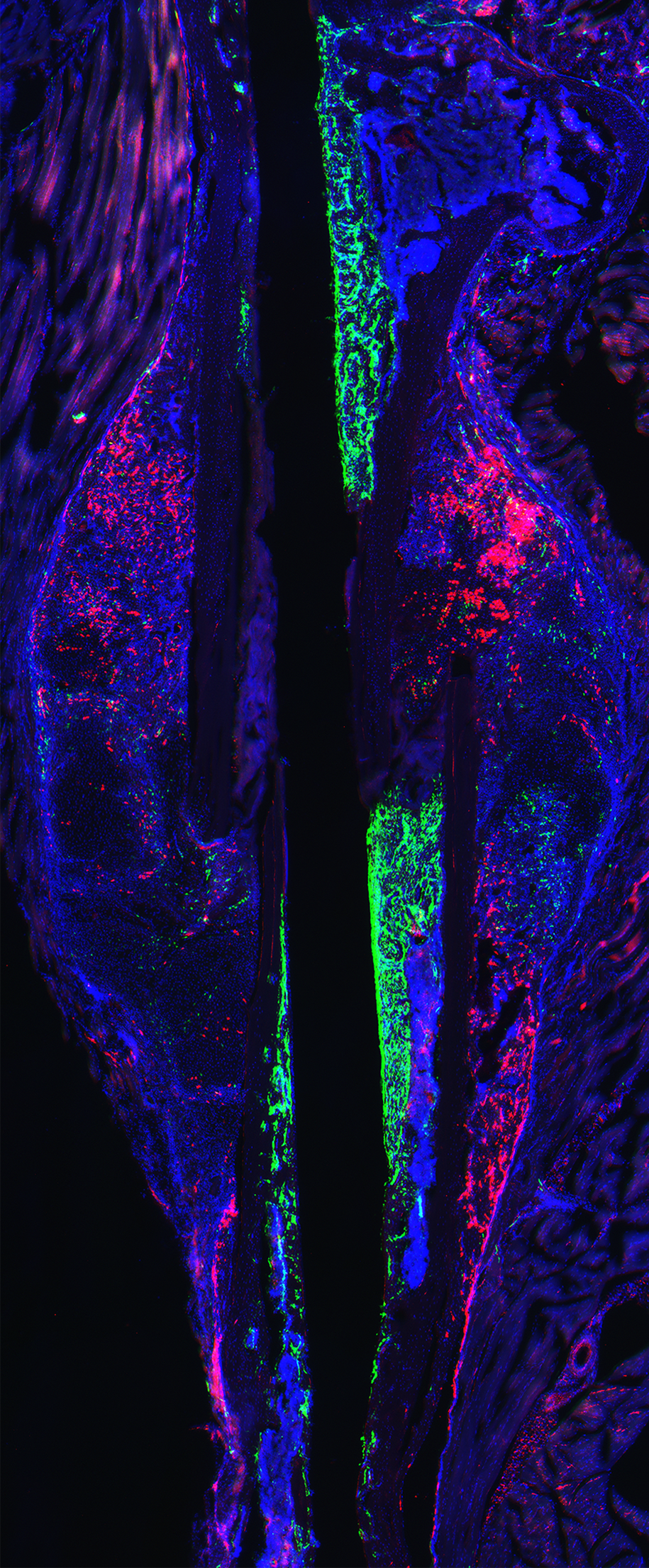

Supplement: Supplementary file 6 — Source data Fig. 4 [file 44318_2025_664_MOESM6_ESM.zip › Figure 4/4F/4F_left.tif]

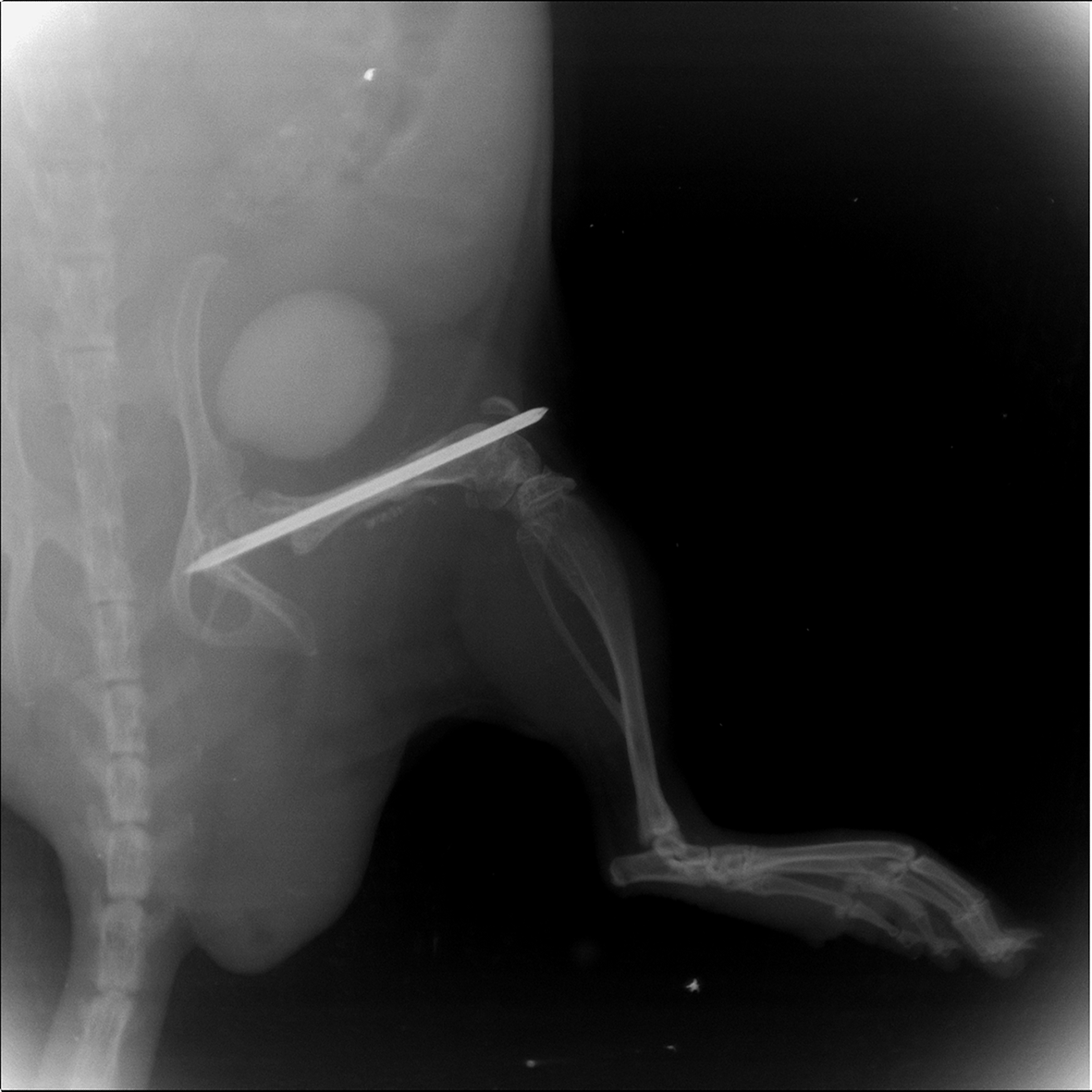

Supplement: Supplementary file 7 — Source data Fig. 5 [file 44318_2025_664_MOESM7_ESM.zip › Figure 5/5C/Prx1-cre;Bnc2_ 14 dpf.tif]

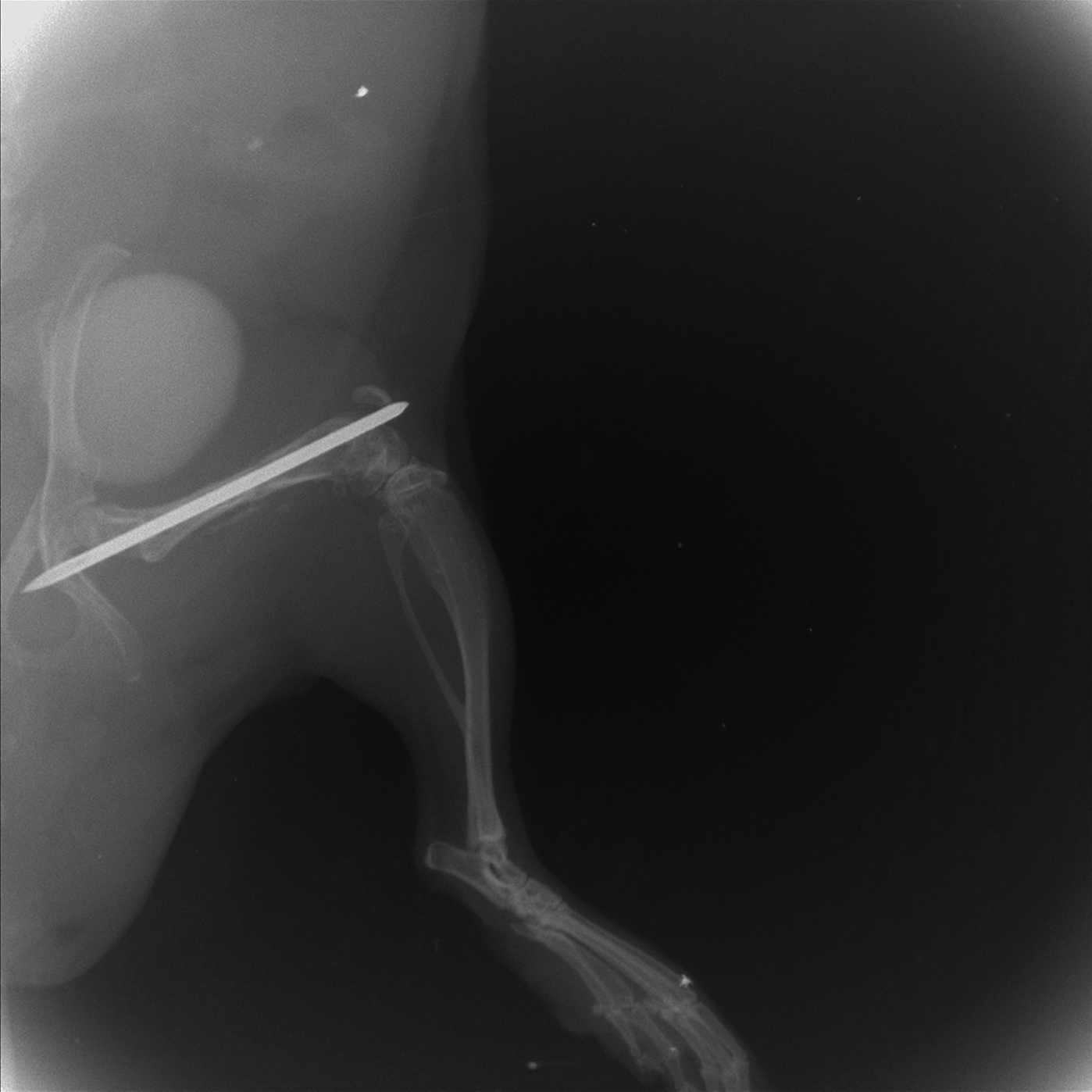

Supplement: Supplementary file 7 — Source data Fig. 5 [file 44318_2025_664_MOESM7_ESM.zip › Figure 5/5C/Prx1-cre;Bnc2_ 21 dpf.tif]

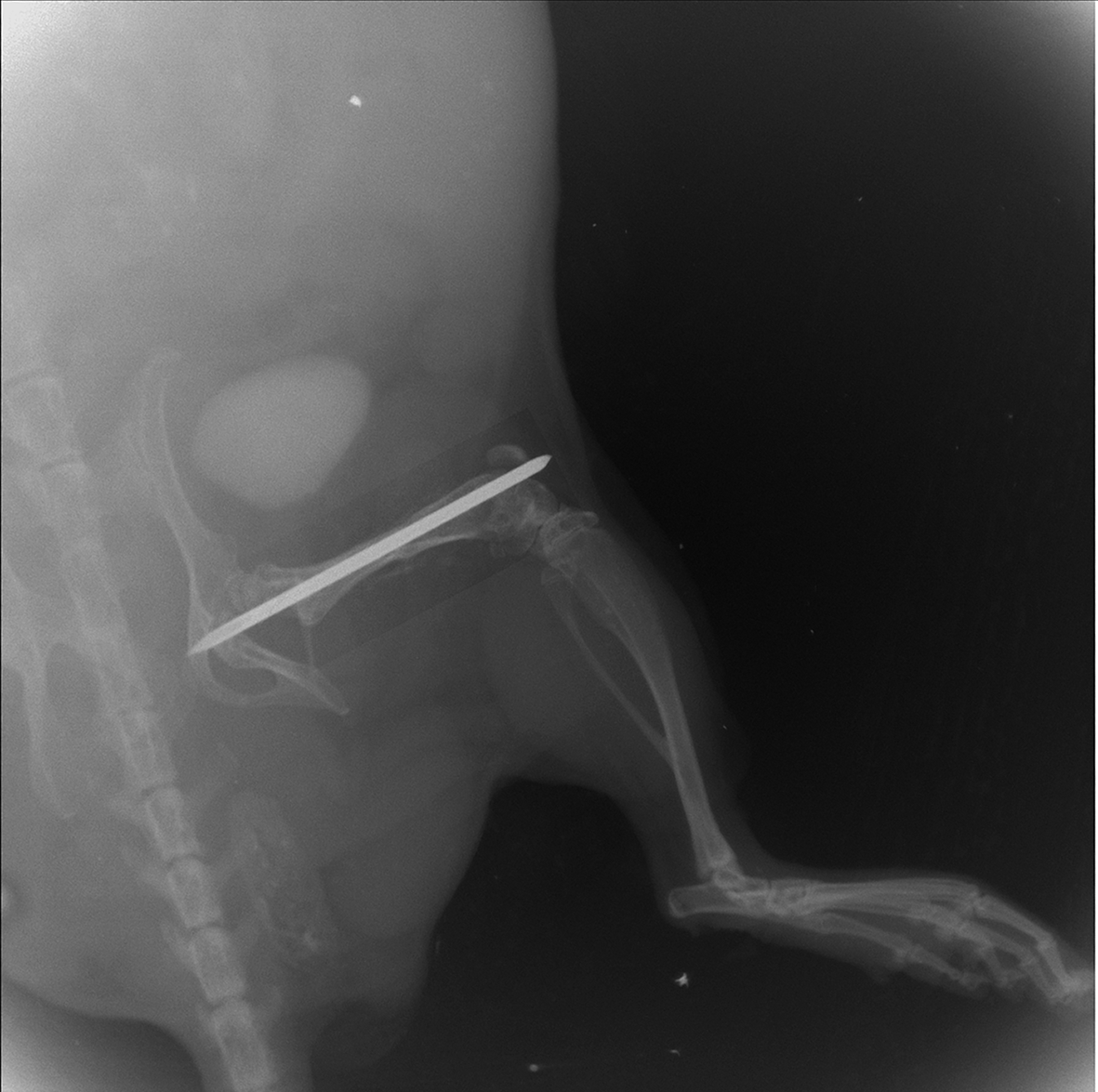

Supplement: Supplementary file 7 — Source data Fig. 5 [file 44318_2025_664_MOESM7_ESM.zip › Figure 5/5C/Prx1-cre;Bnc2_ 28 dpf.tif]

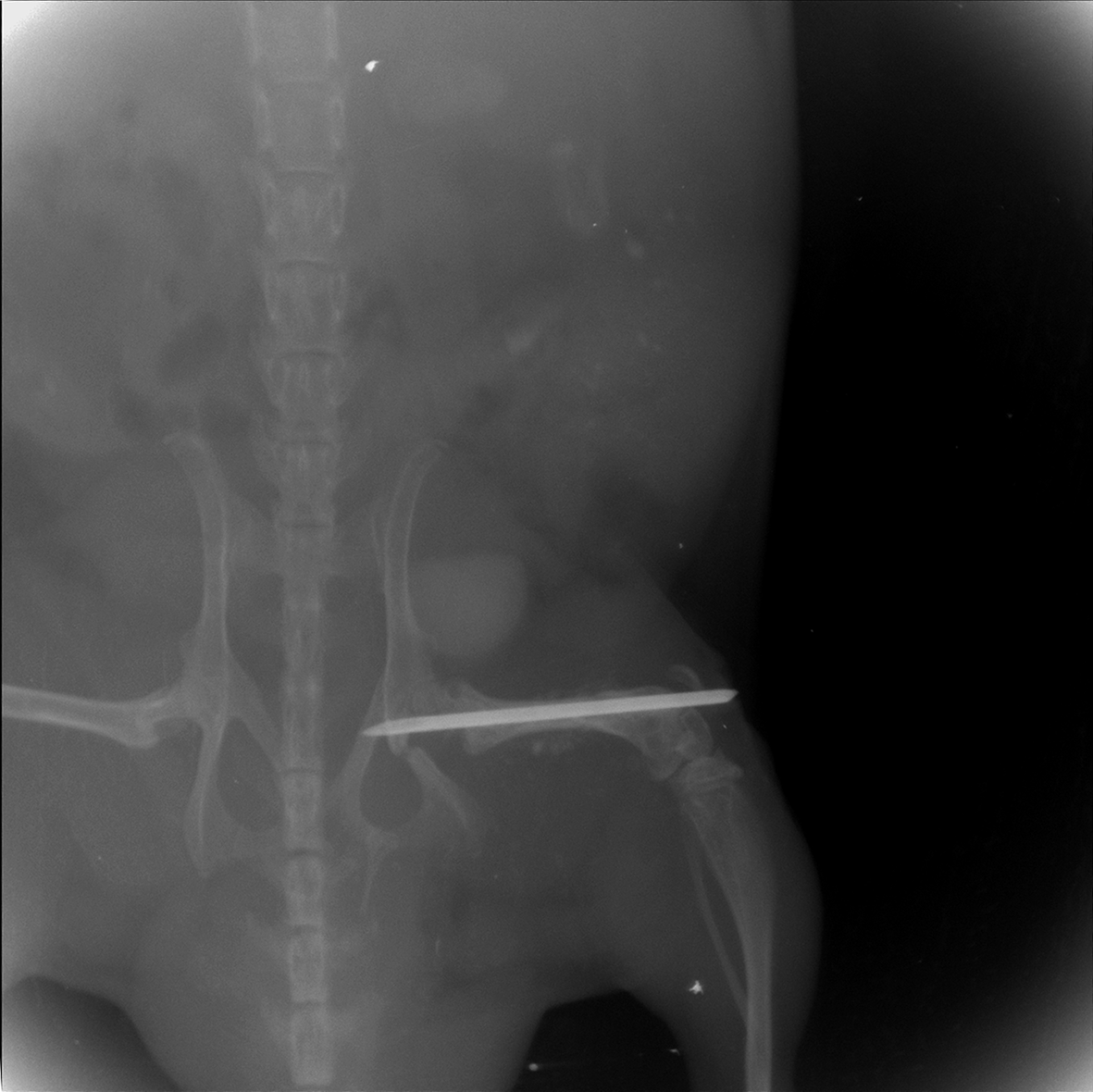

Supplement: Supplementary file 7 — Source data Fig. 5 [file 44318_2025_664_MOESM7_ESM.zip › Figure 5/5C/Prx1-cre;Bnc2_ 7 dpf.tif]

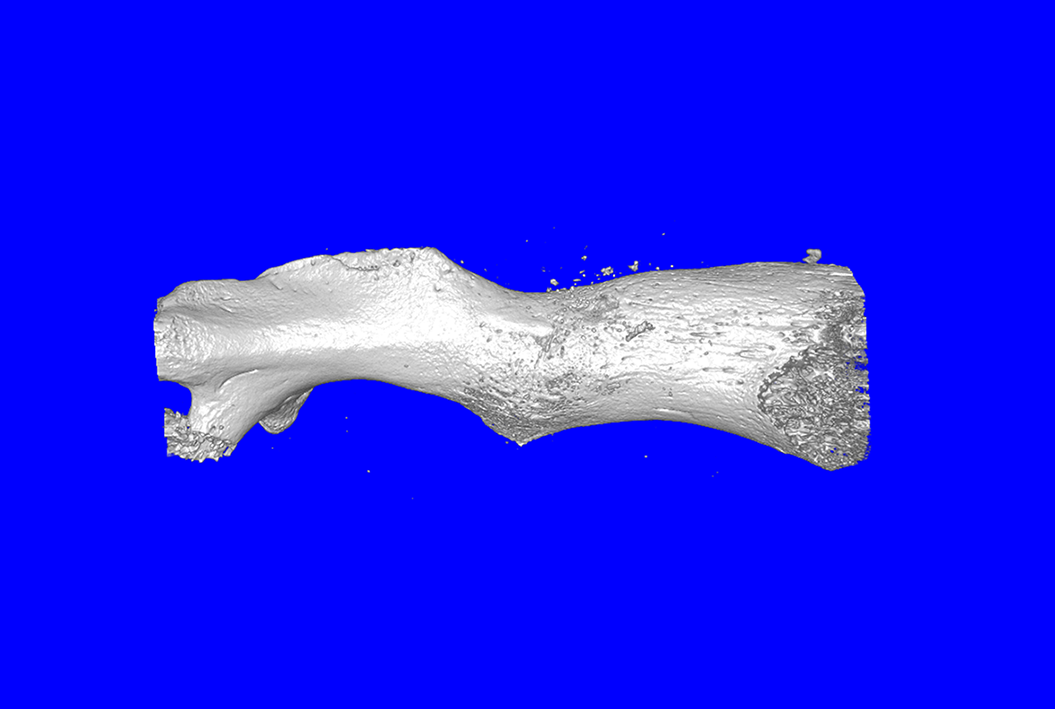

Supplement: Supplementary file 7 — Source data Fig. 5 [file 44318_2025_664_MOESM7_ESM.zip › Figure 5/5E/Ctrl.tif]

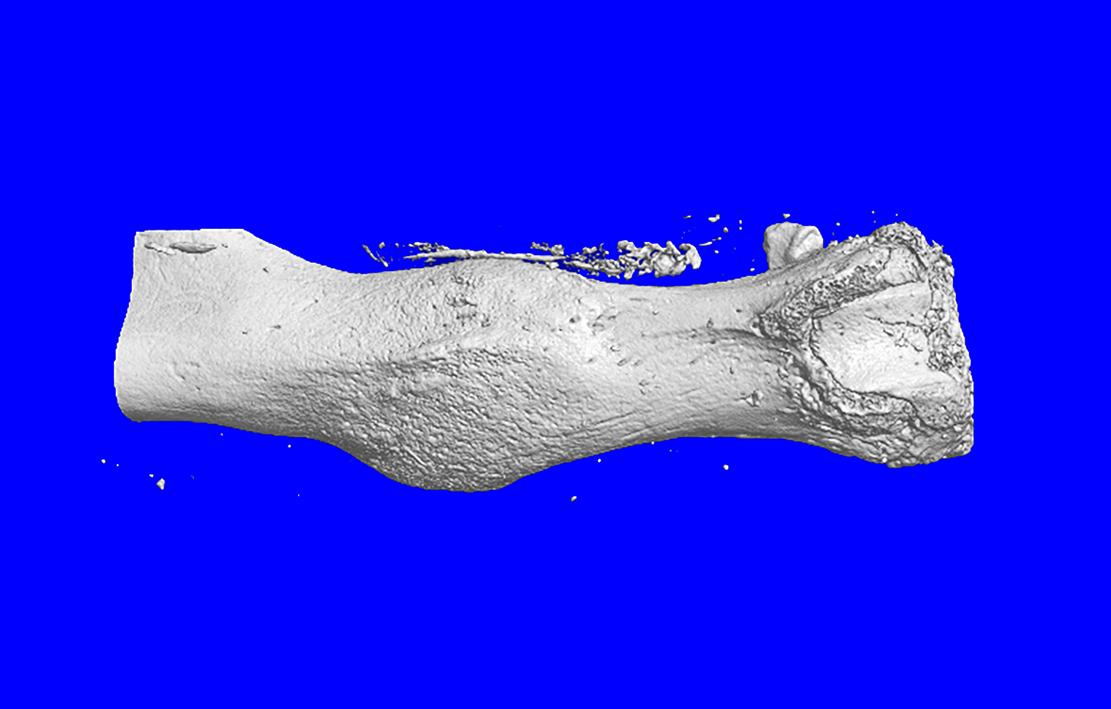

Supplement: Supplementary file 7 — Source data Fig. 5 [file 44318_2025_664_MOESM7_ESM.zip › Figure 5/5E/LepR-creER; Bnc2.tif]

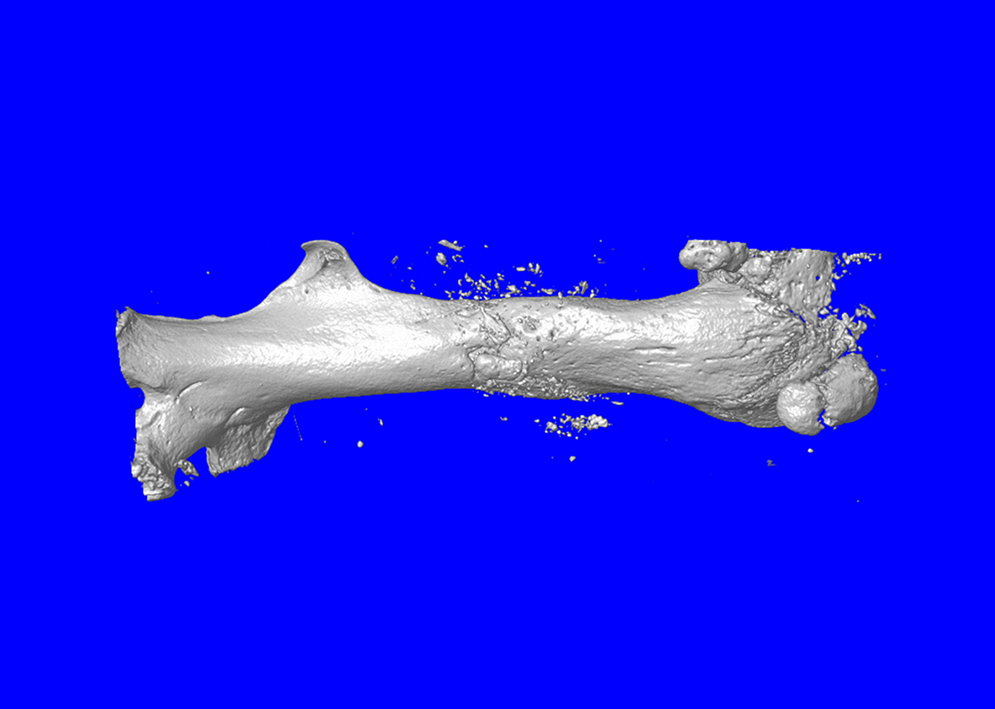

Supplement: Supplementary file 7 — Source data Fig. 5 [file 44318_2025_664_MOESM7_ESM.zip › Figure 5/5E/Prx1-cre; Bnc2.tif]

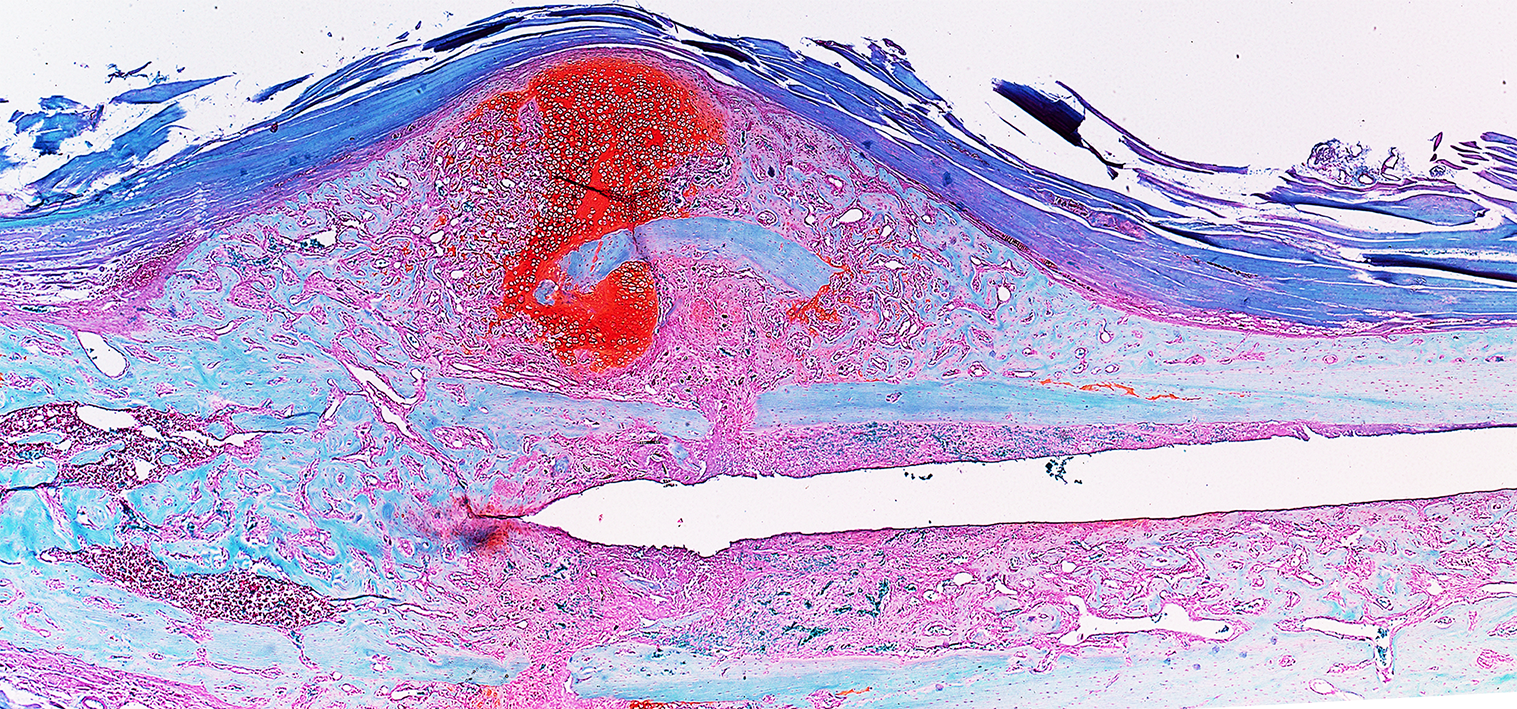

Supplement: Supplementary file 7 — Source data Fig. 5 [file 44318_2025_664_MOESM7_ESM.zip › Figure 5/5H/Ctrl_ 14 dpf.tif]

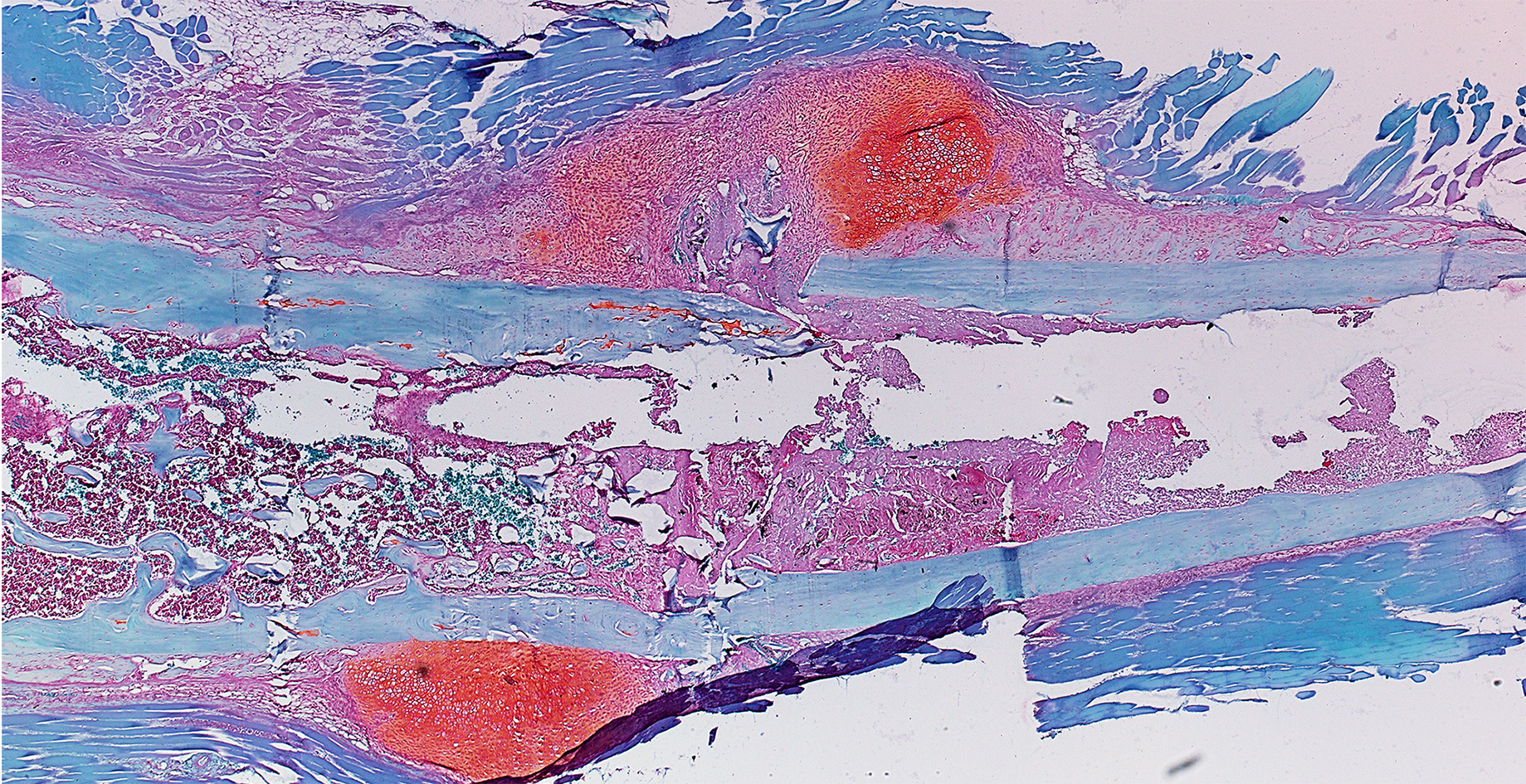

Supplement: Supplementary file 7 — Source data Fig. 5 [file 44318_2025_664_MOESM7_ESM.zip › Figure 5/5H/Ctrl_ 7 dpf.tif]

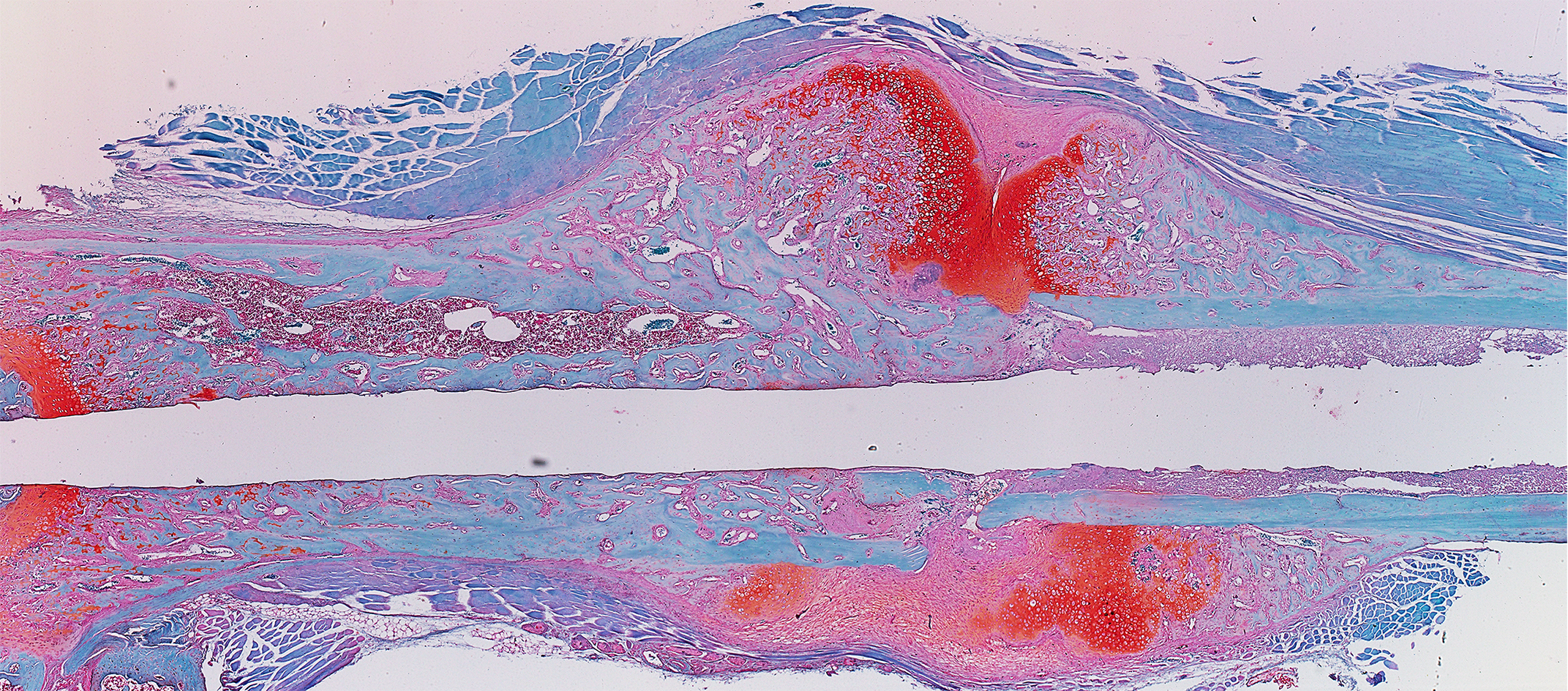

Supplement: Supplementary file 7 — Source data Fig. 5 [file 44318_2025_664_MOESM7_ESM.zip › Figure 5/5H/LepR-creER;Bnc2_ 14 dpf.tif]

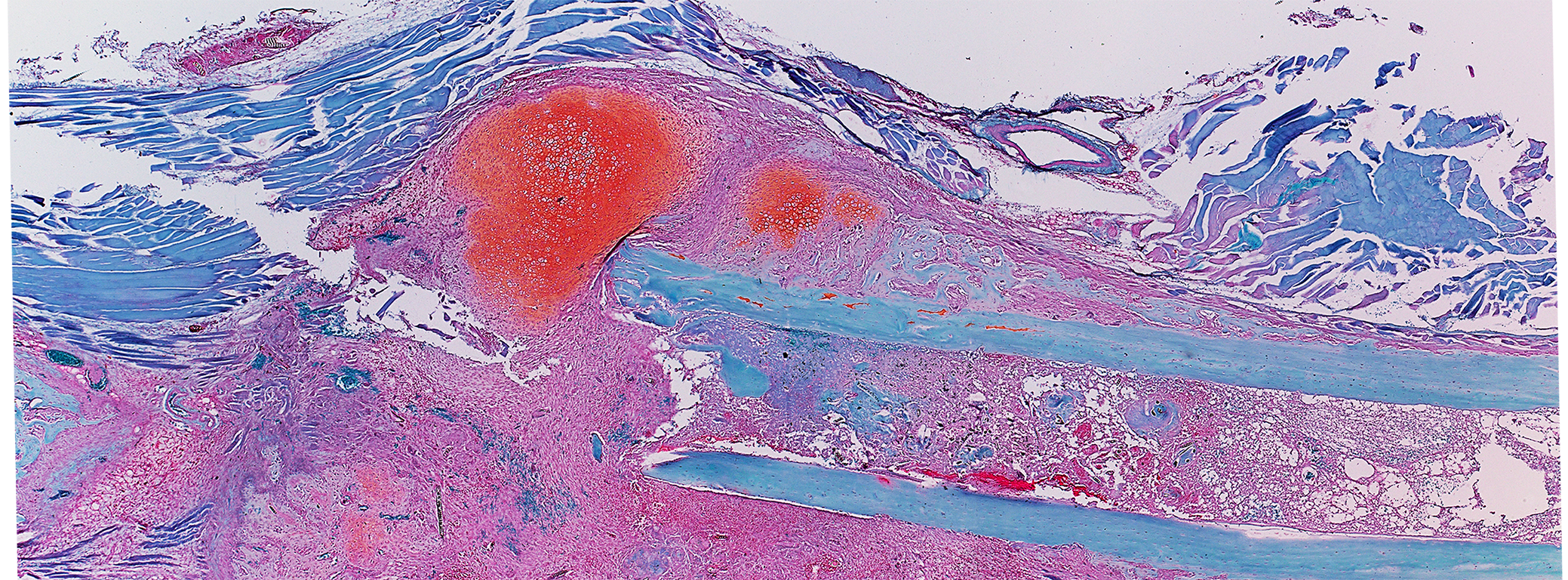

Supplement: Supplementary file 7 — Source data Fig. 5 [file 44318_2025_664_MOESM7_ESM.zip › Figure 5/5H/LepR-creER;Bnc2_ 7 dpf.tif]

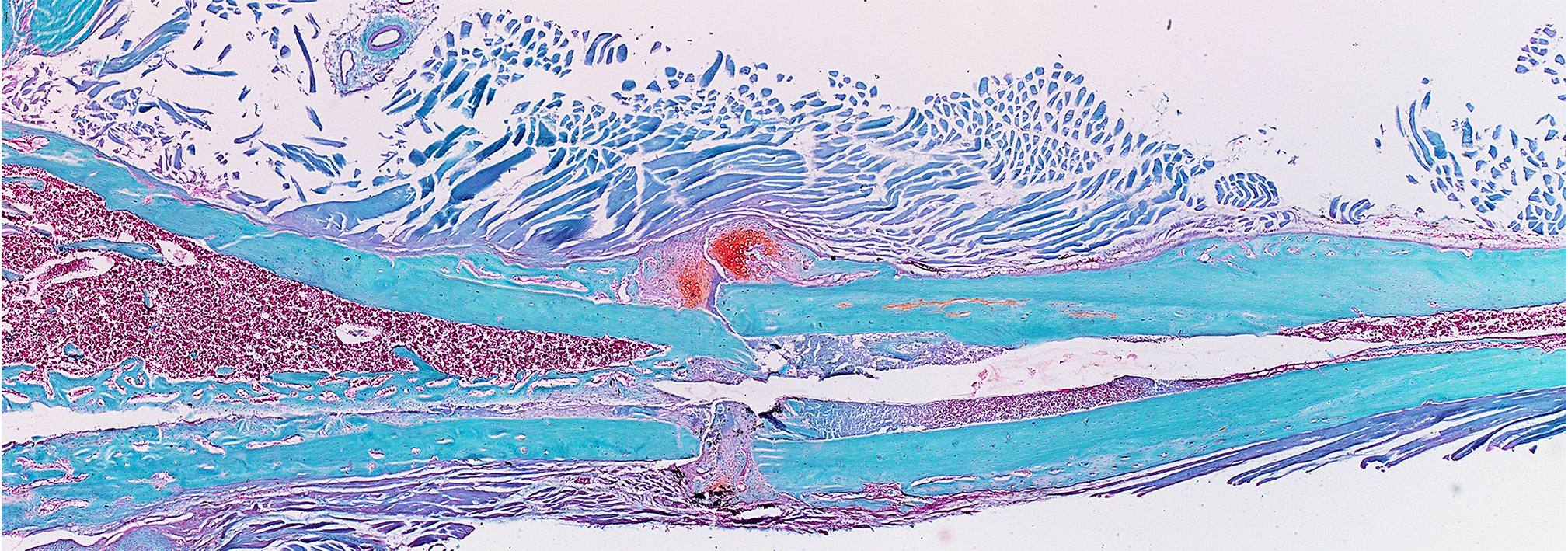

Supplement: Supplementary file 7 — Source data Fig. 5 [file 44318_2025_664_MOESM7_ESM.zip › Figure 5/5H/Prx1-cre;Bnc2_ 14 dpf.tif]

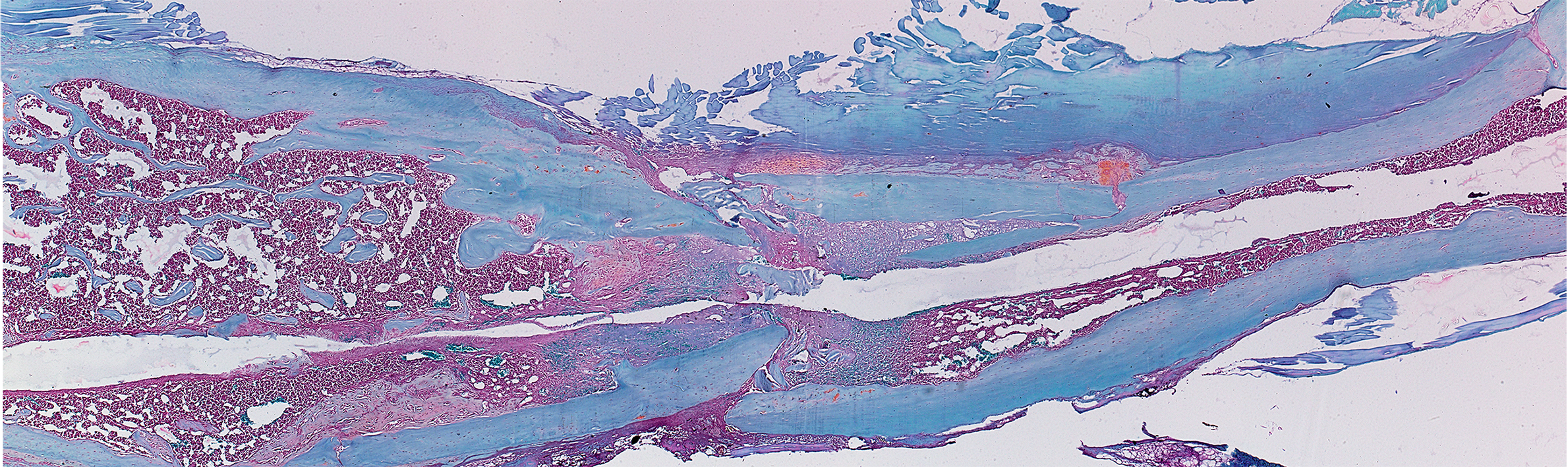

Supplement: Supplementary file 7 — Source data Fig. 5 [file 44318_2025_664_MOESM7_ESM.zip › Figure 5/5H/Prx1-cre;Bnc2_ 7 dpf.tif]

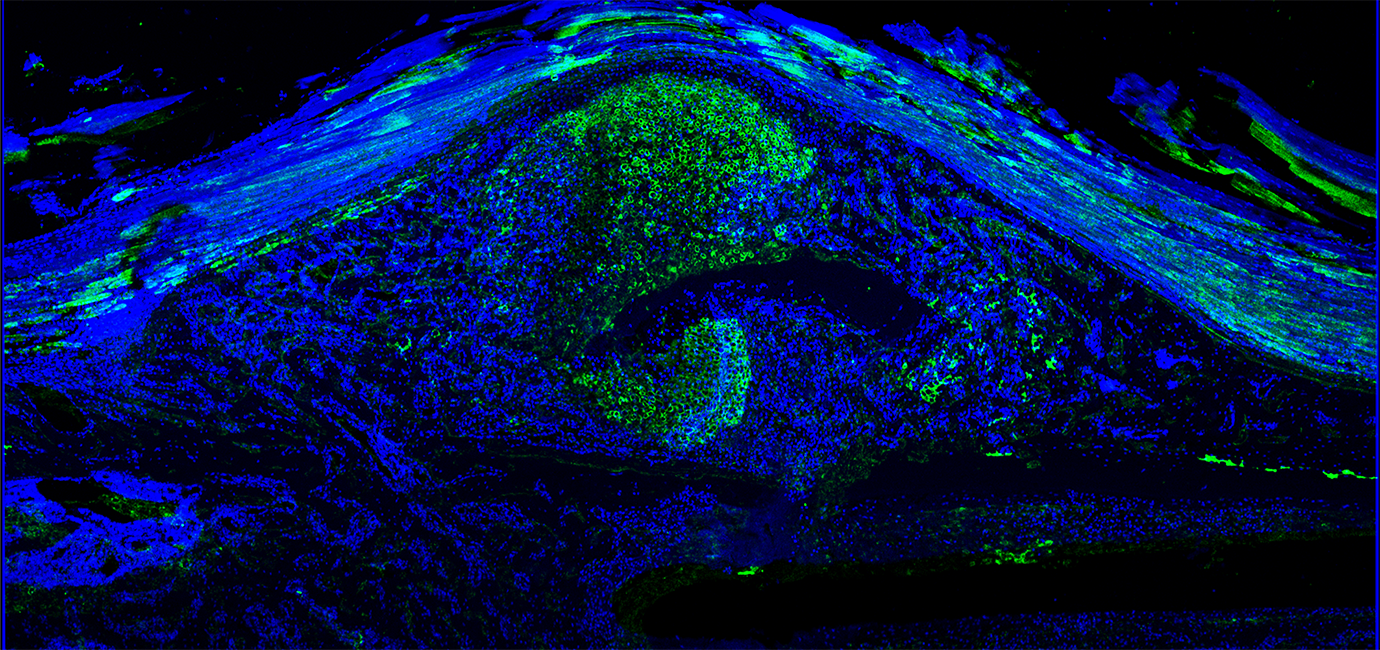

Supplement: Supplementary file 7 — Source data Fig. 5 [file 44318_2025_664_MOESM7_ESM.zip › Figure 5/5I/Ctrl_ 14 dpf.tif]

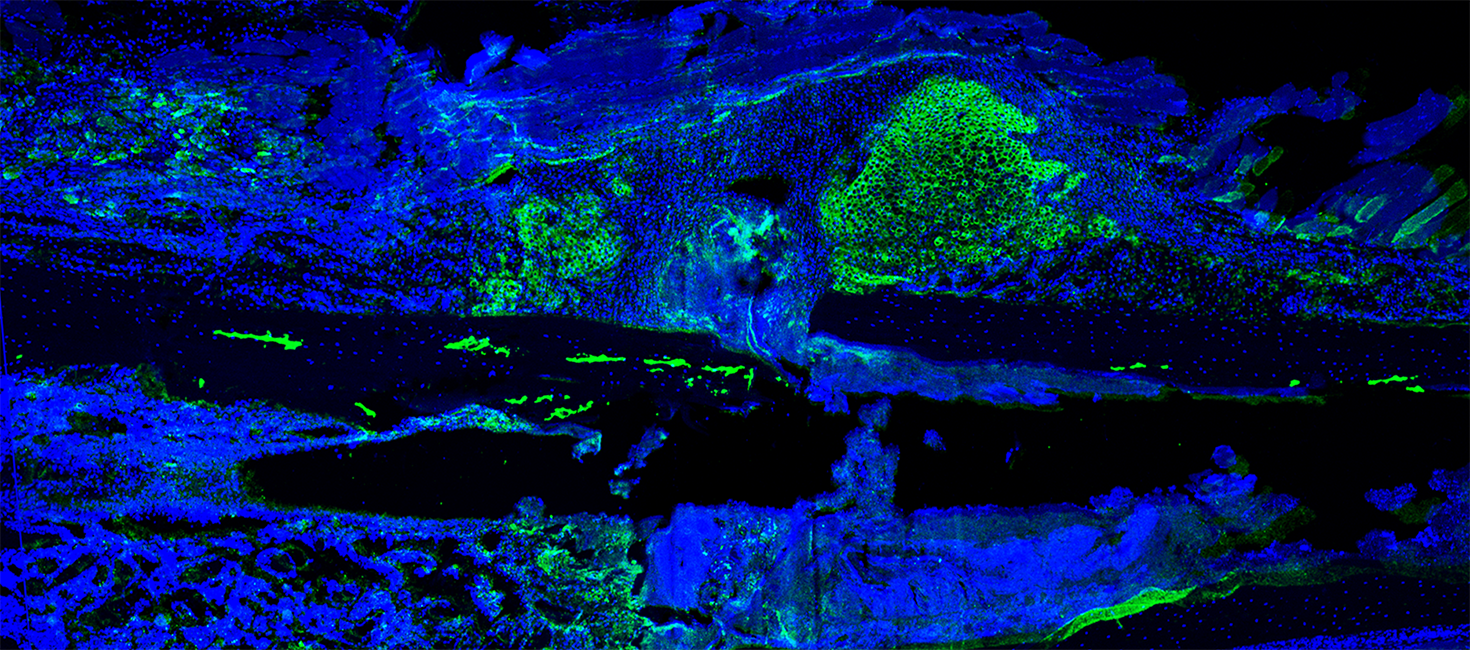

Supplement: Supplementary file 7 — Source data Fig. 5 [file 44318_2025_664_MOESM7_ESM.zip › Figure 5/5I/Ctrl_ 7 dpf.tif]

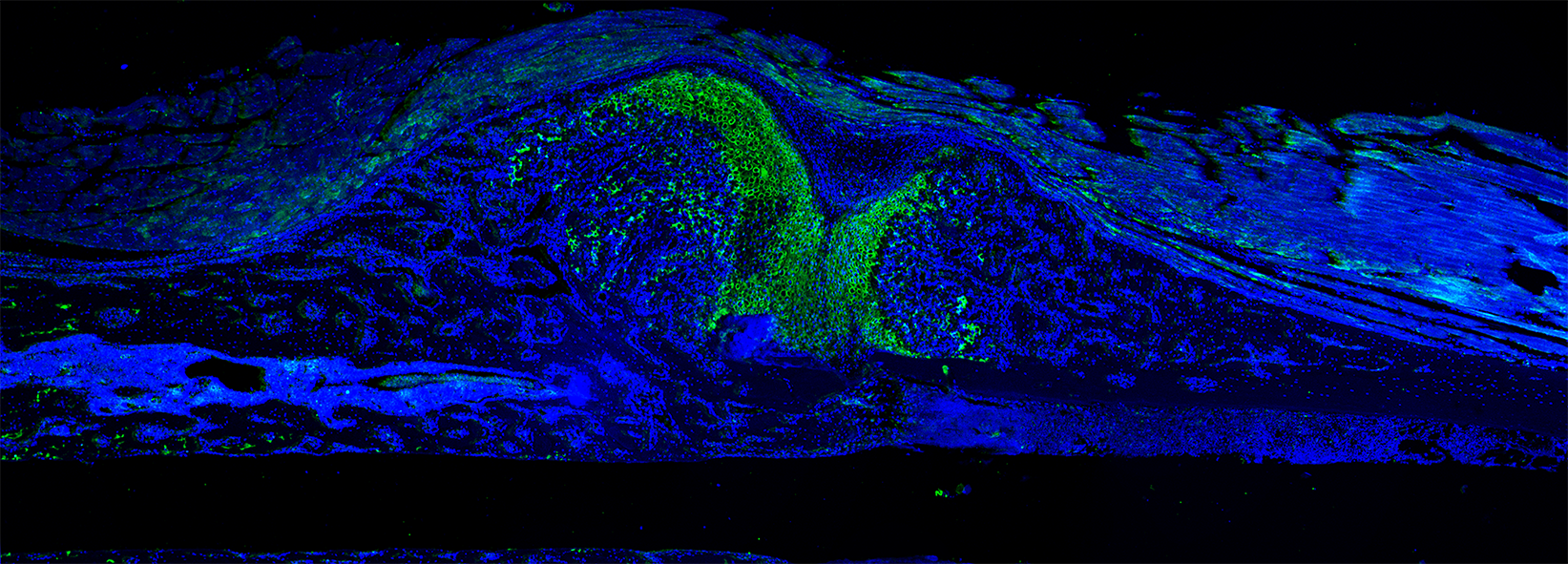

Supplement: Supplementary file 7 — Source data Fig. 5 [file 44318_2025_664_MOESM7_ESM.zip › Figure 5/5I/LepR-creER;Bnc2_ 14 dpf.tif]

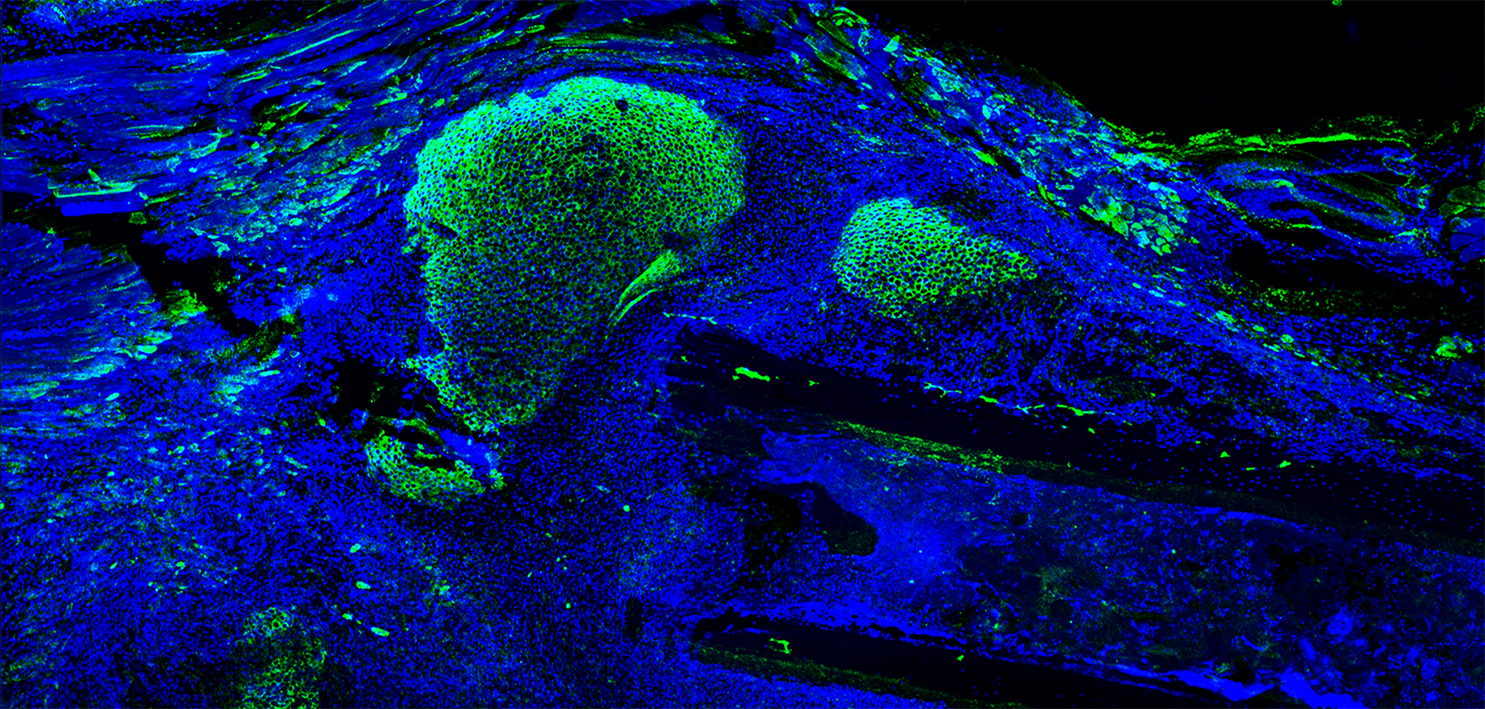

Supplement: Supplementary file 7 — Source data Fig. 5 [file 44318_2025_664_MOESM7_ESM.zip › Figure 5/5I/LepR-creER;Bnc2_ 7 dpf.tif]

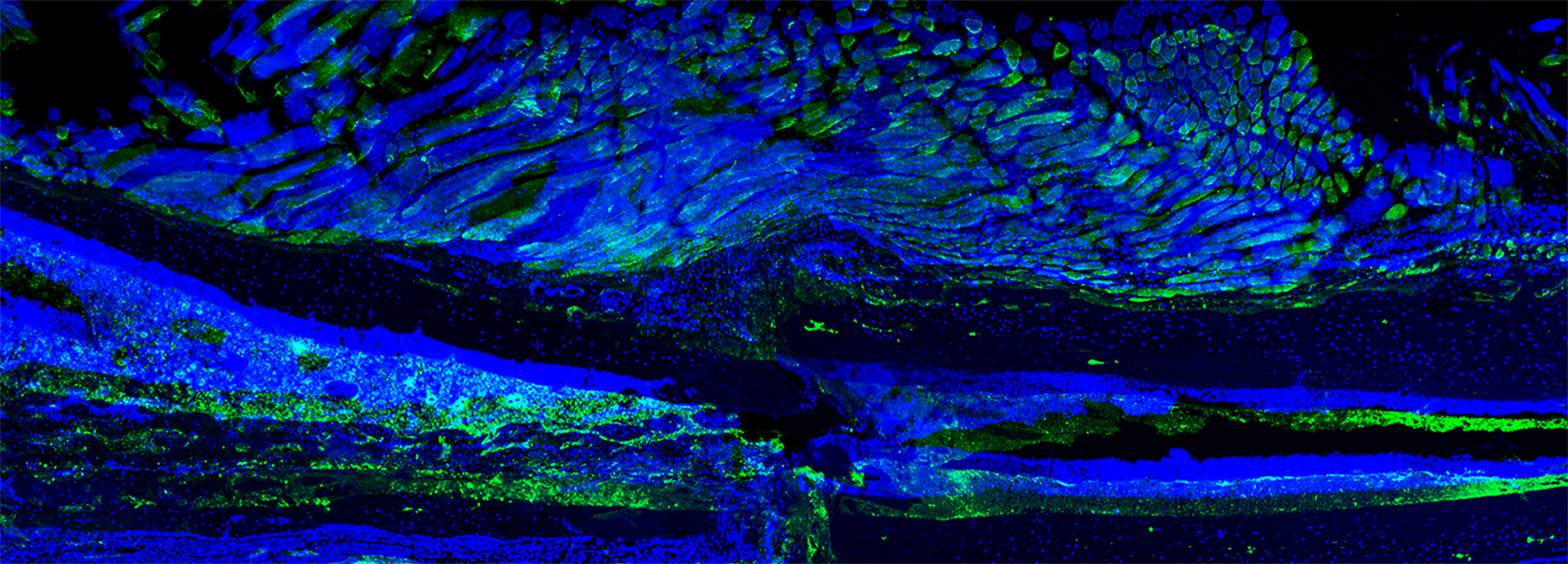

Supplement: Supplementary file 7 — Source data Fig. 5 [file 44318_2025_664_MOESM7_ESM.zip › Figure 5/5I/Prx1-cre;Bnc2_ 14 dpf.tif]

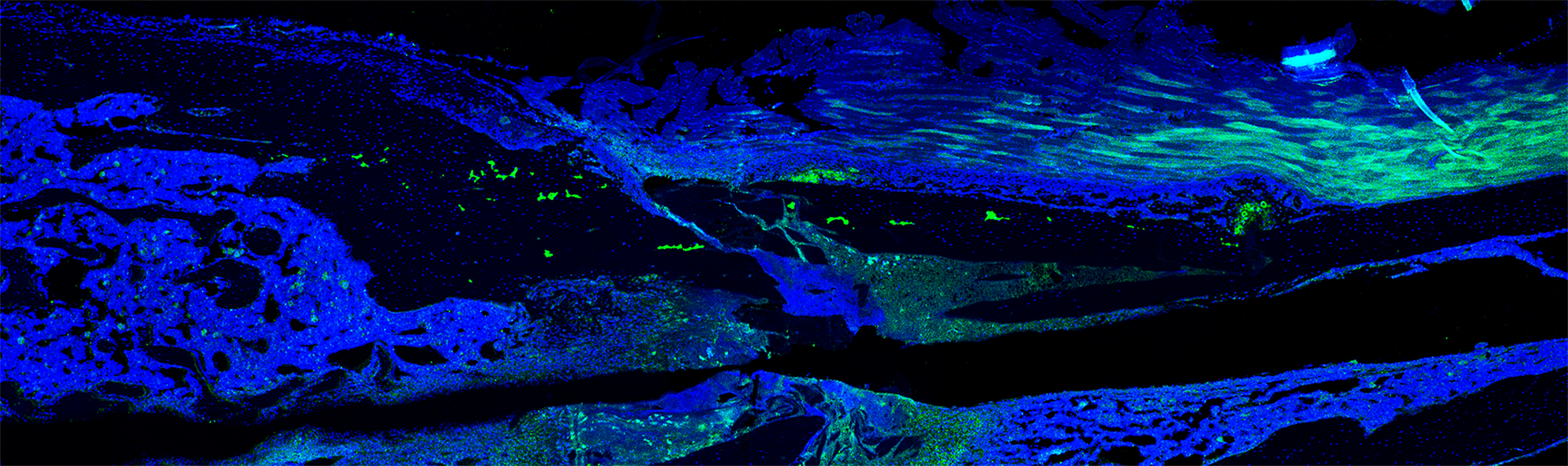

Supplement: Supplementary file 7 — Source data Fig. 5 [file 44318_2025_664_MOESM7_ESM.zip › Figure 5/5I/Prx1-cre;Bnc2_ 7 dpf.tif]

Periosteum

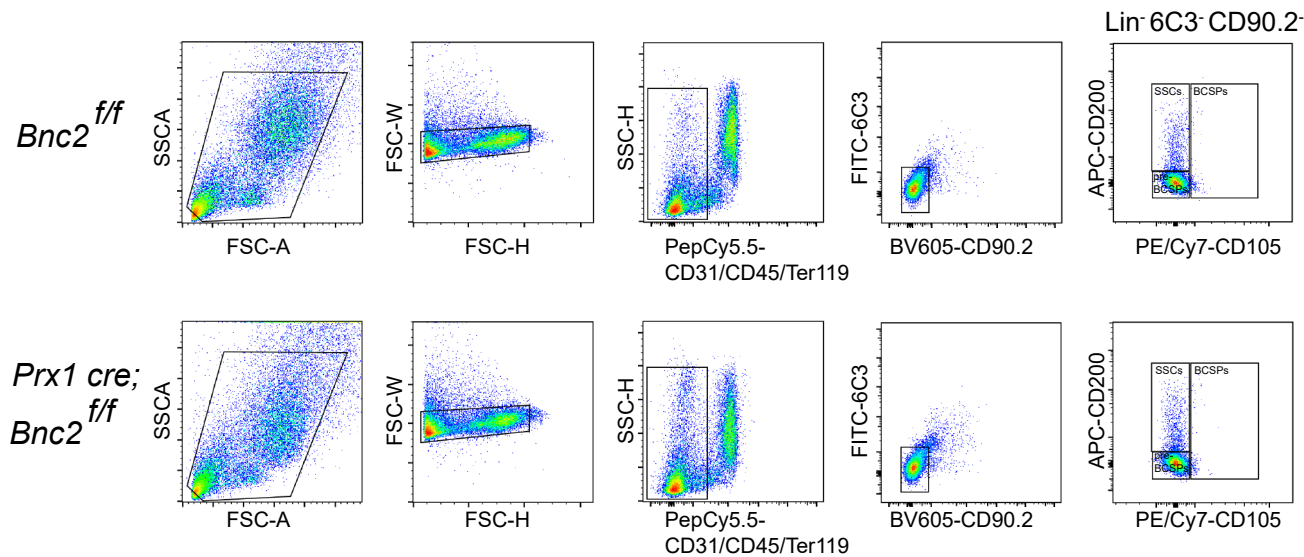

Callus

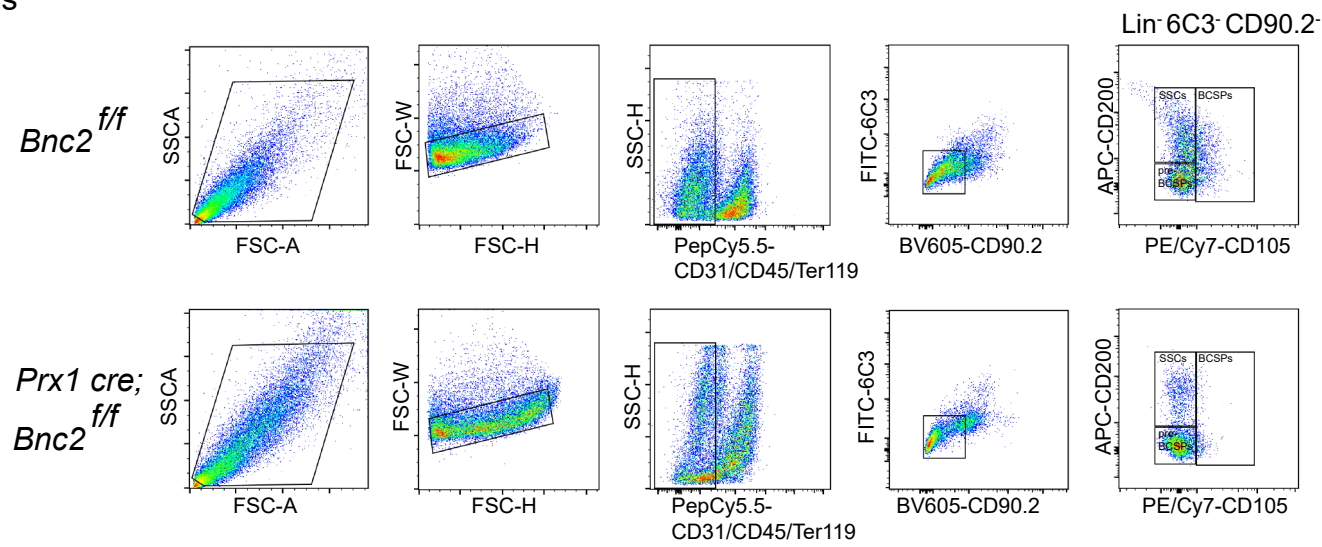

Supplement: Supplementary file 8 — Source data Fig. 6 [file 44318_2025_664_MOESM8_ESM.zip › Figure 6/6B/Flow cytometry analysis of the proportion of SSCs in Prx1-cre; Bnc2 mice.pdf]

Periosteum

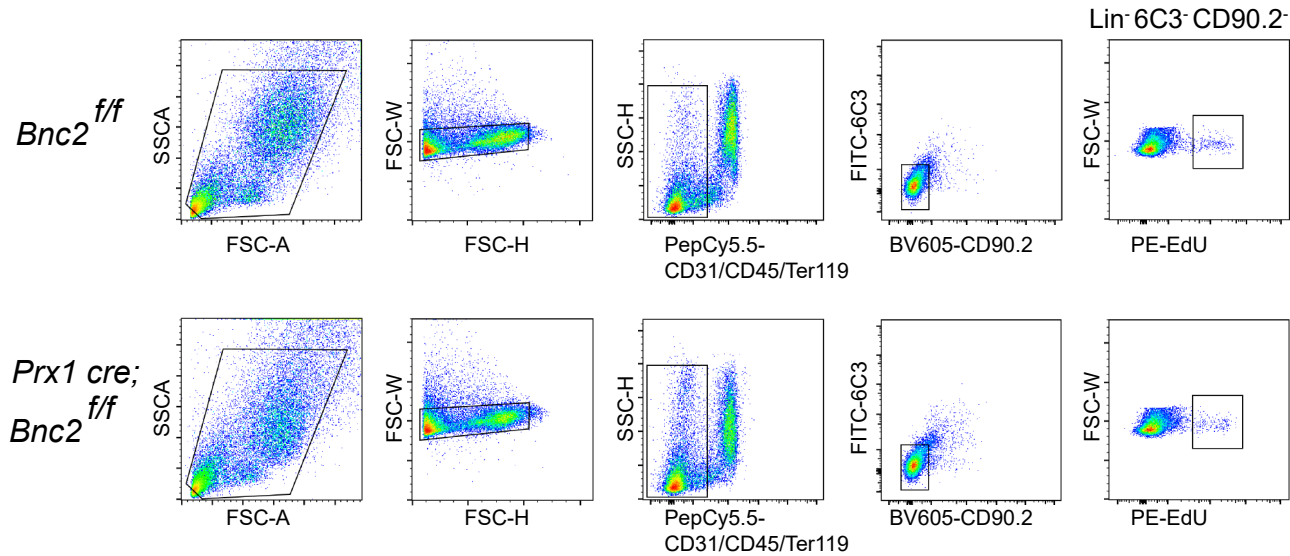

Callus

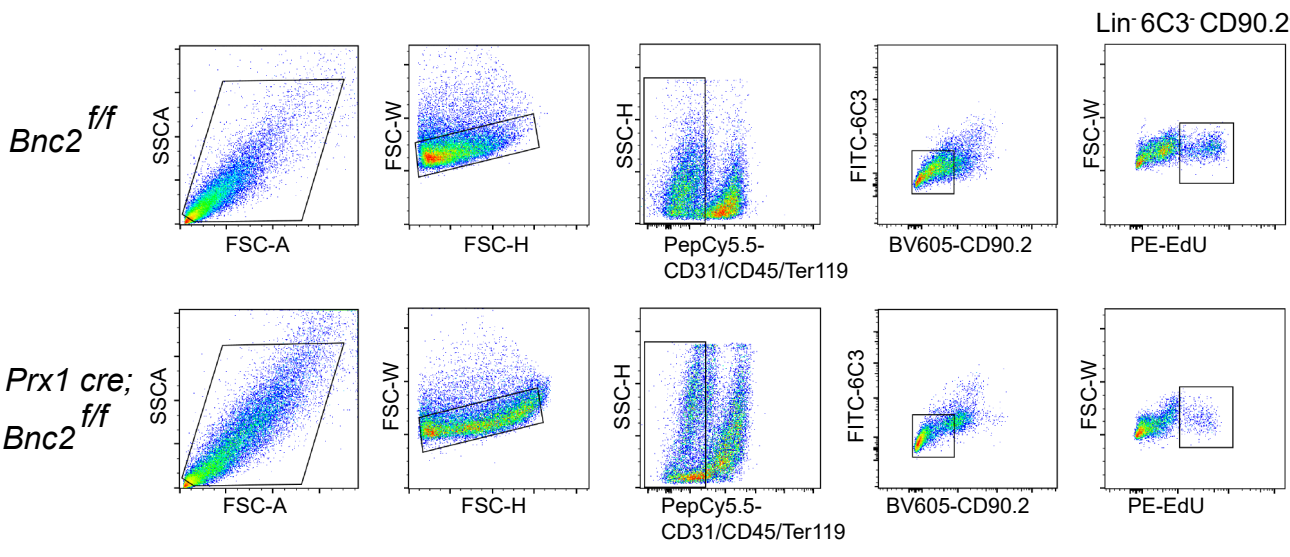

Supplement: Supplementary file 8 — Source data Fig. 6 [file 44318_2025_664_MOESM8_ESM.zip › Figure 6/6E/Flow cytometry analysis of the proportion of EdU+ cells in Prx1-cre; Bnc2 mice.pdf]

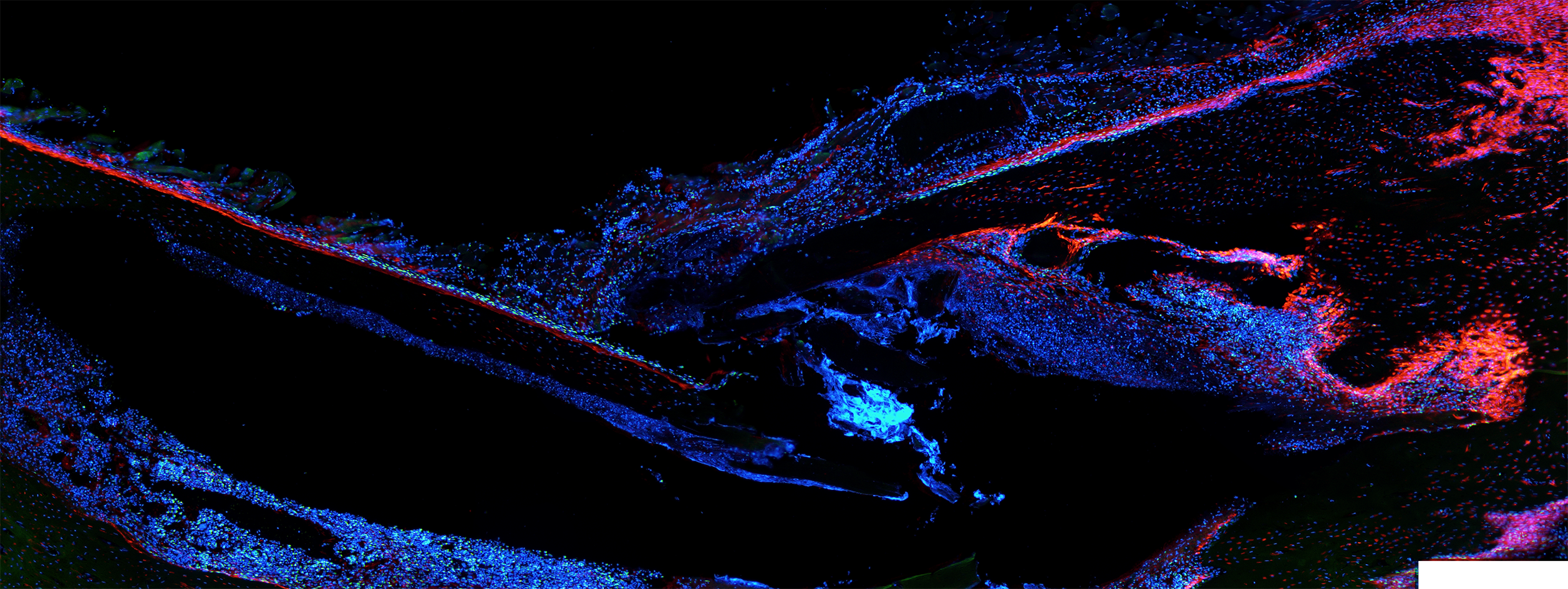

Supplement: Supplementary file 8 — Source data Fig. 6 [file 44318_2025_664_MOESM8_ESM.zip › Figure 6/6G/Prx1-cre;Bnc2 f+ _ left.tif]

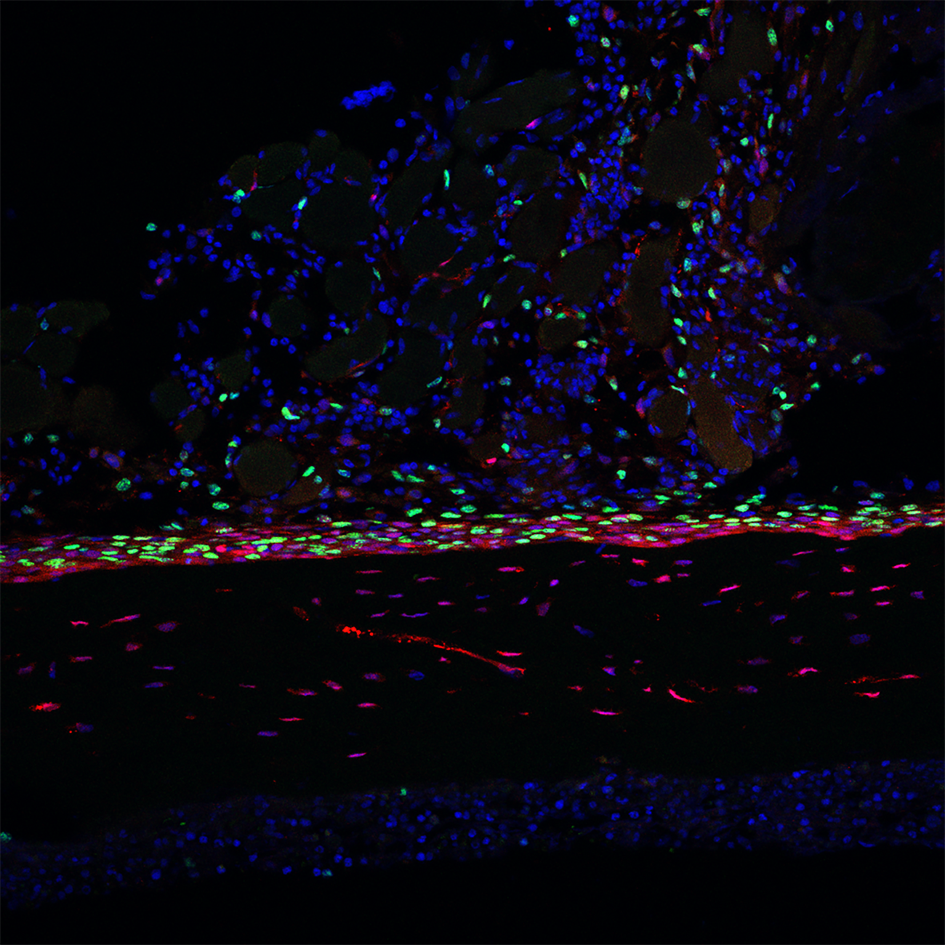

Supplement: Supplementary file 8 — Source data Fig. 6 [file 44318_2025_664_MOESM8_ESM.zip › Figure 6/6G/Prx1-cre;Bnc2 f+ _ right.tif]

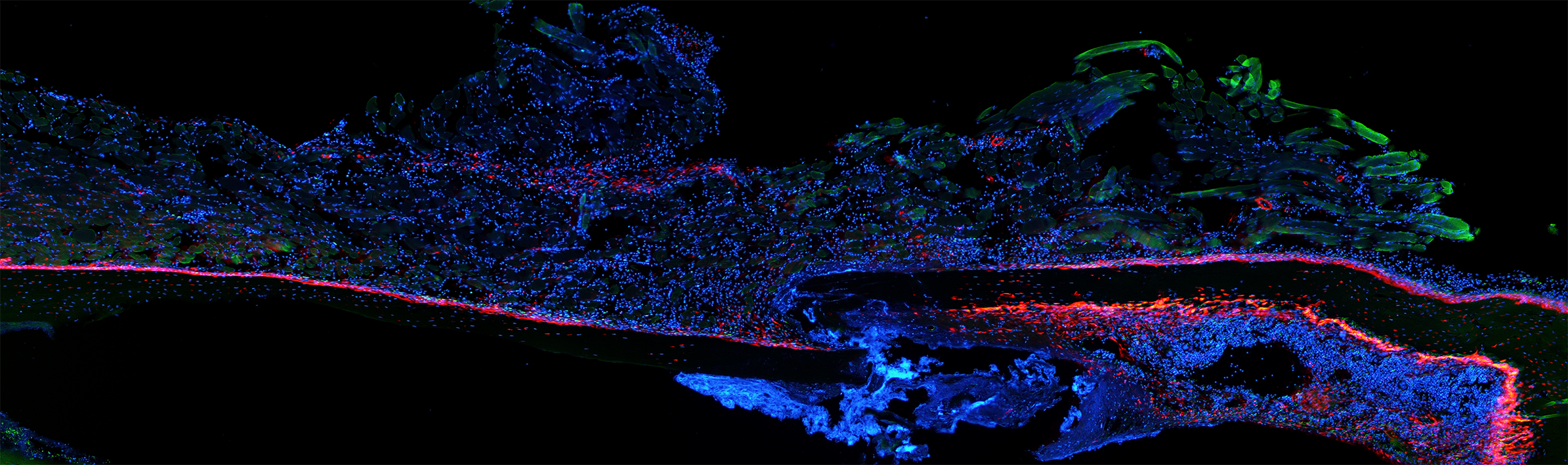

Supplement: Supplementary file 8 — Source data Fig. 6 [file 44318_2025_664_MOESM8_ESM.zip › Figure 6/6G/Prx1-cre;Bnc2 ff _ left.tif]

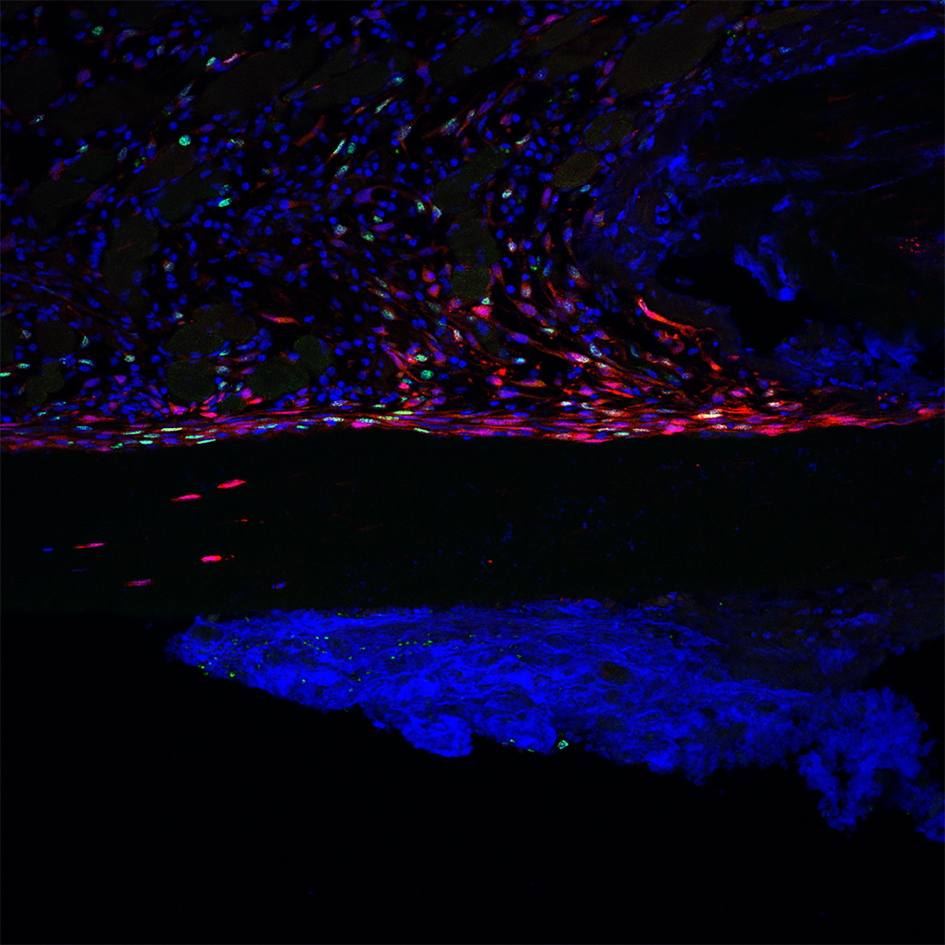

Supplement: Supplementary file 8 — Source data Fig. 6 [file 44318_2025_664_MOESM8_ESM.zip › Figure 6/6G/Prx1-cre;Bnc2 ff _ right.tif]

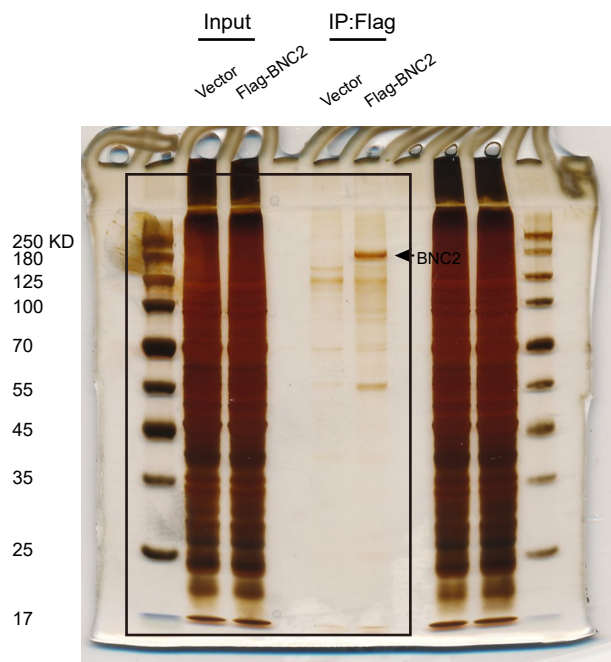

Supplement: Supplementary file 9 — Source data Fig. 7 [file 44318_2025_664_MOESM9_ESM.zip › Figure 7/7A/Silver staing of BNC2 pull-down.pdf]

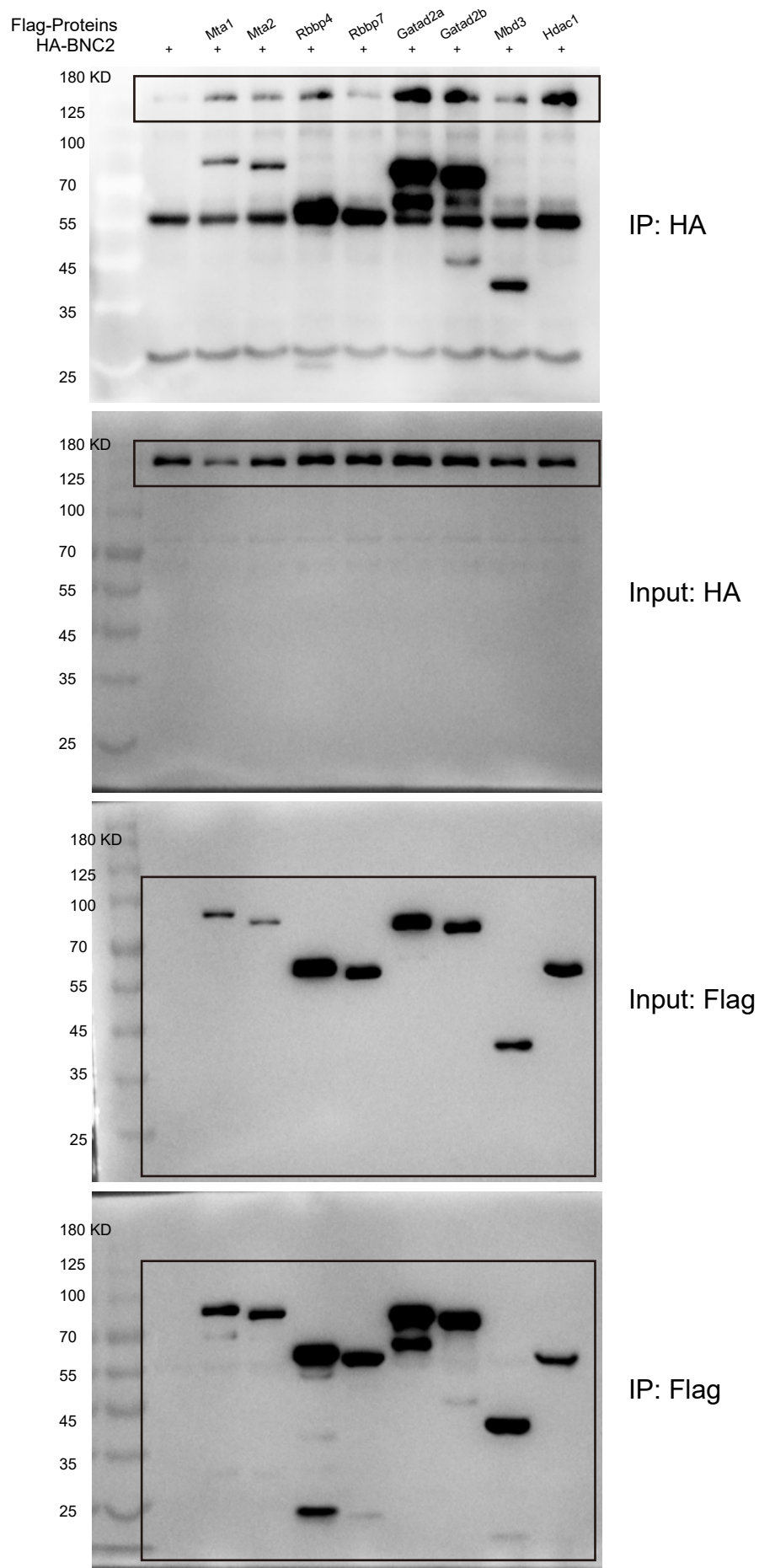

Supplement: Supplementary file 9 — Source data Fig. 7 [file 44318_2025_664_MOESM9_ESM.zip › Figure 7/7C/BNC2 - NuRD IP.pdf]

*Prx1-cre;* *Prx1-cre;*  
*Bnc2*<sup>f/+</sup> *Bnc2*<sup>f/f</sup>

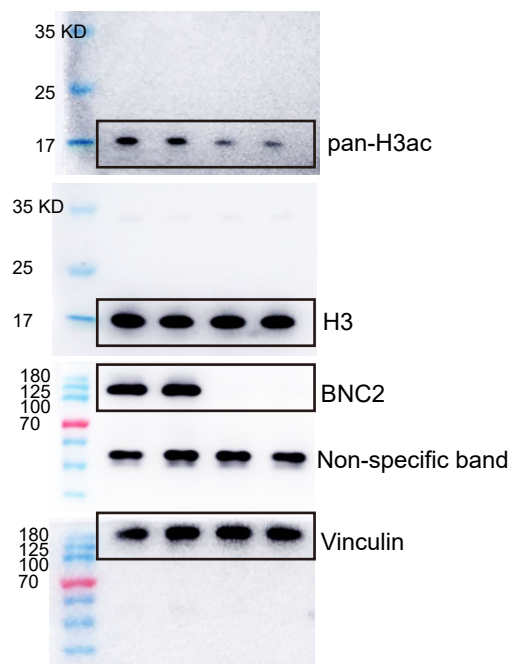

Supplement: Supplementary file 9 — Source data Fig. 7 [file 44318_2025_664_MOESM9_ESM.zip › Figure 7/7D/The H3Ac level in Prx1-cre; Bnc2 mice callus cells was detected by WB.pdf]

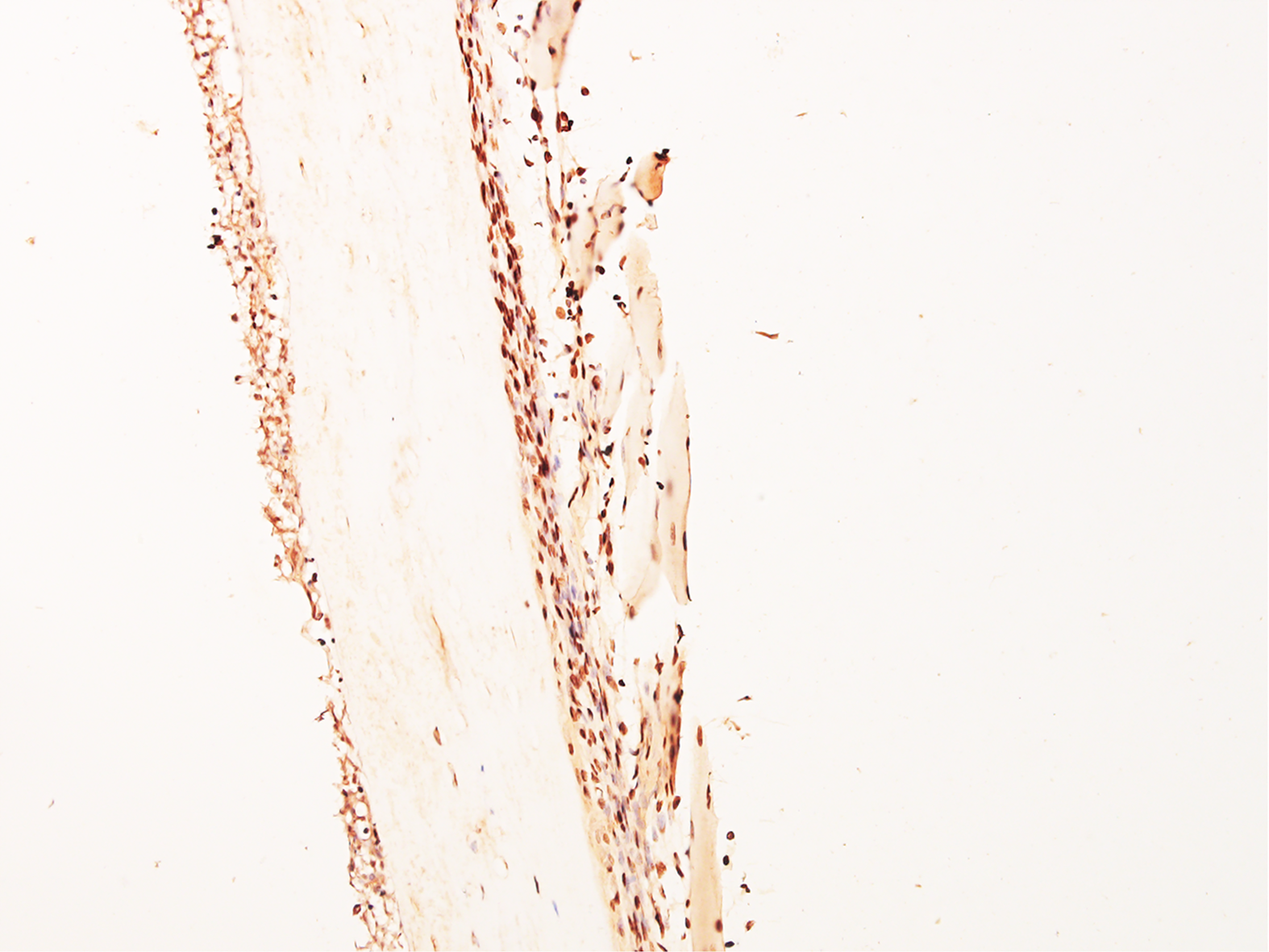

Supplement: Supplementary file 9 — Source data Fig. 7 [file 44318_2025_664_MOESM9_ESM.zip › Figure 7/7E/Ctrl_ pan-H3ac IHC.tif]

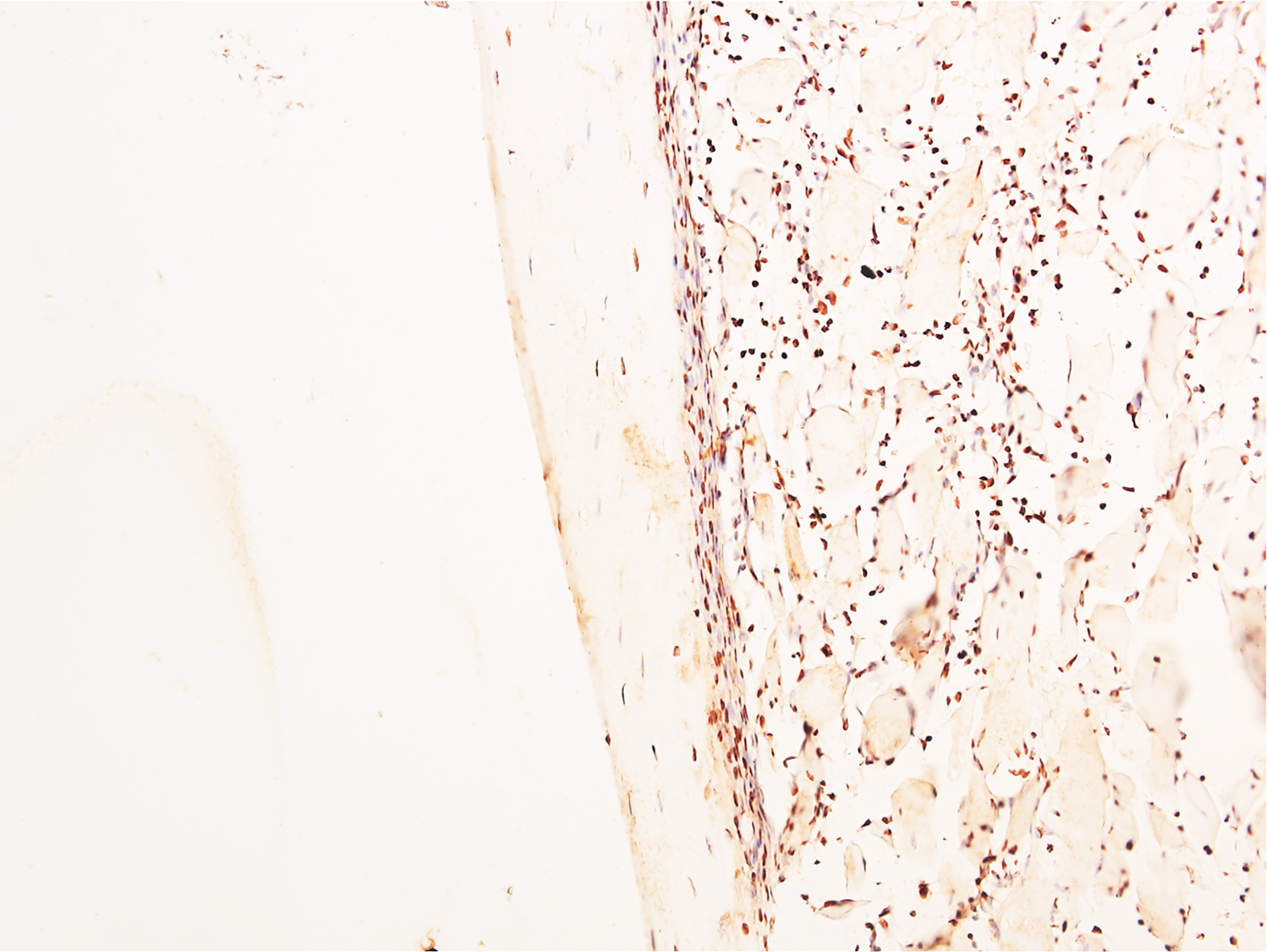

Supplement: Supplementary file 9 — Source data Fig. 7 [file 44318_2025_664_MOESM9_ESM.zip › Figure 7/7E/Prx1-cre;Bnc2_ pan-H3ac IHC.tif]

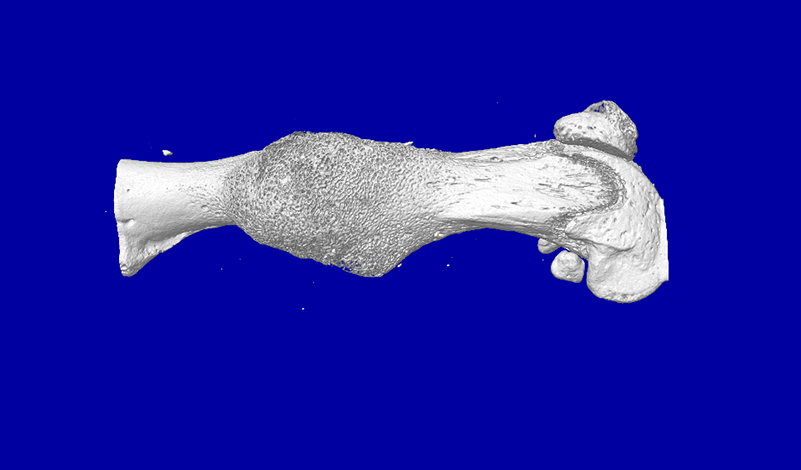

Supplement: Supplementary file 9 — Source data Fig. 7 [file 44318_2025_664_MOESM9_ESM.zip › Figure 7/7H/Bnc2 FF mice + Ctrl.tif]

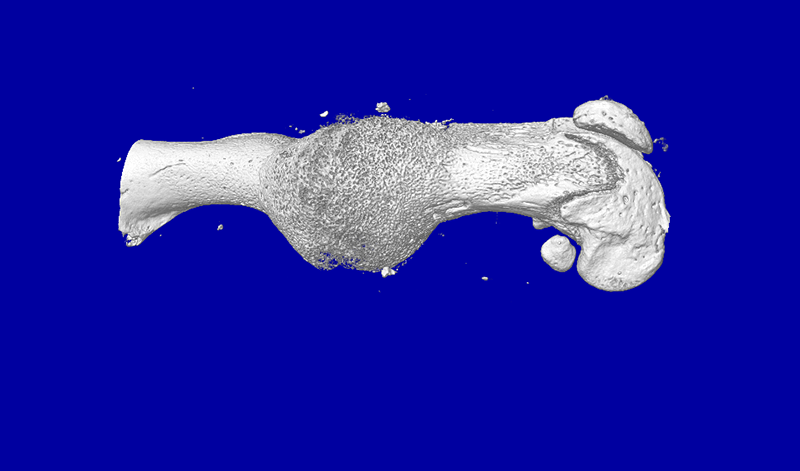

Supplement: Supplementary file 9 — Source data Fig. 7 [file 44318_2025_664_MOESM9_ESM.zip › Figure 7/7H/Bnc2 FF mice + TSA.tif]

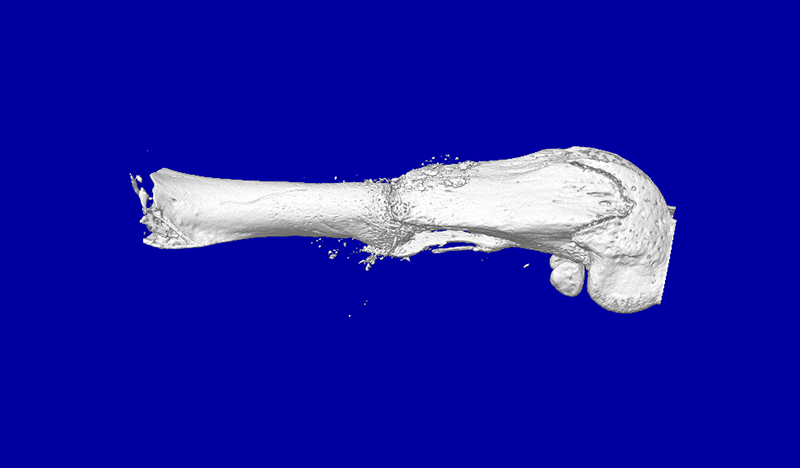

Supplement: Supplementary file 9 — Source data Fig. 7 [file 44318_2025_664_MOESM9_ESM.zip › Figure 7/7H/Prx1-Cre; Bnc2 FF mice + Ctrl.tif]

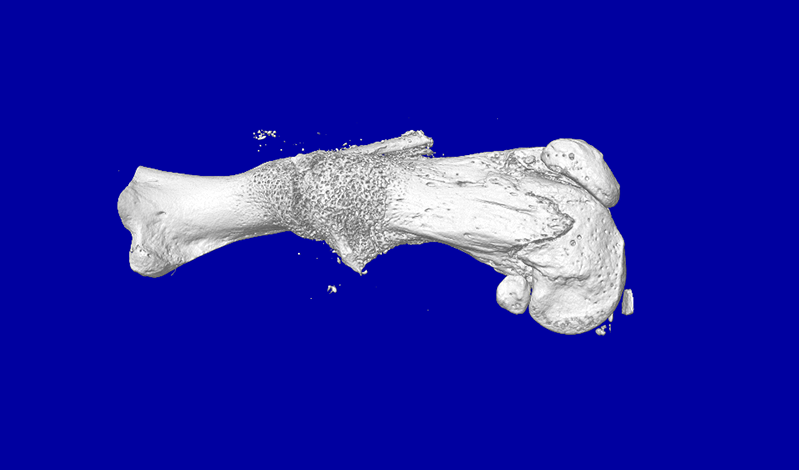

Supplement: Supplementary file 9 — Source data Fig. 7 [file 44318_2025_664_MOESM9_ESM.zip › Figure 7/7H/Prx1-Cre; Bnc2 FF mice + TSA.tif]

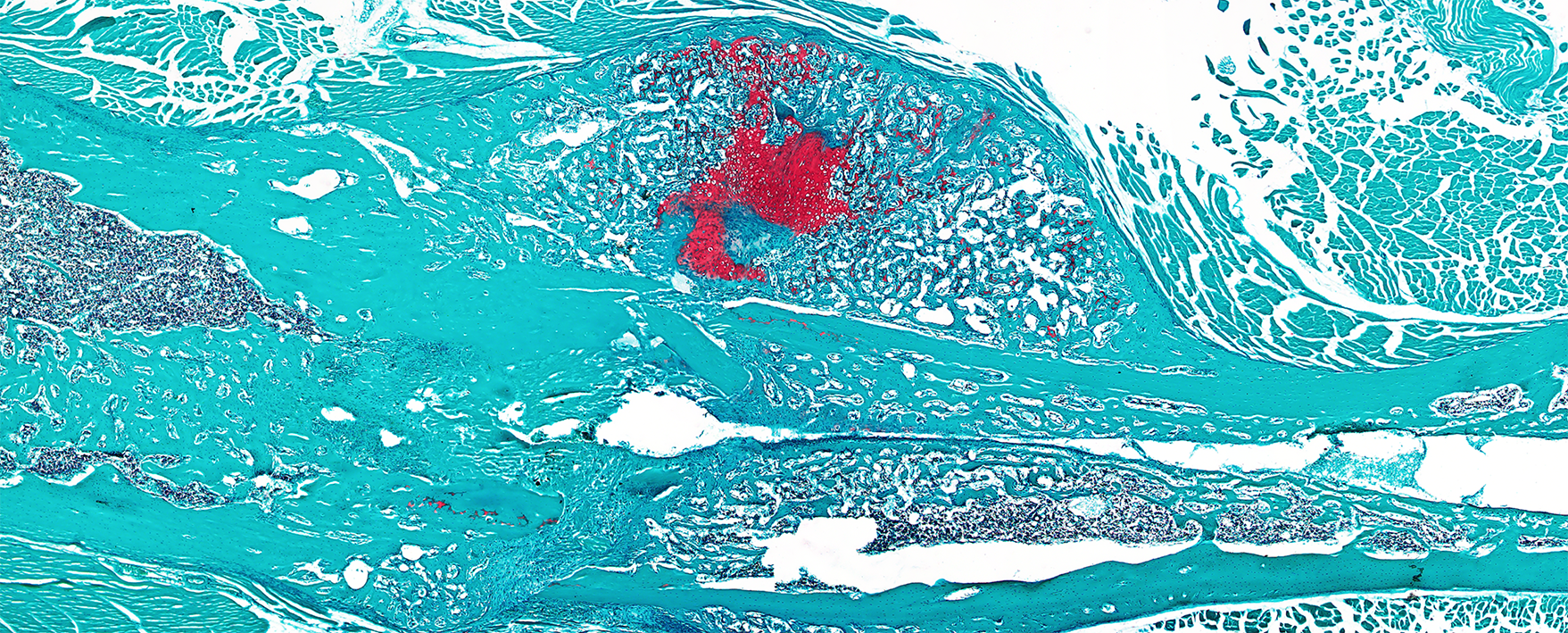

Supplement: Supplementary file 9 — Source data Fig. 7 [file 44318_2025_664_MOESM9_ESM.zip › Figure 7/7J/Bnc2 FF + Ctrl.tif]

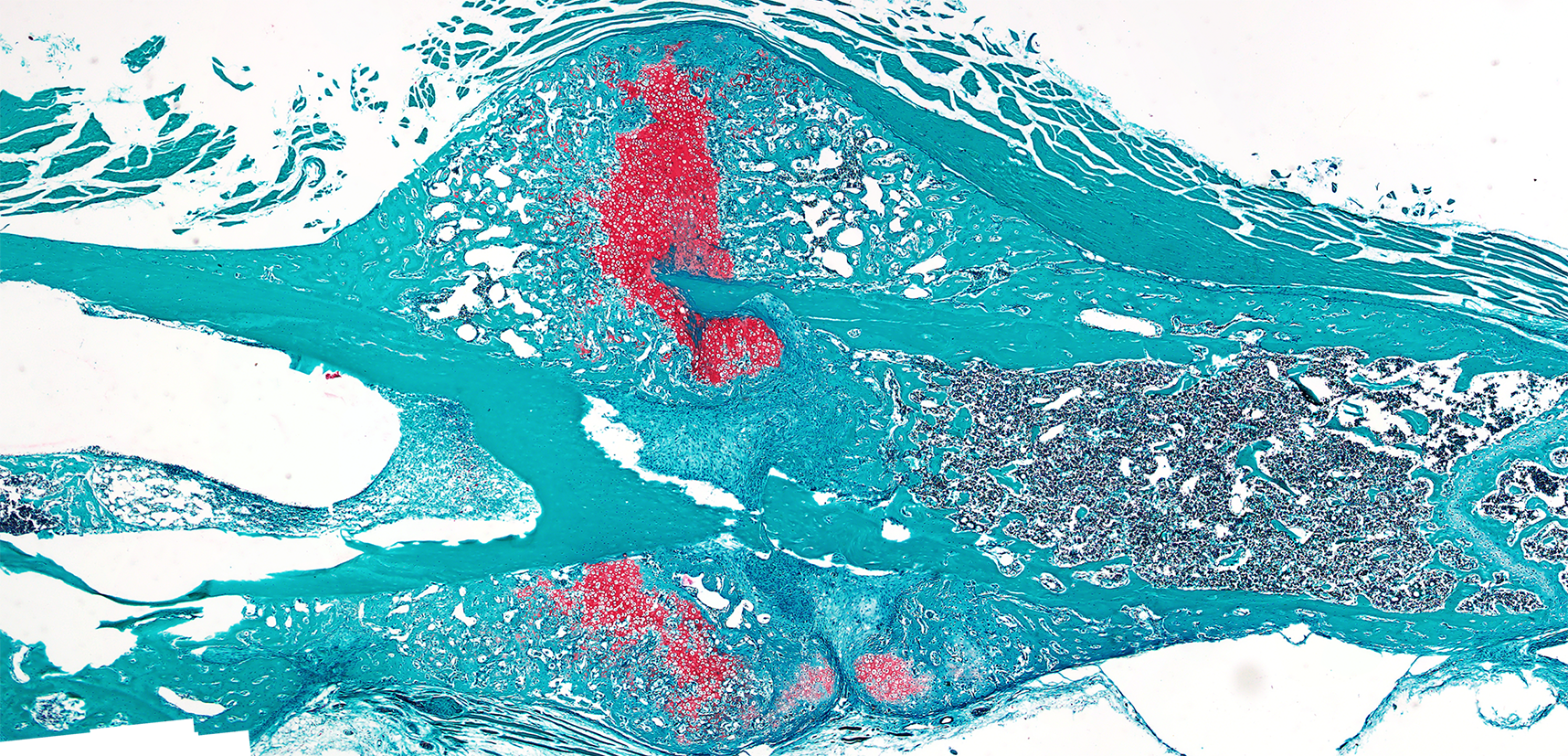

Supplement: Supplementary file 9 — Source data Fig. 7 [file 44318_2025_664_MOESM9_ESM.zip › Figure 7/7J/Bnc2 FF + TSA.tif]

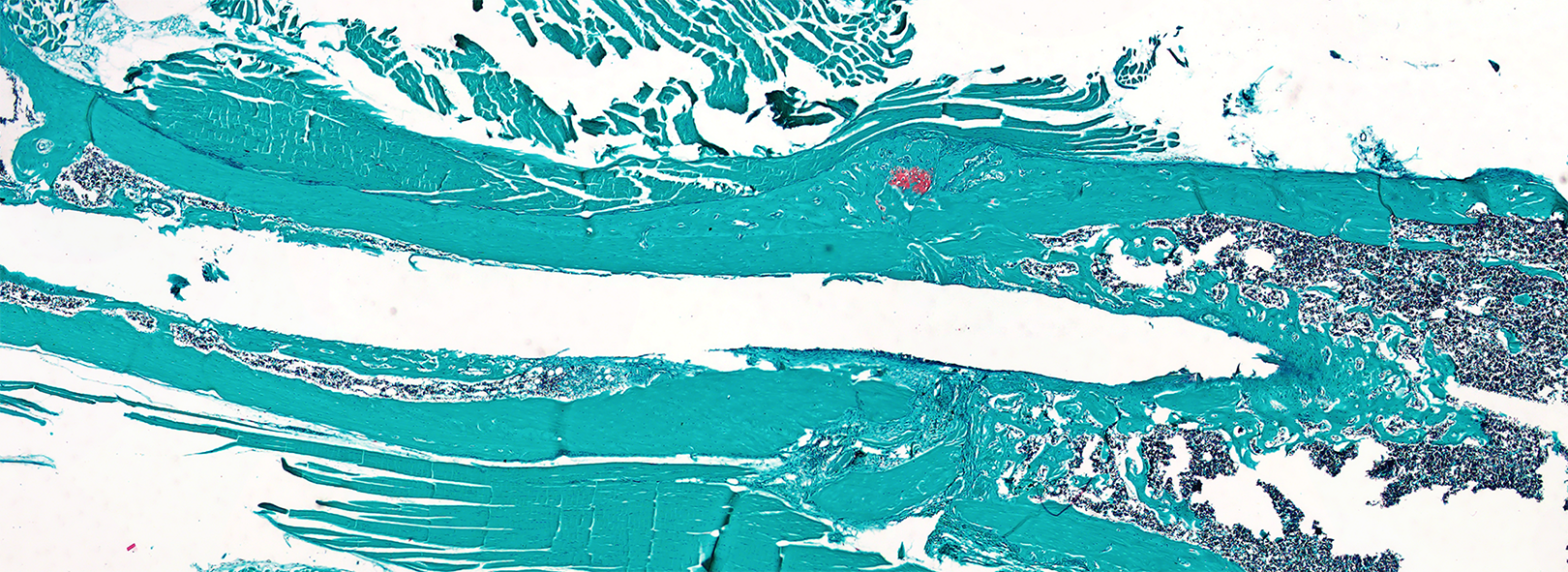

Supplement: Supplementary file 9 — Source data Fig. 7 [file 44318_2025_664_MOESM9_ESM.zip › Figure 7/7J/Prx1-cre; Bnc2 FF + Ctrl.tif]

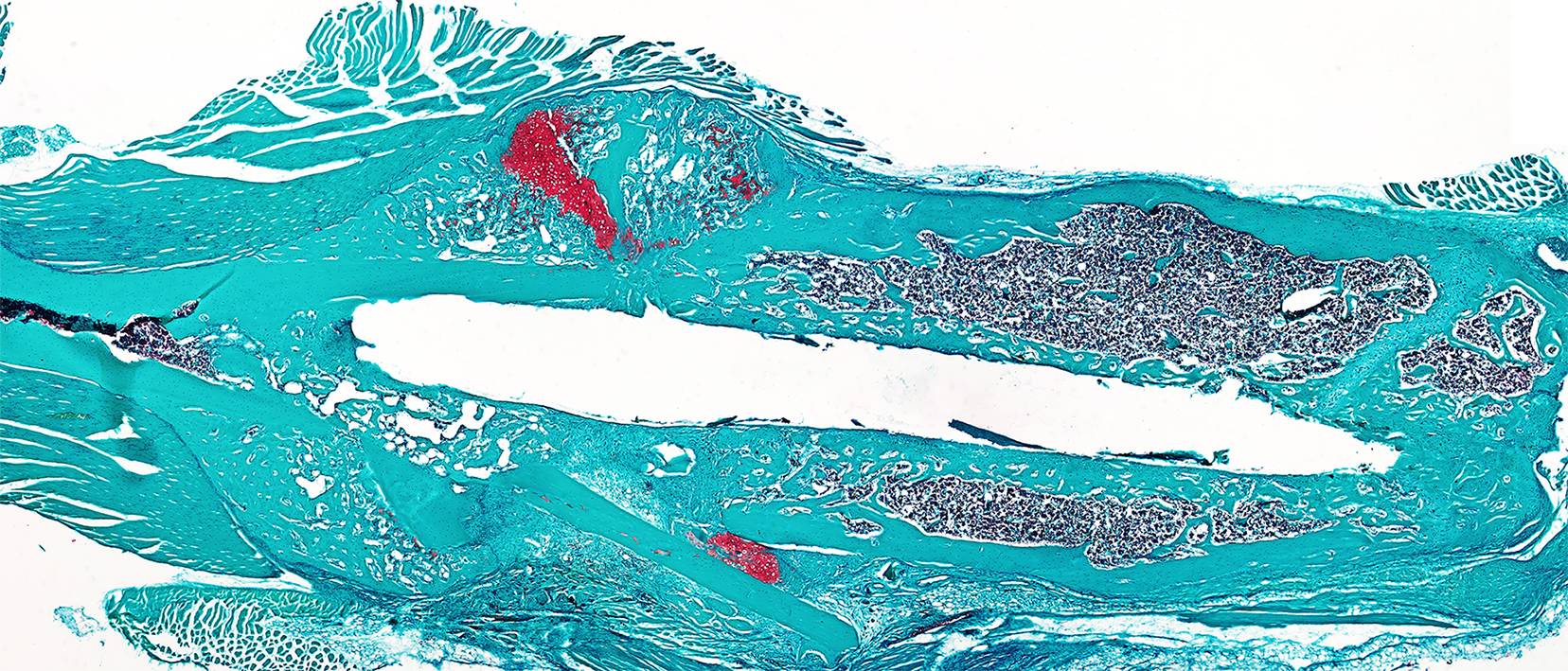

Supplement: Supplementary file 9 — Source data Fig. 7 [file 44318_2025_664_MOESM9_ESM.zip › Figure 7/7J/Prx1-cre; Bnc2 FF + TSA.tif]
